# Multimodal Prodromal Patterns Associated with COPD Exacerbations in Home Telemonitoring: An Exploratory Observational Study

**DOI:** 10.3390/bioengineering13101195

**Published:** 2026-10-14

**Authors:** Daniel Sanchez-Morillo, Patricia Camacho-Magriñán, Antonio León-Jiménez

**Affiliations:** 1 Department of Automation Engineering, Electronics and Computer Architecture and Networks, School of Engineering, University of Cadiz, 11519 Puerto Real, Spain; patricia.camachomagri@uca.es; 2 Instituto de Investigación e Innovación Biomédica de Cádiz (INiBICA), 11009 Cádiz, Spain; antonio.leon.sspa@juntadeandalucia.es; 3 Pulmonology Department, Puerta del Mar University Hospital, 11009 Cádiz, Spain

**Keywords:** COPD, exacerbation, home monitoring, indoor air quality, cough, speech breathing

## Abstract

Characterizing multi-parameter physiological, acoustic, and environmental changes before acute chronic obstructive pulmonary disease exacerbations (AECOPD) is crucial for improving home telemonitoring and enabling timely clinical intervention. In a 12-month prospective observational exploratory pilot study (N=16), a multimodal Internet of Things (IoT) ecosystem collected daily data on symptoms, lung function, cardiorespiratory dynamics, actigraphy, sleep, fine motor skills, bioacoustics, and indoor air quality. From 427 daily features across 4738 patient-days, collinearity mitigation retained 245 independent features. Linear mixed-effects models (LMM), nonparametric paired tests, and multi-timescale exponential moving average smoothing quantified prodromal trajectories and univariate discrimination during the 7-day pre-exacerbation window. Engagement analysis showed a dual hierarchy: passive background monitoring had high retention (>72 %), while demanding active tasks like peak flow spirometry dropped to 26.9 % compliance. Patients with frequent exacerbations showed higher reactive adherence. LMM frameworks identified a multisystem prodromal footprint marked by coordinated increases in cough frequency, sputum purulence, dyspnea, and resting heart rate. Acoustic measures showed notable pre-event changes in higher-order speech cepstral coefficients, voice-intensity variability, and cough spectral dispersion, providing objective proxies for airway limitation. Indoor environmental signals suggested domestic confinement but weakened after adjustment for macro-seasonal confounders. Multi-temporal kinetics demonstrated that acute physiological symptoms peaked within 72 h, whereas acoustic features required 3-to-7-day temporal smoothing (${\mathrm{EMA}}_{3}$–${\mathrm{EMA}}_{7}$) to optimize signal-to-noise ratios. In conclusion, these preliminary observations in the exploratory small cohort suggest that the transition from a stable baseline to the prodromal AECOPD phase is marked by a quantifiable, multi-systemic digital footprint across cardiorespiratory, bioacoustic, and symptom domains. Although requiring validation in broader populations, findings highlight the potential of multi-tiered digital health architectures that pair continuous passive sensing with low-friction active reporting to overcome task attrition and secure the uninterrupted longitudinal baselines needed for automated early-warning systems.

## 1. Introduction

Chronic obstructive pulmonary disease (COPD) is a chronic lung disease characterized by irreversible airflow obstruction. It is a progressive disorder, associated with significant morbidity, disability, and mortality. It affects over 400 million people worldwide and remains the third leading cause of death, inflicting severe health and socioeconomic consequences [1]. COPD imposes a massive and unequally distributed macroeconomic burden across nations, regions, and income levels. Over the 2020–2050 period, the condition is projected to cost the world economy INT$4.326 trillion, with China and the USA bearing the heaviest losses at a combined INT$1.363 trillion [2].

COPD is a heterogeneous lung condition, characterized by persistent symptoms like shortness of breath (dyspnea), cough, and phlegm production. It arises from structural anomalies within the airways, alveoli, and pulmonary vasculature, manifesting as bronchitis, bronchiolitis, or emphysema. Although tobacco smoke and the inhalation of noxious particulate matter or gases from ambient and household air pollution constitute the primary etiological risk factors, the onset of the disease is also modulated by environmental exposures and host vulnerabilities, such as compromised lung development and accelerated pulmonary senescence. COPD typically follows a progressively worsening course and requires confirmation of airflow obstruction through spirometric testing for a definitive diagnosis [3].

The complexity of COPD arises from its progressive and heterogeneous nature, which significantly affects patients’ quality of life, accelerates disease progression, and increases mortality. Despite optimal therapeutic intervention, patients with COPD continue to experience a substantial disease burden. This is primarily driven by recurrent acute episodes, which require significant use of healthcare resources [4]. These episodes, known as acute exacerbations of COPD (AECOPD), are described as episodes marked by dyspnea and/or cough and sputum that worsen over a period of up to 14 days, may be accompanied by tachypnea and/or tachycardia, and are often linked to increased local and systemic inflammation triggered by airway infection, environmental pollution, or other harmful exposures. AECOPD occurs more often as lung function decreases and therefore reflects the ongoing progression of COPD [5].

There is a direct relationship between the severity of COPD and care costs. More than 50% of people diagnosed with COPD experience exacerbations, and healthcare costs in those with frequent exacerbations can be up to six times higher than in patients with clinically stable COPD, primarily as a consequence of increased hospitalisation rates and pharmacological treatment [6]. Beyond these significant economic ramifications, acute exacerbations are linked to elevated morbidity, decreased physical activity and functional mobility, accelerated decline in pulmonary function, and altered sleep architecture. Consequently, these events culminate in reduced health-related quality of life and heightened mortality rates [7]. Studies have reported a substantial risk of recurrence and a notable mortality rate among individuals who suffer severe COPD exacerbations requiring hospitalization [8,9].

In current clinical practice, the detection of exacerbations is based primarily on the patient’s self-report of symptoms, which involves a high degree of subjectivity and habituation and, consequently, leads to a critical delay in treatment. In fact, despite the early detection of COPD exacerbations being crucial to establishing a timely therapeutic window, most patients do not recognise the prodromal signs of an episode and delay seeking medical attention until several days after symptom onset [10], thus prolonging recovery time, reducing health-related quality of life, increasing the risk of subsequent exacerbations and increasing the probability of hospital admission [11,12]. Furthermore, patients often fail to report exacerbations, and evidence indicates that these unreported episodes carry health risks for patients that are comparable to those of reported exacerbations. Multiple factors contribute to underreported and late-reported exacerbations, and addressing them requires a multifaceted strategy [13]. Taking these estimates into account, preventing and treating exacerbations early can reduce the direct costs associated with the disease. In addition, quickly recognising exacerbation symptoms can decrease the likelihood of hospitalisation, allow appropriate and timely therapy, and improve health-related quality of life [11,14].

In this context, patient education, continuous health status monitoring, and efficient exacerbation management have proven essential to improve clinical outcomes and mitigate future episodes, with home telemonitoring emerging as a promising approach in recent years [15].

Recent studies have validated the advantages of telemonitoring for individuals with COPD, showing a marked decrease in the number of exacerbations, as well as a reduction in emergency visits, exacerbation-related readmissions, hospital days, mortality, and symptom burden [16,17]. However, despite substantial research efforts, evidence for the clinical effectiveness of home telemonitoring is still inconsistent [18]. Some systematic reviews have failed to identify evidence for decreases in hospitalisations, emergency department visits, or exacerbation rates [19,20,21]. This lack of consensus underscores the imperative need for more research to clarify the therapeutic value of digital health strategies in the care of COPD. Several critical barriers have been reported to limit the efficacy of home telemonitoring for the early detection of exacerbations, including the lack of reliable predictive algorithms, the scarcity of robust clinical predictors, and low patient adherence.

Regarding predictive algorithms, artificial intelligence (AI) driven COPD exacerbation research is showing promising results, adding a predictive layer to telemonitoring by combining symptom logs, wearables, spirometry data, and environmental information. Nevertheless, the majority of AI-focused studies are based on retrospective data, do not include external validation, and have yet to show measurable benefits in patient-centred outcomes [22].

In terms of clinical predictors, COPD exacerbations do not have a single reliable early warning sign that works well in all patients. Recent advances in remote monitoring highlight the clinical potential of multi-parametric monitoring architectures to enable the early detection of these episodes [23]. In recent studies, the monitored variables cluster into a few recurring groups: symptoms, oxygenation/vital signs, and lung-function measures. While the symptoms side usually includes dyspnea, cough, sputum/expectoration, rescue-inhaler use, and composite symptom scores such as the COPD Assessment Test (CAT) or daily questionnaire scores, the standard set of physiological measurements typically comprises SpO_2_, pulse or heart rate, blood pressure, body temperature, weight and lung-function measurements, including home spirometry variables such as forced expiratory volume in 1 s (FEV_1_), forced vital capacity (FVC), maximum expiratory flow (PEF), inspiratory capacity, or oscillometry/forced oscillation technique (FOT) measures [24,25,26,27]. However, although biomarkers and routine clinical variables have shown potential, their utility is limited by variability, lack of specificity, and inconsistent performance in real-world settings [12]. Developing novel sensors and predictors is crucial to improve the accuracy of current algorithms, which remain limited in detecting the occurrence, severity, and duration of exacerbations [28]. Because nearly half of these episodes lack prodromal signs [29] and patients often do not differentiate isolated poor-health days from actual worsenings [30], the search for clinically effective predictors is a priority for home telemonitoring systems.

In this study, rather than relying on isolated metrics, we propose a digital health framework that integrates longitudinal patient-reported outcomes (symptoms) and physiological information, such as respiratory rate, heart rate, and peripheral body temperature, along with functional markers such as PEF and body weight. These symptoms and physiological data are combined with continuous behavioural and contextual monitoring, including physical activity and sleep data, which can serve as crucial surrogate indicators of functional decline [31,32,33]. Furthermore, we include the passive assessment of indoor air quality metrics, which can provide an essential environmental context [34]. In addition, we explore novel low-burden markers, such as fine motor skill performance, as a potential proxy of systemic or hypoxemia-related fatigue. Similarly, we incorporate the monitoring of advanced acoustic metrics derived from voluntary cough sounds and speech breathing, which could act as surrogate markers for underlying airway inflammation and vocal dynamics [35,36,37]. Integrating this heterogeneous range of markers presents a novel approach with the potential to be used for detecting early warning patterns prior to sudden clinical decline.

In this study, rather than relying on isolated metrics, we propose a digital health framework that integrates longitudinal patient-reported outcomes (symptoms) and physiological vital signs, such as respiratory rate, heart rate, and peripheral body temperature, alongside functional markers like PEF and body weight. This physiological data set is combined with continuous behavioural and contextual monitoring, including physical activity and sleep data, which can serve as crucial surrogate indicators of functional decline [31,32,33]. Furthermore, we include the passive assessment of indoor air quality metrics, which can provide an essential environmental context [34]. In addition, we explore novel low-burden biomarkers, such as fine motor skill performance, to assess systemic or hypoxemia-related fatigue. Similarly, we include monitoring of advanced acoustic metrics derived from voluntary cough sounds and speech breathing, which have the potential to serve as proxies for underlying airway inflammation and vocal cord dynamics [35,36,37]. Collectively, the novel integration of these diverse digital biomarkers offers a promising paradigm for identifying prodromal patterns before acute clinical deterioration occurs.

Considering the above, this exploratory study aimed to contribute to two of the priorities identified for future advancements in COPD telemonitoring: (a) the identification of new physiological and environmental predictors; and (b) the implementation of user-centric strategies that minimise patient burden, thus fostering long-term adherence to the home telemonitoring experience. To do this, we conducted a prospective 12-month observational study in a COPD cohort, extracting 427 daily features per patient. Our focus was twofold: to evaluate the discriminative capacity of these multi-domain features for AECOPD detection; and to analyse how task complexity and physical burden influence patient compliance to provide actionable insights that can optimise the user experience of digital health interventions while preserving the high-resolution data streams required for robust predictive modelling.

## 2. Materials and Methods

### 2.1. Study Design and Study Population

A 12-month prospective observational study was designed to evaluate a multimodal home telemonitoring ecosystem integrating physiological, environmental, symptoms, and bioacoustic data. A cohort of 16 patients with moderate-to-severe COPD was enrolled at the Puerta del Mar University Hospital (Cadiz, Spain). Eligible participants were 40 to 85 years old, clinically stable with a post-bronchodilator FEV_1_/FVC ratio below 70% and presented a history of hospitalisation related to COPD or two exacerbations requiring systemic treatment within the previous year. Inclusion required the ownership of a smartphone with adequate mobile data coverage and sufficient cognitive and motor skills for the independent operation of standard electronic equipment and sensors. Exclusion criteria included patients with active smoking, neoplastic disease, substance abuse, general frailty, or severe comorbidities that limited physical activity or required ongoing antibiotic or glucocorticoid therapy. The Institutional Review Board of the University Hospital Puerta del Mar approved the study (protocol code 29.23, 1 August 2023), and the participants provided their written informed consent, all in accordance with the Declaration of Helsinki.

### 2.2. Telemonitoring Architecture

**Figure 1:**
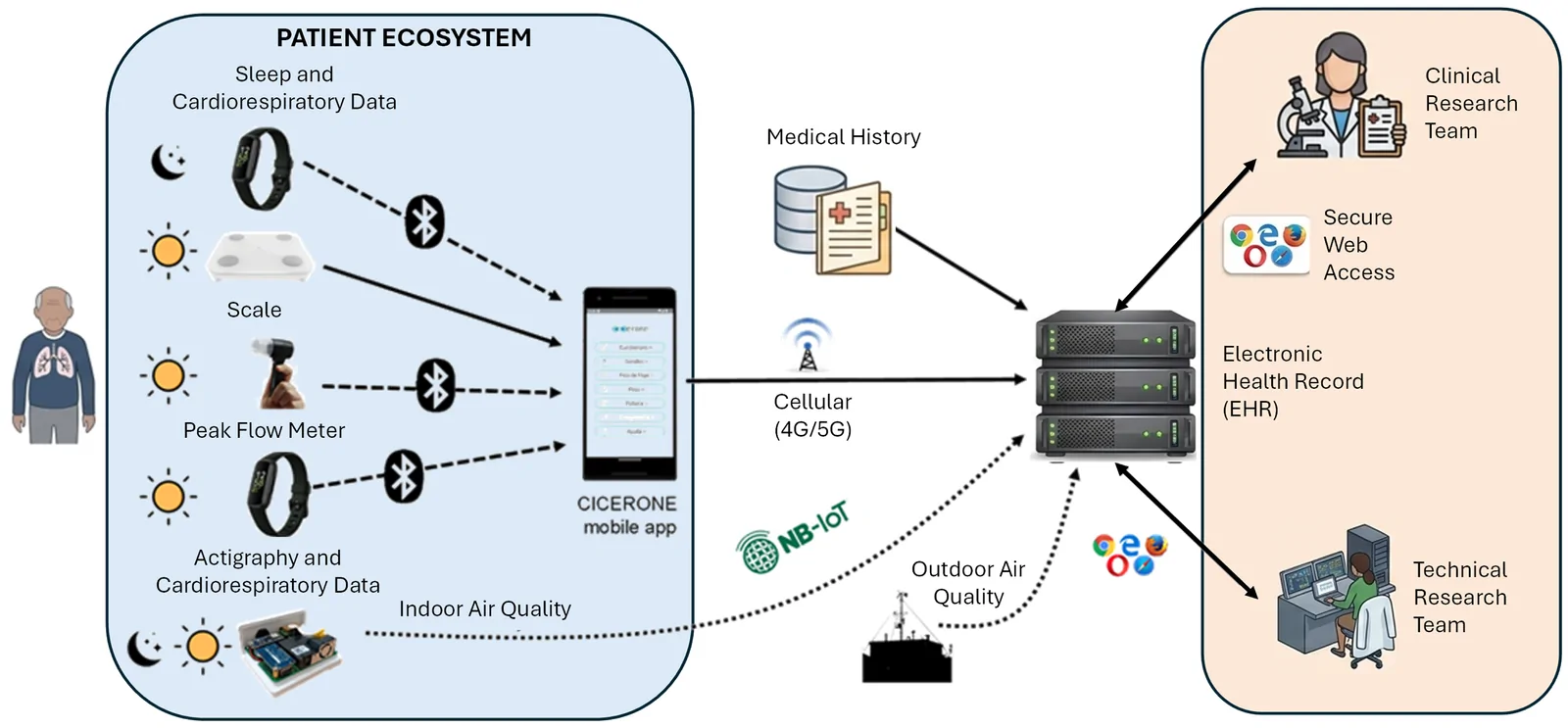
Proposed COPD telemonitoring ecosystem for data acquisition, storage, and clinical access. Data were collected during daytime, nighttime, and over the full day (24 h), as detailed.

A multimodal IoT ecosystem was deployed for data collection, comprising an activity tracker, an indoor air quality node, a scale, a peak flow sensor, and a mobile application designed specifically for COPD patients. The architecture of the system is illustrated in Figure 1.

This infrastructure allowed the collection of both active patient-reported data (symptom questionnaires, PEF, weight, acoustic data, and fine motor tests) and passive measurements (physical activity, cardiorespiratory and sleep metrics, and environmental data).

#### 2.2.1. Mobile Gateway Application (Client-Side)

**Figure 2:**
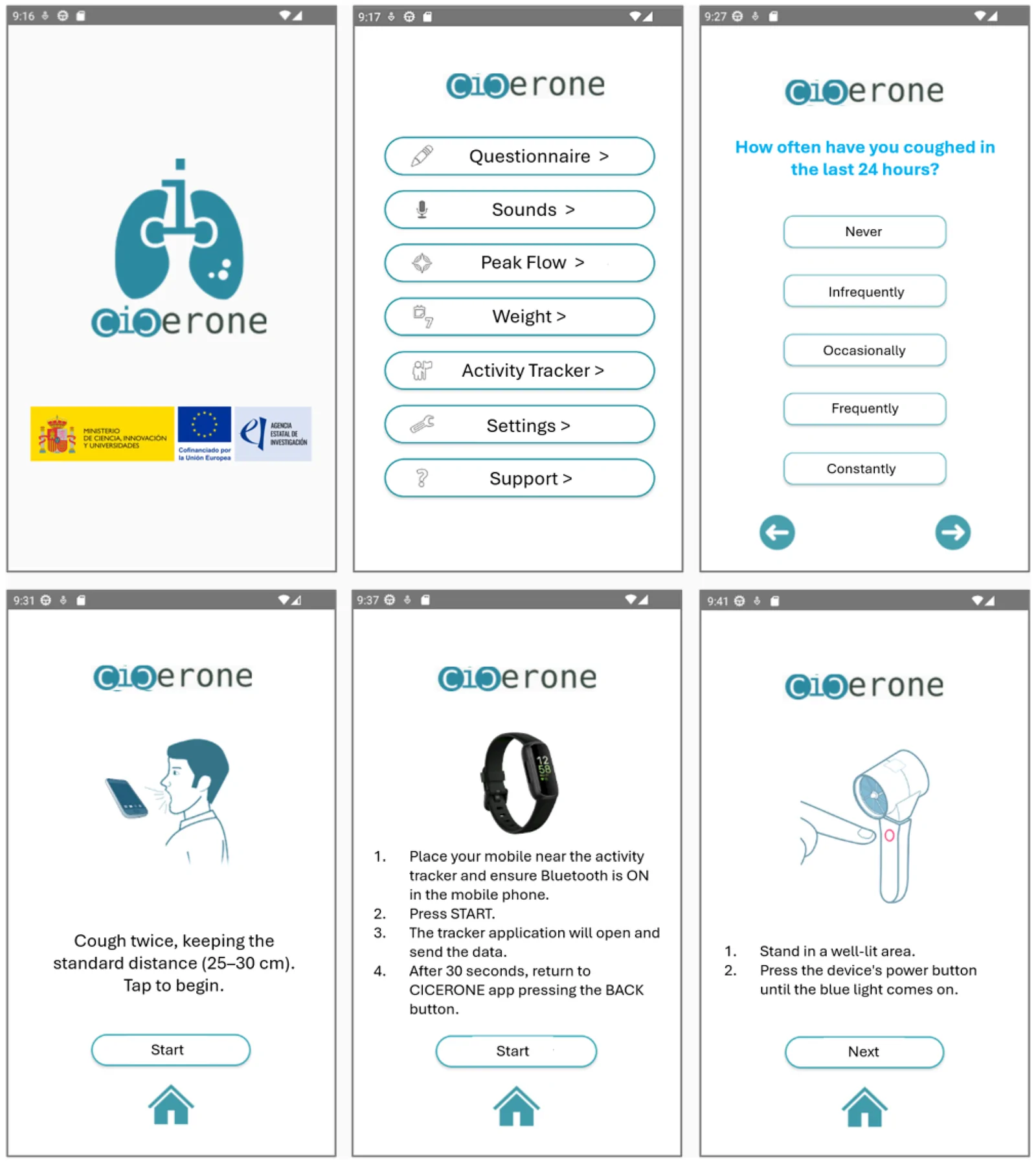
Representative screenshots illustrating the CICERONE mobile application workflow, including symptom assessment, sound recording, and device synchronization.

A mobile application acted as the main gateway, connecting patient-side sensors and a centralised electronic health record (EHR). It was designed using user-centred design principles (UCD) tailored for elderly users (Figure 2) and developed in Java for Android using the Android Studio IDE.

The interface managed structured daily workflows that included a Likert-scale symptom questionnaire, two gamified fine motor tasks, speech and voluntary cough audio recordings, and automated data retrieval from paired sensors. The application was developed following a two-phase usability evaluation (N=18) that achieved an excellent system usability scale (SUS) score of 87.5 [38].

#### 2.2.2. Biomedical and Environmental Sensors Ecosystem

The telemonitoring platform integrated four primary monitoring components:

- Physical activity and sleep: the Fitbit Inspire 3 activity tracker (Google LLC, Mountain View, CA, USA) was used to capture daily activity and sleep metrics, syncing data through Google Health via Bluetooth Low Energy (BLE).
- Body mass: weight fluctuations were captured using the Xiaomi Scale 2 (Xiaomi Corporation, Beijing, China).
- Pulmonary function: PEF was monitored using the Smart Peak Flow wireless spirometer (Smart Respiratory, London, UK) in three guided expiratory manoeuvres.
- Environmental context: custom indoor sensing nodes used narrow-band Internet of things communications (NB-IoT) to continuously measure temperature, humidity, $CO_{2}$ (ppm), volatile organic compounds (VOCs) (index), and particulate matter ($PM_{1},PM_{2.5},PM_{4},PM_{10}$) (μg/m^3^) [39]. These parameters have been associated with AECOPD risk [34,40,41].

#### 2.2.3. Electronic Health Record (Server-Side)

**Figure 3:**
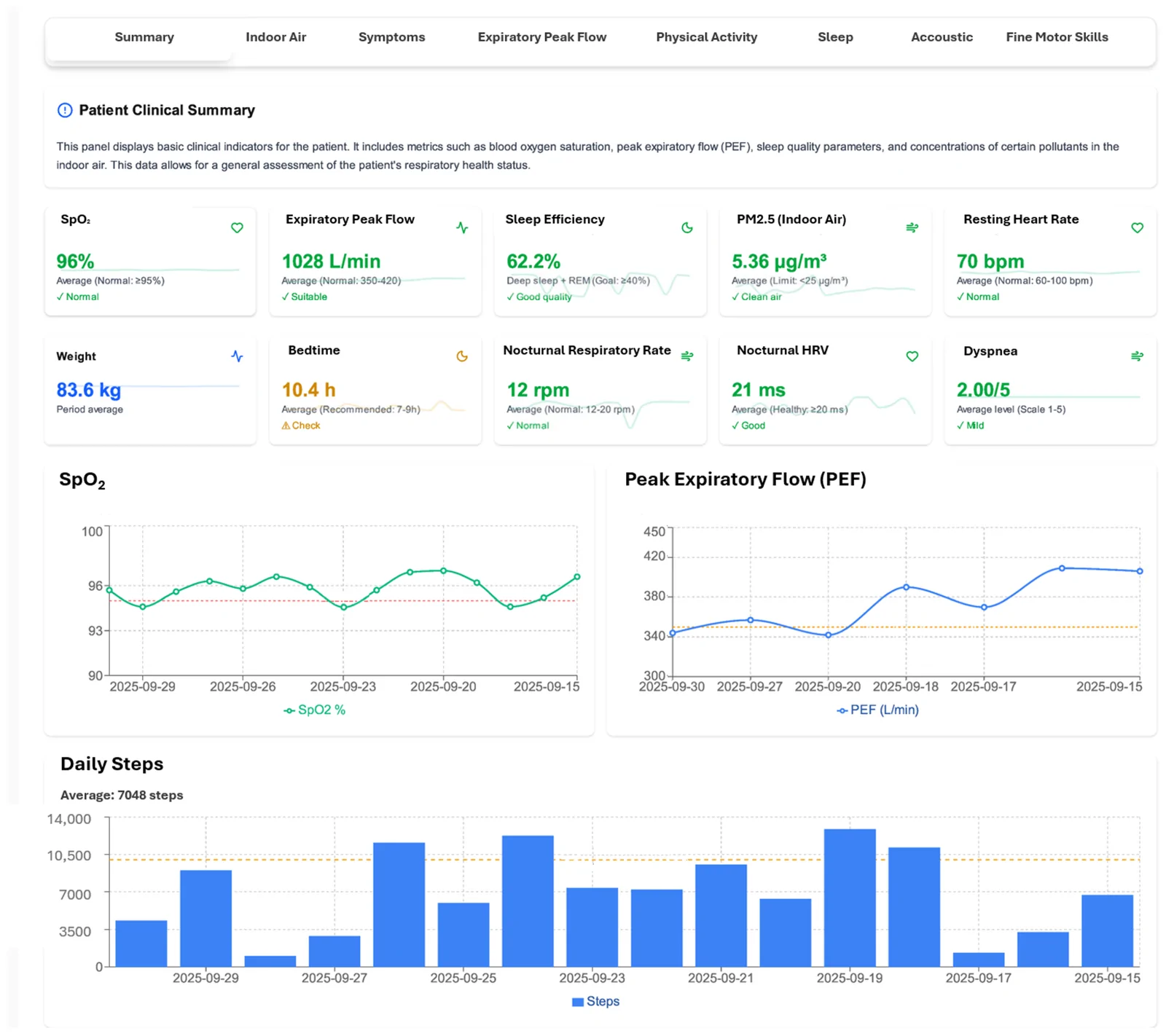
Overview of the clinical dashboard designed for the telemonitoring of the cohort of patients with COPD.

The server-side infrastructure operated as a secure EHR, encompassing patient management modules, environmental and biomedical data intake streams, and analytical dashboards for clinical specialists. Developed using UCD with 6 medical professionals, the dashboard achieved a SUS score of 77.92 [42]. The screenshot showing a summary of clinical data from a patient is illustrated, as an example, in Figure 3.

The backend architecture was designed in Python 3.6 using the Flask microframework to deploy robust RESTful web services. The production deployment was stabilised using a Waitress WSGI application server, backed by SQLite 3.40 database engine. Configuration management and readable structured data interactions were implemented via the PyYAML 6.0 library, utilizing the YAML serialization standard. The registry natively ingested, parsed, and visualized all longitudinal data types.

### 2.3. Daily Monitoring Routine

Participants followed a standardised daily monitoring sequence managed through the mobile application. The routine integrated: (a) a six-item Likert-scale symptom questionnaire covering general health status, dyspnea, rescue inhaler usage, cough frequency, and sputum characteristics; (b) an audio assessment (two voluntary coughs and a standardized phrase reading) for acoustic analysis; (c) two gamified fine motor tasks (target-tapping and circle-tracing) to evaluate psychomotor proficiency; (d) measurement of PEF in three forced expiratory maneuvers; and (e) synchronization with the activity tracker to upload actigraphy, cardiorespiratory, and sleep data. Body weight was recorded weekly. Daily at-home assessments were completed in one uninterrupted session, using the mobile app modules in a predetermined order: (1) self-reported symptom questionnaires and motor control tests; (2) voluntary cough and speech recordings; (3) PEF measurement followed by data synchronization with the tracker; and (4) body weight log.

### 2.4. Multimodal Feature Extraction and Digital Biomarkers

The heterogeneous raw data streams collected by the telemonitoring ecosystem were processed to generate a granular feature space comprising 427 features per patient. Feature dimensions included:

- Environmental metrics: continuous indoor air quality data (temperature, relative humidity, ${\mathrm{CO}}_{2}$, VOCs, and ${\mathrm{PM}}_{1,2.5,4,10}$) were aggregated into 24-h daily profiles and segmented into diurnal and nocturnal cycles. For each variable, four descriptive statistics (mean, standard deviation, maximum, and minimum) were calculated to monitor chronic exposure and acute spikes, summing a total of 96 features per day.
- Wearable physiology and activity: continuous actigraphy tracked steps and intensity levels (sedentary to very active). Autonomic and cardiovascular tone were assessed via resting heart rate and heart rate variability (HRV) quantified using the root mean square of successive differences (RMSSD). Nocturnal respiratory dynamics captured average breathing rates and ${\mathrm{SpO}}_{2}$ extreme values, while sleep architecture metrics parameterized sleep quality and efficiency. Nighttime skin temperature fluctuations were also tracked, yielding a total of 24 daily features.
- Symptoms and lung function: patient-reported outcome measures were captured using a 6-item Likert-scale symptom diary. Lung function was captured using the wireless peak flow sensor, based on the peak flow values obtained from three consecutive maneuvers and their average value. In addition to weekly body weight, a total of 11 features per day were collected for this data dimension.
- Fine motor assessments: two gamified tasks evaluated cognitive-motor proficiency by providing 14 daily features. A continuous circle-tracing task (8 features, including trajectory smoothness and circularity index) was used as a proxy for kinematic smoothness, tremor, and bradykinesia. Visuomotor accuracy and attentional lapses were tracked using a target-tapping task (6 features across three consecutive trials).
- Acoustic markers: acoustic metrics have become potential indicators of respiratory function [43]. In this study, voluntary cough and speech sounds were processed to analyse the differences between the stable and prodromal phases by extracting, in total, 208 features daily. Quantitative characterization of voluntary cough acoustics has emerged as a non-invasive and low-cost screening and tracking tool in obstructive airway diseases such as asthma, COPD, and allergic rhinitis [44]. We examined the microstructural acoustic properties of voluntary cough sounds to assess their potential to characterise the prodromal stage of AECOPD. To standardize cough sound acquisition, participants were instructed to slightly open their mouths, inhale deeply, and perform two to three consecutive maximal-effort coughs. Cough episodes were segmented and analysed to extract 169 temporal, energetic, and spectral descriptors per day. In addition to non-invasively monitoring changes in patient-reported lung function and glottal dynamics, a standardized voice recording task was integrated into the daily telemonitoring protocol. This paradigm leveraged the speech airstream mechanism as a digital proxy for underlying respiratory decline [45]. Thirty-nine acoustic and prosodic parameters were extracted from an audio recording of a standardised, phonetically balanced Spanish sentence read by the patients to assess expiratory control under exertion.
- Temporal feature engineering: to capture longitudinal trajectories and prodromal physiological trends rather than static daily snapshots, an automated time-series vectorization pipeline transformed 15 core baseline variables into 75 engineered features using three mathematical operators for a given variable *x* on day *t*. Comprehensive details regarding the mathematical formulations and extended feature definitions are documented in Supplementary Material S1.

### 2.5. Dataset Creation, AECOPD and Prodromal Window

#### 2.5.1. Outcome Definition

The prodromal phase of COPD is characterized by a progressive subclinical escalation of symptoms that typically unfold over the seven days preceding an acute clinical deterioration [46]. Relying exclusively on hospital registries and administrative records risks underestimating the true rate of exacerbations by up to 50% [47]. Despite remaining unreported, these episodes accelerate cumulative lung function decline and impair long-term health-related quality of life [48].

To mitigate underreporting bias and establish a high-fidelity diagnostic ground truth, a multi-source data fusion strategy was implemented, combining retrospective EHR reviews with patient-reported daily symptom logs.

Exacerbation events were categorized into two operational paradigms: (a) event-based exacerbations, verified through retrospective EHR review and anchored to unscheduled medical encounters accompanied by documented escalation in baseline therapy, in strict compliance with GOLD guidelines [3]; and (b) symptom-based exacerbations, algorithmically identified from daily symptom diaries using Anthonisen criteria, which require an acute deterioration of at least two symptoms among sputum volume, sputum purulence, and dyspnea for two consecutive days [49].

When symptom-based detections and clinical events occurred in close temporal proximity, they were consolidated into a single composite AECOPD episode to avoid redundant window allocation and conflicting class labels. The target variable was defined as follows: (i) the prodromal phase spanned the 7 days before initial symptom onset and extended continuously through all intermediate days up to the acute clinical event; (ii) the acute phase covered 14 days initiated at the onset of the index clinical event (or the later event in composite cases); and (iii) the baseline phase comprised all remaining days of clinical stability. Consequently, the primary target outcome was defined as a binary classification variable (Y∈{0,1}). Days corresponding to the biological onset of an exacerbation, along with their 7-day preceding prodromal windows, were designated as risk days (coded as “1”), whereas all homeostatic monitoring periods were designated as stable baseline days (coded as “0”). The acute phase periods (−1) were strictly excluded from this binary classification.

#### 2.5.2. Recovery Period Exclusion Criteria

Fluctuations in symptoms throughout the recovery period are well recognized and may interfere with the validity of statistical modeling [50]. Although median symptom recovery is reported at seven days (IQR 4–14) [46], this implies that half of all episodes remain unresolved beyond the seventh day. Recovery timelines vary considerably, with a mean of 11 to 13 days depending on the exacerbation phenotype [29], with systemic inflammation and CAT scores reaching baseline at approximately 14 days [51]. Consequently, as an operational data-cleaning rule to ensure data integrity and prevent signal contamination from incomplete recoveries, a conservative 14-day post-onset period was strictly excluded from baseline modeling.

### 2.6. Data Curation and Preprocessing Pipeline

To ensure data integrity and clinical validity, a systematic data curation and quality-control pipeline was applied to individual patient datasets prior to statistical analysis. Records with missing or corrupt temporal entries were excluded. First, physiological and environmental features were passed through clinical sanity filters and a subject-specific interquartile range (IQR) thresholding routine to remove extreme outliers and technical artifacts. For each continuous marker, first ($Q_{1}$) and third ($Q_{3}$) quartiles were calculated independently per patient; values falling outside $\left[Q_{1}-3.0\times \mathrm{IQR},Q_{3}+3.0\times \mathrm{IQR}\right]$ were identified as anomalous and mapped to missing values (NaN).

Following outlier removal, individual data streams were vertically integrated into a unified longitudinal repository. The composite target variable was then chronologically engineered for each patient by synchronizing clinical events, symptom-based worsenings, and enforcing the 14-day recovery exclusion window (Section 2.5.1 and Section 2.5.2).

Finally, a hybrid imputation algorithm was deployed to handle remaining missing sequences without introducing synthetic artifacts:

- Last observation carried forward (LOCF) was applied to highly stable parameters or patient-reported clinical status metrics (e.g., body weight and daily symptom scores).
- Linear interpolation was applied to dynamic biosignals and time-varying markers (e.g., physical activity tracking, heart rate variability, sleep efficiency, environmental sensors, ambient air quality, spirometry peak flow, and speech acoustics metrics) to model intra-day trends. To avoid artificial data fabrication during prolonged system disconnections, a continuity cap was enforced, limiting linear interpolation to a maximum consecutive gap of 3 days. Missing sequences exceeding this boundary remained unimputed.

The 4 data points derived from the daily peak flow test were excluded from the primary analysis. A combination of low active patient compliance and technical transmission failures created significant structural missing data within this domain, which could have compromised statistical power and introduced bias into the longitudinal models. Consequently, out of the 427 total features per day, a pool of 423 markers spanning 10 clinical domains was retained for downstream statistical analysis.

### 2.7. Statistical Analysis and Model Evaluation

#### 2.7.1. Patient Adherence to Telemonitoring Tasks

To address the dichotomy of telehealth adherence between low-effort passive monitoring and demanding active self-reporting [25,52], this study quantified compliance based on task complexity. To evaluate participant burden, monitoring tasks were stratified by intensity of interaction: passive (continuous background sensing such as indoor air quality, physical activity, and sleep monitoring) and active (moderate cognitive load, such as questionnaires and motor tasks, and instrument-based physical effort, such as peak flow manoeuvres, body weight, and voice recordings).

For each participant and each monitoring modality, overall adherence was defined as the proportion of compliant monitoring days out of the total follow-up duration for that subject. Cohort-level metrics were summarized using a macro-averaging approach, wherein individual patient adherence percentages were calculated and averaged across the cohort (mean±standarddeviation). Median and IQR were estimated to reflect between-patient dispersion. To evaluate the association between exacerbation burden and monitoring compliance, the total count of AECOPD experienced by each participant during the observation period was quantified. Bivariate relationships between patient-level AECOPD frequency and modality-specific adherence rates were assessed using the Spearman correlation. Furthermore, participants were stratified into exacerbation subgroups (low-frequency: ≤2 events; high-frequency: ≥3 events) to perform group-wise comparative evaluations of mean adherence across active and passive monitoring domains.

#### 2.7.2. Feature Selection and Collinearity Mitigation

To mitigate multi-systemic collinearity and satisfy the mathematical assumption of feature independence, a feature selection framework was implemented before inferential testing. Longitudinal bivariate correlations were evaluated using the repeated measures correlation (rmcorr) technique. This collinearity analysis was performed using the longitudinal data derived strictly from the stable baseline phases of the patient. The rationale for isolating the correlation analysis to the stable period is twofold: first, it establishes an unconfounded baseline of the patient’s multimodal ecosystem, capturing the intrinsic physiological, behavioral, and environmental cross-correlations without the mathematical distortion introduced by acute clinical exacerbations. Second, computing rmcorr during a homeostatic state prevents artificial inflation or masking of correlations caused by systemic disease-driven trajectories, thereby ensuring that characteristic flags for high collinearity (|rmcorr|≥0.75) reflect genuine baseline redundancies rather than shared responses to clinical deterioration. For every highly correlated pair identified during this baseline assessment, an objective optimization rule was applied, retaining exclusively the marker that demonstrated the highest univariate discriminative performance, measured using the area under the receiver operating characteristic curve (ROC-AUC).

#### 2.7.3. Univariate Assessment of Prodromal Phase Shifts

Non-parametric Wilcoxon signed-rank tests for paired measures were conducted to validate the magnitude and significance of changes in markers between the baseline and prodromal phases without assuming normality. To control for the unbalanced number of daily observations per phase, longitudinal data was first aggregated by computing the mean value of each marker per patient within each clinical status (stable vs. prodromal). To complement asymptotic *p*-values and quantify the clinical and discriminative strength of each physiological, environmental, and acoustic marker, robust effect sizes were calculated. Specifically, the common language effect size (CLES) [53], which expresses the probability that a randomly selected patient will exhibit a higher marker value in the prodromal phase compared to their stable baseline, was calculated, ensuring a distribution-free interpretation of the physiological shifts.

To control the false discovery rate (FDR) arising from multiple hypothesis testing across candidate features, *p*-values were adjusted using the Benjamini–Hochberg procedure applied independently within each monitoring domain. Features with FDR-adjusted *q*-values below 0.05 (q<0.05) were considered statistically significant, whereas biomarkers with 0.05≤q<0.10 were retained and presented as exploratory or suggestive trends.

#### 2.7.4. Linear Mixed-Effects Models

Linear Mixed-Effects Models (LMMs) were implemented to compare feature dynamics during the prodromal phase against the stable baseline state, controlling for inter-subject variability and the intrinsic correlation of repeated measurements within individuals. To preserve between-subject heterogeneity while accounting for the hierarchical data structure (daily longitudinal observations nested within patients), all continuous variables were standardized on a global scale across the entire cohort. Models were specified with a patient-specific random intercept ($\sigma_{\mathrm{intercept}}^{2}$) to capture baseline subject-level clustering. Where numerically stable and convergent, a random slope for prodromal status ($\sigma_{\mathrm{slope}}^{2}$) was incorporated; otherwise, a random-intercept-only structure was retained.

Variance components, comprising the random-intercept variance ($\sigma_{\mathrm{intercept}}^{2}$), random-slope variance ($\sigma_{\mathrm{slope}}^{2}$, where applicable), and residual variance ($\sigma_{\mathrm{residual}}^{2}$), as well as the intraclass correlation coefficient (ICC), were systematically calculated.

Residual model assumptions were evaluated through visual diagnostics and distributional summary statistics. Formal hypothesis tests for normality (such as the Shapiro–Wilk test) were omitted because of the large sample size.

Model estimation was performed by maximizing the restricted maximum likelihood (REML) log-likelihood function using Powell’s derivative-free conjugate direction algorithm. This optimizer was selected to ensure robust numerical convergence across the high-dimensional feature space and to prevent optimization failures near variance parameter boundaries. To account for multiple hypothesis testing across candidate markers, *p*-values associated with the prodromal status fixed effect were adjusted using the Benjamini–Hochberg FDR procedure at both domain and global levels. Statistical significance was defined as q<0.05, whereas 0.05≤q<0.10 was considered an exploratory trend.

To control for seasonal confounding, calendar season was incorporated as a fixed covariate across all LMM frameworks. Unadjusted and seasonally adjusted standardized parameters (β coefficients, 95% CIs, *p*-values, and domain- and global-specific FDR *q*-values) were systematically evaluated to quantify feature stability and signal attenuation.

##### Reliability of Fine Motor Features

Finally, the test–retest reliability of fine motor features during clinically stable periods was assessed using LMM analyses restricted to days categorized as stable. To account for unbalanced longitudinal daily observations per participant, LMMs with participant-specific random intercepts were fitted to estimate variance components. For each feature, the ICC was computed as the ratio of between-subject variance to total variance. Additionally, the standard error of measurement (SEM) and the mean within-subject coefficient of variation (CV) were calculated to quantify absolute precision and daily performance variability.

#### 2.7.5. Univariate ROC Analysis

The focus of this evaluation is descriptive and exploratory, quantifying the individual discriminative capacity and magnitude of signal separation between baseline stable days and prodromal days across candidate features. To eliminate baseline inter-individual heterogeneity, a patient-specific Z-score transformation was first applied to each marker time series, standardizing daily measurements relative to each subject’s individual mean and standard deviation. To evaluate how tracking short-term longitudinal trends impacts discriminative power and mitigates daily measurement noise, a temporal trend smoothing analysis was systematically implemented. Exponential moving averages (EMA) were calculated for retrospective intervals of 3 days (${\mathrm{EMA}}_{3}$) and 7 days (${\mathrm{EMA}}_{7}$) for each feature of each participant throughout the continuous longitudinal series. Univariate ROC-AUC metrics were computed for each raw feature as well as its corresponding smoothed variants (${\mathrm{EMA}}_{3}$ and ${\mathrm{EMA}}_{7}$). To account for opposite physiological response directions (i.e., features that pathologically increase versus those that decrease during prodromal deterioration), AUC metrics were directionally aligned to express absolute separation performance (ROC-AUC≥0.50). These univariate metrics serve as standardized effect-size indicators of physiological signal shifts, not as multivariate measures.

#### 2.7.6. Sensitivity Analysis

To address potential incorporation bias arising from the dual use of patient-reported symptoms as both candidate predictive features and criteria for symptom-defined exacerbations (Anthonisen criteria), a secondary sensitivity analysis was conducted. We defined a restricted, hard clinical outcome consisting exclusively of EHR-verified exacerbation events. All primary multivariable LMMs and feature discrimination pipelines (ROC-AUC performance across EMAs) were re-estimated against this independent clinical endpoint. Signal robustness was evaluated by comparing the stability of regression coefficients (β), domain-level FDR-adjusted *q*-values, and ROC-AUC metrics between the primary composite outcomes and the hard EHR-verified clinical events.

### 2.8. Software

Signal processing, graphical representation, and statistical analyses were performed using Python 3.11. LMMs were implemented via the statsmodels library, while non-parametric analyses and repeated measures correlations were conducted using the packages SciPy and Pingouin, respectively.

## 3. Results

### 3.1. Participants

Table 1 summarizes the demographic and clinical features of the participants at the time of enrollment. The study cohort comprised 16 subjects, with a predominantly male population (68.8% male, 31.2% female) of a mean age of 67.2±6.1 years old and a mean body mass index (BMI) of 28.5±5.9${\mathrm{kg}/m}^{2}$.

**Figure 4:**
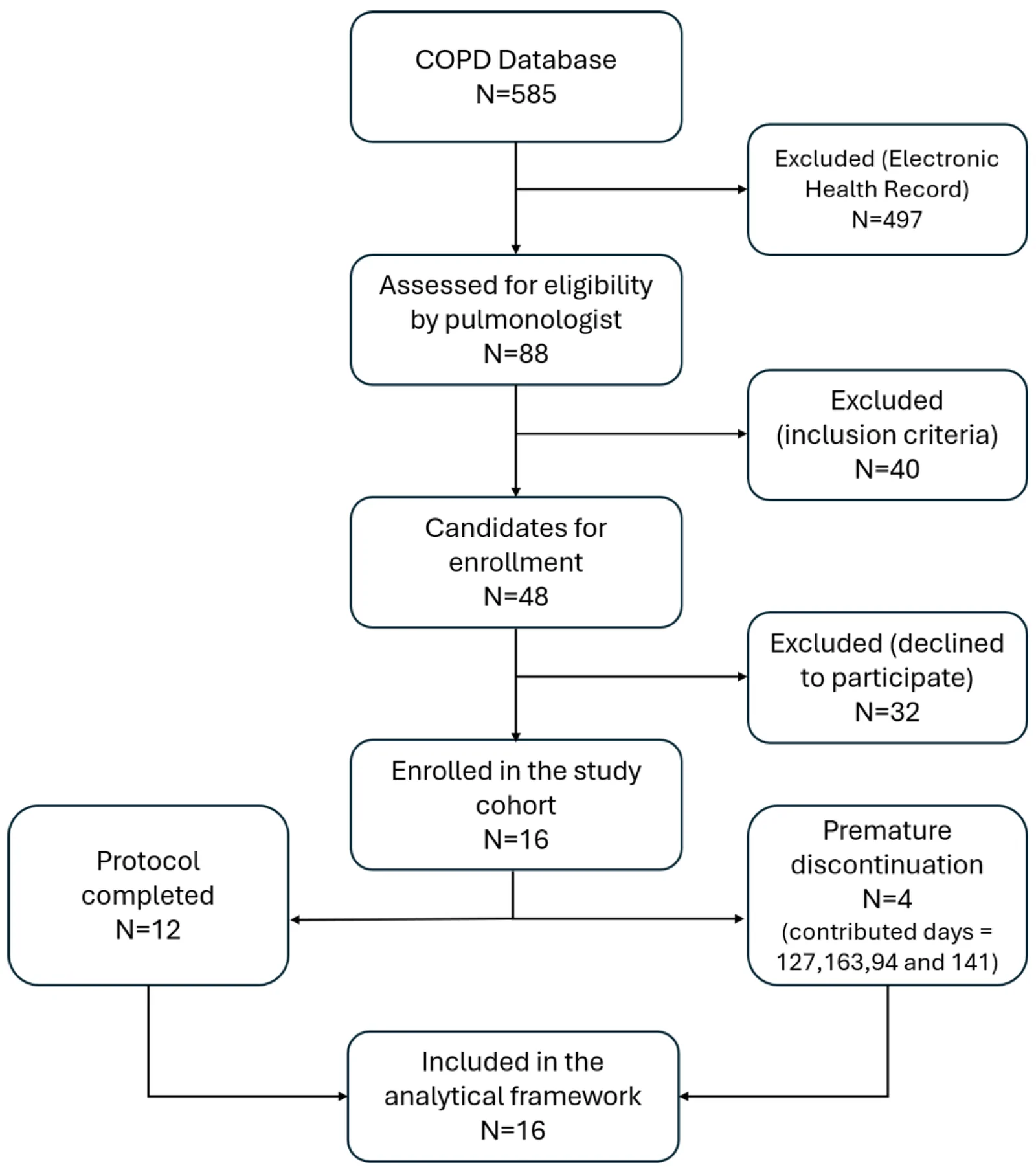
STROBE diagram for the study (STROBE: Strengthening the reporting of observational studies in epidemiology).

The cohort was recruited using a staggered enrolment design between October 2024 and June 2025, according to a study protocol that required a minimum active telemonitoring period of 6 months per participant. Figure 4 shows the diagram that represents the phases of the study.

Four participants (25%) were classified as premature dropouts by failing to complete their follow-up period. Although individual primary reasons were not systematically recorded through formal exit questionnaires, the contributing factors identified included relocation outside the study site, replacement of smartphones that caused loss of connectivity, and voluntary withdrawal from daily self-reporting.

Despite their withdrawal, these participants contributed between 94 and 163 active monitoring days along with verified baseline and prodromal records. The remaining 12 participants (75%) successfully adhered to the long-term protocol, with active monitoring windows ranging from 260 to 471 days by the time the study was completed in February 2026. Consequently, all 16 participants were retained in the primary multivariable LMMs, while 15 patient pairs were evaluated in the secondary paired Wilcoxon analyses, given that a participant without exacerbation events was excluded from the paired testing.

### 3.2. Dataset

The study yielded a total of 4738 cumulative active telemonitoring days across all participants. The individual breakdown of detected AECOPD episodes, overlapping events, and valid prodromal windows is detailed in Table 2. In total, 33 event-based and 56 symptom-based episodes were recorded. Methodological consolidation criteria, including overlapping definitions, occurrence at study baseline without a preceding 7-day monitoring window, or events presented without sufficient post-event stabilization days, led to a total of 43 excluded events. This process yielded 46 prodromal windows available for statistical analysis, with the number of eligible windows per participant ranging from 0 to 6.

Following the removal of 14-day post-exacerbation recovery periods (1155 patient-days excluded), a total of 3583 patient-days were included in the final analytical dataset. These were categorized into 393 prodromal days and 3190 stable baseline days. This established a class imbalance ratio of 8.12:1 (stable-to-prodromal days).

**Figure 5:**
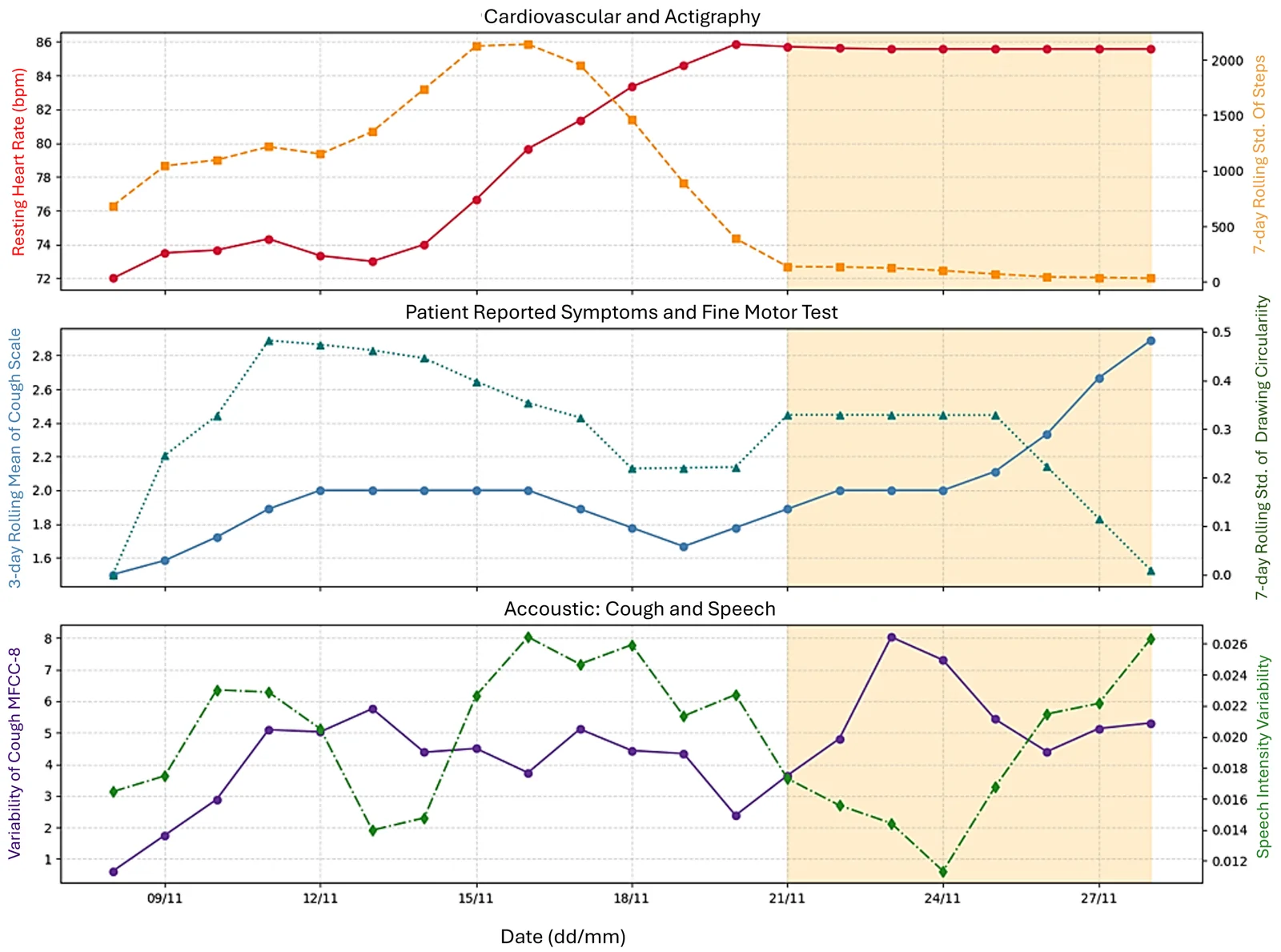
Multimodal trajectory of some representative physiological, behavioral, and acoustic parameters for subject P2 during a prodromal period. The top panel displays resting heart rate and the 7-day rolling standard deviation of step count. The middle panel shows the reported cough scale alongside variability in the fine motor circularity drawing test. The bottom panel presents the variability of the MFCC-8 spectral coefficient of cough and the speech intensity variability. The shaded area delineates the 7-day prodromal window preceding the exacerbation event.

As a representative example, Figure 5 illustrates the multimodal trajectory of some physiological, behavioral, and acoustic parameters before the onset of an AECOPD episode.

### 3.3. Patient Adherence to Telemonitoring Tasks

Table 3 shows the Spearman correlation and average adherence according to exacerbation frequency by data modality. Across the entire cohort (N=16), overall adherence varied substantially depending on the nature of the data collection modality. Passive monitoring yielded the highest levels of compliance, led by daytime physical activity monitoring (83.9%), nocturnal physiology tracking (74.5%), and continuous indoor air quality monitoring (72.2%). Conversely, active patient-reported tasks requiring explicit daily engagement demonstrated lower overall adherence rates. Daily symptom questionnaires with fine motor tests reached a mean adherence of 51.1%, followed by cough audio recordings (49.1%) and speech phrase recordings (47.6%). The lowest compliance rates were observed for daily PEF measurements (26.9%) and body weight logging (25.4%). Across all modalities, overall mean adherence per participant reached a cohort median of 54.0% [47.3–64.5%].

Furthermore, exacerbation frequency was positively correlated with adherence across multiple domains, showing moderate-to-strong associations particularly for indoor air quality tracking (ρ=0.61), daily peak flow measurements (ρ=0.55), and symptom reporting (ρ=0.40). Subgroup analysis corroborated this trend, revealing that participants with a high exacerbation burden (≥3 AECOPD) consistently maintained higher compliance rates across all modalities compared to low-frequency exacerbators (≤2 AECOPD).

#### Reliability of Fine Motor Features

In this exploratory analysis, fine motor features appeared to display varying reliability trends across stable days depending on their operational domain. Temporal execution speed (tapping_time, ICC = 0.791, SEM = 494.83 ms) and global spatial metrics (draw_radius_mean, ICC = 0.767, SEM = 36.52; draw_eccentricity, ICC = 0.666, SEM = 0.09) suggested relatively high test–retest reliability. Conversely, initial tapping trial errors (tapping_err_1, ICC = 0.089; tapping_err_2, ICC = 0.061) exhibited lower stability, which could potentially be attributed to initial intra-session adaptation or daily warm-up dynamics. Meanwhile, subsequent performance (tapping_err_3, ICC = 0.651) and overall average error (tapping_mean_error, ICC = 0.556) appeared to maintain moderate-to-good consistency across stable states.

### 3.4. Feature Selection and Collinearity Mitigation

The candidate dataset comprised 423 daily features. After performing repeated measures correlation analysis using stable phase baseline data, a collinearity threshold of |rmcorr|≥0.75 was applied. The conflicts were resolved by selecting the feature with the highest ROC-AUC, taking into account the three available values in each case. This resulted in the systematic elimination of 178 collinear features, consisting predominantly of redundant cross-sectional metrics and highly coupled adjacent temporal lags.

This selection procedure resulted in a reduced-collinearity feature set of 245 clean markers, which advanced to downstream univariate inferential testing. This final feature space was heavily dominated by acoustic (112 cough and 50 speech metrics) parameters. The remaining channels were distributed by domain as follows: environmental (22), motor (17), sleep (7), respiratory (5), actigraphy (12), cardiovascular (5), skin temperature (1), and symptoms (14) (Supplementary Material Table S2). The correlation matrix is provided in Supplementary Material S3.

### 3.5. Univariate Assessment of Prodromal Phase Shifts

To characterise biomarker trajectories during the transition from a stable baseline to the prodromal phase, non-parametric paired Wilcoxon signed-rank tests were performed in the 245 candidate features in N=15 available patient pairs who presented valid baseline and prodromal monitoring windows (a patient without exacerbation events was excluded from paired analyses). The CLES index was calculated to evaluate the discriminative power of each variable. Overall, 38 biomarkers exhibited statistically significant shifts (p<0.05). Following domain-specific Benjamini–Hochberg FDR corrections (q<0.10), 19 high-confidence biomarkers across five distinct monitoring domains strictly retained significance, with 13 features maintaining robust significance at q<0.05. Table 4 details the baseline, prodromal, and delta metrics for these top-performing variables, while the complete analysis for all 245 features is provided in Supplementary Material Table S4.

Patient-reported symptoms showed robust and statistically significant deteriorations across all core respiratory indicators. Short- and long-term rolling metrics for cough frequency (q3_cough_roll_mean_3d, q3_cough_roll_std_7d, q3_cough_delta), sputum volume and purulence (q5_sputum_volume_roll_mean_3d, q5_sputum_volume_roll_std_7d, q5_sputum_volume_delta, q6_sputum_color), and dyspnea (q2_dyspnea_roll_mean_3d) demonstrated consistent upward shifts prior to clinical detection. Crucially, overall perceived general health status (q1_general_health) also exhibited a significant worsening, validating patient awareness of acute health changes during the early prodromal window.

Regarding cardiovascular tone, the prodromal transition was marked by autonomic dysregulation toward sympathetic predominance. This was evidenced by an elevated resting heart rate compared to baseline (resting_hr, resting_hr_delta), increased 7-day heart rate volatility (resting_hr_roll_std_7d), and a reduction in parasympathetic vagal tone (hrv_daily_rmssd).

While passive actigraphy, sleep, fine motor, and acoustic cough parameters remained apparently stable, vocal dynamics were characterized by reduced higher-order cepstral coefficients (speech_mfcc_8_mean, speech_mfcc_6_mean) and elevated intensity volatility (speech_intensity_var). Skin temperature was also found to be slightly higher in prodromal stages (relative_skin_temp).

Finally, ambient sensors captured subtle shifts in the indoor microclimate prior to exacerbation onset, evidenced by reduced nighttime indoor temperatures (temp_max_night) and lower relative humidity variance (hum_std_night).

### 3.6. Linear Mixed-Effects Models

LMM served as the primary analytical framework to account for within-subject correlations and multivariable adjustments, while non-parametric paired Wilcoxon tests were used as secondary exploratory screening analyses.

LMMs were implemented to evaluate longitudinal biomarker dynamics during the prodromal phase compared to the stable baseline state, controlling for subject-level clustering ($\sigma_{\mathrm{intercept}}^{2}$) and random slopes ($\sigma_{\mathrm{slope}}^{2}$) where supported. To avoid removing baseline variance between subjects, continuous features were standardised on a global scale across the entire cohort. Of the 245 candidate markers evaluated, the LMM framework identified 23 features that showed statistically significant or exploratory shifts ($q_{\mathrm{dom}}<0.10$) during the 7-day prodromal window (Table 5). The complete analysis for all 245 features is provided in Supplementary Material Table S5.

Patient-reported symptoms exhibited the largest effect sizes in the cohort during the prodromal state. Marked increases in rolling variability of 7 days were observed for sputum volume (β=0.635), cough intensity (β=0.563) and use of rescue inhalers (β=0.313). Sustained prodromal elevations were also detected in the general perceived health status (β=0.390), the 3-day rolling mean of cough (β=0.344), the 3-day rolling mean of sputum volume (β=0.249), the sputum purulence (β=0.281), and the 3-day rolling mean of dyspnea (β=0.271). High ICC values across static symptom dimensions confirmed that the baseline clustering at the patient-level represented the majority of the total variance.

Continuous physiological monitoring identified an autonomic cardiac stress prior to the onset of exacerbation, marked by increased 7-day resting heart rate volatility (β=0.477), baseline resting heart rate elevation (β=0.140), and daily resting heart rate acceleration (β=0.158).

Although daily HRV demonstrated significant changes in univariate screening (Table 4), it did not retain statistical significance as an independent predictor in the fully adjusted multivariable LMM and was consequently excluded from Table 5 ($q_{\mathrm{dom}}\geq 0.10$). Therefore, passive RMSSD trajectories before an exacerbation should be interpreted with caution as an indicator of potential multi-system autonomic stress rather than a standalone factor. Additionally, the relative skin temperature showed an exploratory increase (β=0.149), consistent with subtle thermal dysregulation.

Passive acoustic monitoring captured structural speech decay and altered cough biomechanics. Speech dynamics were characterised by significant reductions in cepstral acoustic coefficients (speech _mfcc _8 _mean, β=−0.272; speech _mfcc _6 _mean, β=−0.196) and by the increased volatility of vocal intensity (β=0.123). Cough acoustics revealed increased instability, marked by spectral envelope dispersion (cough_mfcc_9_mean_std, β=0.336; cough_mfcc_8_mean_std, β=0.313; cough_mfcc_5_mean_std, β=0.261) and elevated peak perturbation in cough vocal periodicity (cough_jitter_max, β=0.232).

Finally, environmental sensors captured microclimatic alterations during the prodromal phase, characterized by reductions in maximum nighttime indoor temperature (β=−0.423) and in 24-h relative humidity variability (β=−0.202).

#### Seasonal Confounding

To evaluate whether the observed prodromal signal trajectories were confounded by macro-environmental seasonal shifts, LMMs were re-estimated incorporating season fixed effects (Supplementary Table S6). Overall, the inclusion of seasonal controls confirmed that the core clinical and physiological biomarkers retain robust predictive significance, while environmental metrics were predominantly governed by ambient seasonal confounders.

Indoor environmental signals exhibited a substantial attenuation after controlling for season. Nighttime maximum indoor temperature experienced a sharp reduction in effect size from β=−0.423 ($q_{\mathrm{dom}}=0.022$) in the unadjusted model to β=−0.094 ($q_{\mathrm{dom}}=0.384$) in the season-adjusted model. Likewise, the 24-h relative humidity variability shifted from β=−0.202 ($q_{\mathrm{dom}}=0.013$) to β=−0.117 ($q_{\mathrm{dom}}=0.384$), completely losing domain and global statistical significance.

In contrast, symptom-based and autonomic cardiovascular stress features demonstrated high stability across both models, maintaining statistical significance. Relative skin temperature exhibited a consistent exploratory upward trend. Speech acoustic dynamics remained robust to seasonal controls, characterized by sustained reductions in cepstral coefficients and elevated vocal intensity volatility. In cough acoustics, spectral envelope dispersion metrics retained statistical significance, whereas maximum cough jitter and the cepstral MFCC-8 coefficient (mfcc_8_mean_std) attenuated slightly above the domain exploratory threshold after seasonal adjustment.

### 3.7. Univariate ROC Analysis

Predictive AUC analysis revealed a temporal divergence across feature domains, contrasting slow environmental metrics against active physiological symptoms and acoustic speech/cough features. The main results are detailed in Table 6, showing only variables that reached an AUC ≥0.60 in at least one temporal window.

Patient-reported symptom dynamics exhibited optimal predictive performance under unaggregated (raw) or short-term temporal windows (${\mathrm{EMA}}_{3}$), losing fidelity when over-smoothed. Sputum volume variability and sputum purulence achieved peak discrimination in their raw formulations, displaying performance degradation under longer temporal smoothing. Cough frequency peaked under short-term integration but dropped under extended smoothing. This can reflect the immediate, transient nature of symptom surges prior to an exacerbation.

Unsmoothed acoustic features initially performed below a discrimination threshold of 0.60. However, temporal aggregation consistently elevated all acoustic markers. Speech intensity variability and the maximum Δ MFCC-12 of cough reached their peak predictive accuracy under ${\mathrm{EMA}}_{7}$, while cough spectral variability of MFCC-9 peaked under ${\mathrm{EMA}}_{3}$. This can indicate that acoustic signals contain instantaneous noise that requires short-to-medium smoothing to resolve stable predictive patterns.

Environmental metrics showed monotonic performance gains as aggregation windows expand. Daily minimum ${\mathrm{CO}}_{2}$ levels (ppm) exhibited the largest overall improvement, rising to the highest overall predictive value in the dataset under ${\mathrm{EMA}}_{7}$ (ROC-AUC=0.646). Similarly, nighttime maximum temperature peaked under long-term smoothing. Environmental degradation might function as a slow, cumulative macro-driver requiring broader time horizons to capture risk.

No single temporal window optimized all domains simultaneously. Acute symptoms favored raw or short-term (${\mathrm{EMA}}_{3}$) windows; acoustic features optimized across ${\mathrm{EMA}}_{3}$–${\mathrm{EMA}}_{7}$; and environmental indicators strictly required long-term (${\mathrm{EMA}}_{7}$) integration. A detailed breakdown of the predictive performance distributions across all 245 digital features, cumulative proportions, and domain-stratified comparisons under different smoothing formulations (Raw, ${\mathrm{EMA}}_{3}$, and ${\mathrm{EMA}}_{7}$) is provided in Supplementary Figure S7.

### 3.8. Sensitivity Analysis

To test the stability of the proposed pipeline, we conducted a sensitivity analysis restricting ground-truth labels exclusively to event-based exacerbations (N=33). Table 7 shows the distribution of events per patient and the final number of prodromal windows used in this sensitivity study. Two episodes overlapped with associated events, and after consolidation, a total 31 prodromal windows were available for statistical analysis. The number of eligible prodromal windows ranged from 0 to 6.

First, feature selection yielded an overall overlap of 85.7% (210/245 features; Jaccard similarity = 75.0%) compared to the primary criteria (Supplementary Table S8A). Discrepancies were primarily confined to minor functional aggregations (e.g., 3-day rolling averages versus lag terms) rather than structural shifts in candidate features. These results confirm that the pre-filtered feature space was robust and unbiased by the inclusion of symptom-based exacerbation definitions.

In the Wilcoxon analysis, the comparison of the main analysis (combined symptom- and event-based exacerbations) against the sensitivity analysis (strictly restricted to event-based exacerbations) evidenced that the prodromal window retained a highly consistent physiological and symptomatic core (Supplementary Table S8B). The increase in resting heart rate (Δ=+1.824bpm, q=0.006) and the deterioration in overall health status (CLES=0.691, q=0.017) remain robust predictors of the antecedent risk period. Both sputum volume and purulence showed significant changes toward the prodromal period (q<0.05 in both analyses). Restricting the analysis to clinically validated events revealed physiological nuances consistent with greater clinical severity or the need for intervention. Specifically, it highlighted markers of greater physiological compromise that showed exploratory trends not reaching strict statistical significance in the combined analysis, notably a decrease in nocturnal oxygen saturation (mean ${\mathrm{SpO}}_{2}=-0.834\%$, q=0.067). This desaturation reflected a more severe respiratory compromise, characteristic of episodes prompting medical attention.

The frequency of use and instability of rescue inhaler usage also reached statistical significance (q=0.017 and q=0.032, respectively), aligning with clinical event definitions marked by pharmacological adjustment. Furthermore, whereas the primary analysis captured patterns in speech breathing, the event-based sensitivity analysis highlighted variations in cough acoustics, which might suggest mechanical/structural changes in the upper airways in response to acute events. Together, these results demonstrate that while the operational definition introduces domain-specific nuances, it preserved the fundamental direction and stability of the core prodromal profile.

To assess whether the deseasonalized findings depended on the clinical definition of exacerbation, the results of the primary model adjusted for season (combined event-and-symptom criterion; Table 5) were compared with results of the sensitivity analysis restricted to event-based episodes (Supplementary Table S8C). The results demonstrated a high degree of qualitative and structural agreement between both approaches. All key physiological and behavioral domains identified in the primary model (patient-reported symptoms, resting heart rate, and acoustic parameters of voice and cough) maintained the same direction of effect (sign of β) and retained statistical significance after false discovery rate correction (q-dom<0.05). The fundamental difference between the two criteria was limited to a systematic and proportional increase in the magnitude of the standardized coefficients (β) in the events-only model.

For example, general health status (q1_general_health) increased from β=0.390 to β=0.497, and the 3-day moving average of dyspnea (q2_dyspnea_roll_mean_3d) increased from β=0.271 to β=0.412. Similarly, resting heart rate rose from β=0.140 to β=0.166. Far from suggesting estimator instability, this linear amplification reflected a coherent gradient of physiological severity: event-based exacerbations represent clinically more severe episodes, translating into a larger prodromal deviation from the patient’s baseline. Similarly, both criteria were fully agreed in excluding environmental daily humidity and nighttime temperature (q-dom>0.25), confirming that the removal of seasonal biases is consistent regardless of the operational criterion used.

Finally, univariate classification performance across all feature domains demonstrated consistent predictive power when restricting the outcome to event-based exacerbations (Supplementary Table S8D). Overlapping 95% confidence intervals for shared features across definitions confirmed that the identified predictive signals were resilient to variation in diagnostic criteria. A higher proportion of individual features achieved an ROC-AUC>0.60 in the event-only analysis compared to the combined model. This shift was clinically coherent: restricting the endpoint to healthcare-seeking events isolated episodes with higher acute severity and clearer clinical boundaries, effectively enhancing the signal-to-noise ratio of individual predictors. Patient-reported symptoms retained high discriminative capacity for severe events (e.g., q1_general_health with raw ROC-AUC=0.671 and q3_cough with AUC $EMA_{3}$=0.674).

Similarly, acoustic markers, particularly speech intensity dynamics (speech_mean_intensity, AUC=0.648) and cough spectral features (cough_mfcc_1_std_mean, AUC=0.638), as well as environmental trends (${\mathrm{CO}}_{2}$ with $\mathrm{AUC}\;{\mathrm{EMA}}_{7}=0.637$), demonstrated equal or slightly superior performance under the event-based criterion, supporting the hypothesis that multimodal degradation is particularly pronounced during acute clinical events.

## 4. Discussion

### 4.1. Patient Adherence

Although telemonitoring frameworks are crucial for chronic disease self-management, their clinical efficacy remains fundamentally bottlenecked by patient compliance. In this exploratory study, we evaluated user engagement over a long-term temporal horizon, finding a hierarchical structure of adherence governed by task complexity, behavioural friction, and physical effort.

Passive data collection achieved the highest overall consistency, led by actigraphy (83.9%), nocturnal respiratory and sleep monitoring (74.5%), and indoor air quality sensing (72.2%). These high retention rates confirm that passive, ubiquitous technologies have the potential to bypass the cognitive and behavioral friction typical of long-term telehealth protocols [25]. In contrast, active monitoring compliance dropped to an average of ∼25–27% for weight tracking and peak flow measurements, while voice recordings and symptom questionnaires achieved intermediate compliance rates (∼47–51%). In particular, compliance with PEF measurements could be constrained by physical exertion (i.e., forced expiratory maneuvers during clinical degradation) as well as by technological barriers, such as Bluetooth Low Energy (BLE) pairing friction and sensor connection constraints.

These preliminary findings revealed that the frequency of exacerbation was positively correlated with adherence in both active and passive modalities. Patients with a high exacerbation burden (≥3 events) exhibited markedly higher compliance compared to low-frequency exacerbators (≤2 events), most prominently in indoor air quality monitoring (87.7% vs. 56.8%; ρ=0.61), daily peak flow measurement (39.3% vs. 14.5%; ρ=0.55), and daily symptom reporting (63.4% vs. 38.9%; ρ=0.40). This shift points to a pattern of reactive compliance or disease-driven hypervigilance. Participants who faced frequent exacerbations perceived a higher clinical threat, improving their motivation to complete monitoring tasks despite the inherent physical and technical friction. Consequently, while passive features remained essential to establish seamless baseline monitoring, active tools were actively leveraged and prioritised by the most clinically fragile patients during periods of instability. These findings should be validated in a larger study population.

#### Reliability of Fine Motor Tests

Regarding fine motor metrics evaluated during stable periods, our baseline reliability analysis showed that the time to complete the tapping task (tapping_time, ICC = 0.791) and the spatial dimensions in the drawing test (draw_radius_mean, ICC = 0.767) provided stable baseline measurements, whereas initial tapping errors exhibited higher within-subject variability. Selecting motor metrics with high test–retest reliability appears to be essential to ensure that daily measurement noise is minimized when evaluating markers in telehealth protocols.

### 4.2. Integrative Analysis of the COPD Prodrome: Digital Footprint

The preliminary findings suggest that the prodrome of an AECOPD is not an isolated respiratory event, but rather a systemic multi-organ phenomenon. The LMM and univariate analyses revealed a temporal synchronization across the patient’s subjective symptoms, phonatory mechanics, autonomic cardiovascular response, and indoor microclimate. This multi-systemic cascade could map the biological trajectory of clinical worsening:

- Airway obstruction and secretions: in the days preceding an exacerbation, the noted rises in 3-day rolling cough frequency (q3_cough_roll_mean_3d, β=0.344), sputum volume (q5_sputum_volume_roll_mean_3d, β=0.249), and sputum purulence (q6_sputum_color, β=0.281) suggested worsening bronchial inflammation and a build-up of airway secretions [46,54,55]. These changes were mirrored by elevated short-term symptom volatility (q3_cough_roll_std_7d, β=0.563; q5_sputum_volume_roll_std_7d, β=0.635).
- Thermoregulatory nocturnal dynamics: relative skin temperature exhibited an upward exploratory trend during the prodromal phase (relative_skin_temp, β=0.149).
- Autonomic dysregulation: cardiovascular monitoring showed heightened sympathetic drive and autonomic stress before exacerbation onset [56]. This was evidenced by elevated resting heart rate (resting_hr, β=0.140), acute daily acceleration (resting_hr_delta, β=0.158), and increased 7-day volatility (resting_hr_roll_std_7d, β=0.477). Daily HRV (hrv_daily_rmssd) showed a univariate reduction, but its significance attenuated in fully adjusted multivariable modeling.
- Psychomotor stability and fine motor preservation: although digitized fine motor tasks demonstrated good test–retest reliability during stable baseline periods (e.g., tapping_time ICC = 0.791), motor features did not exhibit statistically significant shifts in multivariable LMM analysis during the prodromal phase ($q_{\mathrm{dom}}\geq 0.10$). This suggests that fine psychomotor coordination was relatively preserved or masked by daily intra-subject variance during the early prodromal window.
- Behavioral-environmental feedback coupling and seasonal confounding: while indoor microclimate metrics initially exhibited significant univariate separation, incorporating season fixed effects in multivariable LMMs led to a marked attenuation of indoor environmental signals, most notably nighttime maximum temperature (temp_max_night: β=−0.094) and relative humidity daily variability (hum_std_24h: β=−0.117). This loss of statistical significance confirmed that indoor microclimate shifts were predominantly driven by macro-seasonal ambient variations rather than true disease-specific prodromal changes. In contrast, patient-reported symptoms, vocal dynamics, and resting cardiovascular parameters remained robust and seasonally invariant.

### 4.3. High-Sensitivity Features for Early Detection

A major contribution of this study was evaluating speech breathing and voluntary cough as non-invasive, low-burden proxies for respiratory function. In cough acoustics, the variations found in the dispersion of cepstral coefficients (e.g., MFCC-9) might reflect altered geometry of the vocal tract and sound-wave filtering, which might be caused by airway edema and mucus hypersecretion. Respiratory patterns and lung volume utilisation during speech have been shown to differ from those observed during resting respiration and to vary between different forms of lung disease [57]. More concretely, speech in patients with COPD during stable periods has been found to differ from speech during exacerbation [58]. In this study, decrease detected in higher-order speech cepstral coefficients (e.g., MFCC-6, MFCC-8) alongside with high voice-intensity volatility might indicate vocal muscle fatigue and pneumophonic incoordination. Loss of aerodynamic control could destabilise phonation and fragment speech, which algorithms could detect before dyspnea becomes clinically evident.

Furthermore, the multi-temporal ROC-AUC analysis (Table 6) revealed distinct domain-specific temporal dynamics. While environmental metrics acted as slow macro-trends (e.g., daily minimum ${\mathrm{CO}}_{2}$ levels optimizing under 7-day smoothing, AUC ${\mathrm{EMA}}_{7}$ = 0.646), patient-reported symptoms function performed as acute kinetic indicators concentrated within the final 72 h, and acoustic features benefited from short-to-medium temporal integration (ROC-AUC ${\mathrm{EMA}}_{3}$–AUC ${\mathrm{EMA}}_{7}$, e.g., cough_mfcc_9_mean_std AUC ${\mathrm{EMA}}_{3}$ = 0.612; speech_intensity_var AUC ${\mathrm{EMA}}_{7}$ = 0.604).

These exploratory findings suggest that an effective digital telemonitoring framework should process multi-scale temporal windows, optimizing feature extraction independently for each data domain. Consequently, multivariate machine learning models are proving necessary in this setting, as they have the potential to capture non-linear cross-domain hidden interactions.

### 4.4. Clinical Implications and the Transition Toward Passive Monitoring

Our preliminary observations in this exploratory cohort (N=16) highlight a notable difference in adherence between active and passive monitoring approaches. Intensive daily active tasks, such as home peak flow monitoring, appeared to impose a behavioral burden, resulting in overall active compliance below 30%. Interestingly, patients with higher exacerbation burdens maintained better compliance, hinting that clinical instability might drive disease-focused engagement. However, given the small sample size, these findings should be interpreted with caution. Relying heavily on demanding active reporting during stable periods may limit the continuous baseline data required by predictive models. A multi-tiered architecture—combining continuous passive tracking (e.g., nocturnal wearables and ambient air quality sensors) with low-friction active tasks (such as brief symptom questionnaires and audio recordings)—could offer a balanced strategy, securing reliable longitudinal data while minimizing patient strain.

### 4.5. Limitations and Future Work

Several methodological limitations of this study warrant consideration. First, the small single-center cohort and male predominance (68.8%) constrain the external generalizability of our findings. Additionally, four participants dropped out prematurely, underscoring the usability and engagement challenges inherent to long-term digital health monitoring. Consequently, these results should be interpreted as preliminary pilot findings requiring validation in larger, multi-center, and demographically balanced cohorts. Second, using patient-reported core symptoms both to operationalise symptom-based exacerbations (via Anthonisen criteria) and as candidate predictive features introduces potential incorporation bias (circularity). To address this overlap, we conducted a sensitivity analysis restricting the target outcome strictly to event-based exacerbations.

This validation yielded results consistent with the primary model: the same core multimodal domains, i.e., resting heart rate, cough and speech acoustics, patient-reported wellness, and ambient environment, retained their discriminative capacity, direction of effect, and statistical significance.

Crucially, narrowing the endpoint to clinical events amplified both effect sizes and ROC-AUC metrics, reflecting a biological severity gradient and confirming that the prodromal profile was not an artifact of outcome definition. Third, a key distinction must be drawn between characterizing prodromal physiological trajectories and deploying a point-of-care clinical predictive tool. In this cohort, prodromal days accounted for approximately 11% of the total monitoring timeline. In such low-prevalence scenarios, univariate decision rules, even with moderate discrimination, risk low positive predictive values and elevated false-alert rates. Furthermore, while consumer-grade wearables ensured high compliance for continuous tracking, their sleep-architecture and respiratory-rate algorithms lack formal validation against gold-standard polysomnography.

In summary, our findings represent an exploratory characterization of multimodal prodromal patterns. Translating these signal trajectories into regularized multivariate machine-learning architectures will be essential to evaluate out-of-sample generalizability and establish the true clinical utility of automated early-warning systems.

## 5. Conclusions

This exploratory pilot study provides preliminary evidence that the transition between stable and prodromal phases of AECOPD is accompanied by a quantifiable, multi-parametric digital footprint. By integrating physiological, behavioral, and environmental data streams, our findings support the concept of exacerbations as systemic, multi-organ events that can be non-invasively tracked through remote health monitoring.

The primary takeaways of this work highlight the feasibility and potential utility of alternative markers over traditional, high-friction monitoring methods. Acoustic features derived from speech mechanics and voluntary cough, specifically voice intensity volatility, higher-order speech cepstral coefficients, and cough spectral dispersions, may serve as objective, low-burden proxies for underlying airway dynamics and respiratory limitation. In addition, elevated ambient ${\mathrm{CO}}_{2}$ trends provided preliminary contextual indications of domestic confinement prior to exacerbations. However, these environmental signals warrant cautious interpretation, as their independent explanatory power attenuated significantly after controlling for macro-seasonal confounders in linear mixed-effects modeling. Furthermore, data streams exhibited distinct temporal processing dynamics: acute physiological symptoms peaked within narrow timeframes (≤72 h); acoustic features benefited from short-to-medium temporal smoothing (${\mathrm{EMA}}_{3}$–${\mathrm{EMA}}_{7}$) to mitigate signal noise; and ambient environmental metrics followed slow, cumulative trajectories best captured over 7-day moving windows.

Finally, addressing the behavioural and physical barriers of active reporting may favour a multi-tiered architecture that grounds baseline monitoring in continuous passive sensing alongside low-friction acoustic and symptom reports. Such an architecture holds promise for securing the uninterrupted longitudinal baselines required for data-driven automated early-warning systems. Future efforts will focus on evaluating multivariate multimodal models in larger, broader cohorts to advance early risk stratification and proactive AECOPD management.

## Figures and Tables

**Table 1:** Demographic and clinical characteristics of the participants (N=16).

Variable	Data
Age (years)	67.2±6.1
Male (%)	68.8
Female (%)	31.2
BMI (kg/m^2^)	28.5±5.9
Pack-years Index	38.4±17.3
FEV_1_ (mL)	875.6±379.2
FEV_1_ (%)	36.6±17.9
FVC (mL)	1888.1±467.5
FVC (%)	61.8±20.2
FEV_1_/FVC	46.0±11.4
Dyspnea mMRC	1.9±0.9
Home oxygen therapy (%)	75.0

BMI: Body mass index; FEV_1_: Forced Expiratory Volume in 1 s; FVC: Forced Vital Capacity; mMRC: Modified Medical Research Council Dyspnea Scale.

**Table 2:** Distribution by patient of AECOPD episode subtypes, excluded events, and final number of prodromal windows for analytical modeling.

AECOPD Episodes/Patient	1	2	3	4	5	6	7	8	9	10	11	12	13	14	15	16	Total
Event-based	5	5	1	2	0	0	1	2	1	2	1	4	6	1	1	1	33
Symptoms-based	2	5	2	0	0	13	1	2	0	4	3	8	1	6	4	5	56
Excluded	4	4	2	0	0	7	0	3	0	4	1	8	2	2	3	3	43
Final Prodromal Windows	3	6	1	2	0	6	2	1	1	2	3	4	5	5	2	3	46

**Table 3:** Metrics of adherence, Spearman correlation with number of AECOPD, and adherence according to exacerbation frequency by data modality.

Domain	Mean (%)	Median [IQR] (%)	Spearman (ρ)	Low (≤2 AECOPD) (%)	High (≥3 AECOPD) (%)
Passive/Continuous Monitoring					
Actigraphy	83.9±15.0	88.7 [79.0–93.7]	0.13	78.9	88.8
Nocturnal Respiratory Dynamics, Cardiovascular, Skin Temp. and Sleep	74.5±21.5	80.8 [69.2–89.6]	<0.01	71.2	77.7
Indoor Air Quality, Environment	72.2±27.0	80.5 [68.4–90.0]	0.61	56.8	87.7
Active/Patient-Reported Tasks					
Patient-Reported Symptoms and Fine Motor	51.1±25.1	51.5 [35.8–69.3]	0.40	38.9	63.4
Acoustic—Cough	49.1±24.8	50.3 [34.0–65.9]	0.39	37.7	60.5
Acoustic—Speech	47.6±24.2	45.1 [34.6–64.8]	0.36	36.3	59.0
Peak Flow	26.9±24.1	23.2 [5.2–38.3]	0.55	14.5	39.3
Weight	25.4±26.5	15.7 [6.3–34.5]	0.12	17.4	33.4

Mean adherence is presented as mean±SD. Median adherence is presented as median[IQR]. ρ represents the Spearman rank correlation coefficient between the total number of exacerbations recorded per patient and their overall adherence percentage. Adherence by subgroups is presented for participants with low (≤2 events, n=7) and high (≥3 events, n=9) exacerbation frequency.

**Table 4:** Physiological, behavioral, and environmental biomarkers exhibiting statistically significant shifts between stable and prodromal phases, evaluated via paired Wilcoxon signed-rank tests with domain-specific Benjamini–Hochberg False Discovery Rate (FDR) corrections.

Domain/Feature	Stable	Prodromal	Delta [95% CI]	CLES [95% CI]	*p*-Value	*q*-Value
Patient-Reported Symptoms						
Cough freq. 3d mean (q3_cough_roll_mean_3d)	1.790 ± 0.914	2.109 ± 0.968	0.319 [0.166, 0.472]	0.624 [0.560, 0.742]	<0.001	0.004 **
General health status (q1_general_health)	2.395 ± 0.660	2.664 ± 0.827	0.269 [0.099, 0.439]	0.676 [0.600, 0.809]	0.001	0.007 **
Sputum volume 3-day rolling mean (q5_sputum_volume_roll_mean_3d)	1.572 ± 0.825	1.805 ± 0.853	0.233 [0.109, 0.357]	0.613 [0.573, 0.720]	0.001	0.007 **
Sputum purulence (q6_sputum_color)	0.614 ± 0.885	1.034 ± 0.862	0.419 [0.216, 0.623]	0.713 [0.616, 0.867]	0.002	0.008 **
Sputum volume 7-day rolling Std.dev. (q5_sputum_volume_roll_std_7d)	0.098 ± 0.104	0.242 ± 0.191	0.144 [0.041, 0.247]	0.698 [0.555, 0.871]	0.007	0.020 *
Delta of sputum volume (q5_sputum_volume_delta)	−0.001 ± 0.010	0.063 ± 0.082	0.064 [0.016, 0.112]	0.791 [0.587, 0.956]	0.013	0.026 *
Cough 7-day rolling Std.dev. (q3_cough_roll_std_7d)	0.147 ± 0.130	0.281 ± 0.162	0.134 [0.034, 0.233]	0.707 [0.538, 0.884]	0.015	0.026 *
Dyspnea 3-day rolling mean (q2_dyspnea_roll_mean_3d)	2.607 ± 0.834	2.827 ± 1.067	0.221 [0.002, 0.439]	0.640 [0.551, 0.782]	0.015	0.026 *
Delta of cough (q3_cough_delta)	0.000 ± 0.011	0.081 ± 0.124	0.081 [0.011, 0.152]	0.767 [0.547, 0.938]	0.030	0.047 *
Skin Temperature						
Relative skin temperature (relative_skin_temp)	−0.054 ± 0.128	0.075 ± 0.242	0.130 [−0.022, 0.281]	0.658 [0.422, 0.853]	0.095	0.095 ^†^
Cardiovascular						
Delta of resting heart rate (resting_hr_delta)	0.032 ± 0.192	0.307 ± 0.413	0.276 [0.059, 0.493]	0.751 [0.587, 0.929]	0.007	0.034 *
Resting heart rate (resting_hr)	67.004 ± 8.763	68.546 ± 9.303	1.542 [0.255, 2.830]	0.556 [0.493, 0.640]	0.018	0.045 *
Daily HRV (hrv_daily_rmssd)	31.346 ± 20.972	29.524 ± 20.809	−1.822 [−3.986, 0.342]	0.436 [0.347, 0.480]	0.030	0.050 ^†^
Resting heart rate 7-day rolling Std.dev. (resting_hr_roll_std_7d)	1.240 ± 0.254	1.590 ± 0.609	0.350 [0.017, 0.683]	0.693 [0.520, 0.853]	0.064	0.080 ^†^
Acoustic—Speech						
Mean of speech MFCC-8 (speech_mfcc_8_mean)	−12.644 ± 10.868	−15.507 ± 8.688	−2.863 [−5.122, −0.604]	0.408 [0.306, 0.459]	0.001	0.031 *
Std.dev. of speech RMS amplitude (speech_intensity_var)	0.047 ± 0.024	0.050 ± 0.025	0.004 [0.001, 0.006]	0.582 [0.541, 0.679]	0.001	0.031 *
Mean of MFCC-6 (speech_mfcc_6_mean)	18.722 ± 12.904	15.966 ± 13.795	−2.756 [−4.428, −1.083]	0.434 [0.352, 0.464]	0.004	0.067 ^†^
Indoor Air Quality, Environment						
Nighttime Std.dev. of relative humidity (hum_std_night)	1.858 ± 0.518	1.568 ± 0.503	−0.291 [−0.465, −0.116]	0.332 [0.174, 0.459]	0.004	0.074 ^†^
Nighttime maximum temperature (temp_max_night)	25.631 ± 2.521	23.790 ± 3.112	−1.841 [−3.003, −0.680]	0.332 [0.179, 0.480]	0.007	0.074 ^†^

Data are reported as mean ± standard deviation across subjects, derived from per-patient averages calculated for each clinical phase. Stable: baseline monitoring periods; Prodromal: risk windows immediately preceding a validated exacerbation; Delta: absolute difference computed as meanprodromal − meanstable, presented with its 95% confidence interval [95% CI]; CLES: common language effect size, computed as *P*(Prodromal > Stable), presented with its 95% bootstrap confidence interval [95% CI]. Values > 0.50 indicate an increase in the prodromal phase, whereas values < 0.50 indicate a decrease. Statistical significance was assessed using two-sided paired Wilcoxon signed-rank tests. *p*-value: unadjusted *p*-value; *q*-value: False Discovery Rate (FDR) adjusted *p*-value calculated within each domain using the Benjamini–Hochberg procedure. Significance levels are assigned based on *q*-values: ** *q* < 0.01, * *q* < 0.05, and † *q* < 0.10 (exploratory threshold).

**Table 5:** Linear mixed-effects model performance across validated monitoring domains. Domain- and global-specific Benjamini–Hochberg False Discovery Rate (FDR) corrections were applied. Only features with statistical significance ($q_{\mathrm{dom}}$ < 0.1) are shown.

Domain/Feature	β [95% CI]	*p*-Value	$q_{\mathrm{dom}}$-Value	$q_{\mathrm{glob}}$-Value	$\sigma_{\mathrm{intercept}}^{2}$	$\sigma_{\mathrm{slope}}^{2}$	$\sigma_{\mathrm{residual}}^{2}$	ICC
Patient-Reported Symptoms								
General health status (q1_general_health)	0.390 [0.172, 0.608]	<0.001	0.002 **	0.019 *	0.877	0.172	0.245	0.782
Dyspnea 3-day rolling mean (q2_dyspnea_roll_mean_3d)	0.271 [0.033, 0.509]	0.025	0.040 *	0.207	0.972	0.209	0.236	0.805
Delta of cough (q3_cough_delta)	0.222 [0.056, 0.388]	0.009	0.016 *	0.115	0.000	0.053	0.993	0.000
Cough 3-day rolling mean (q3_cough_roll_mean_3d)	0.344 [0.196, 0.493]	<0.001	<0.001 ***	0.001 ***	1.018	0.074	0.185	0.846
Cough 7-day rolling Std.dev. (q3_cough_roll_std_7d)	0.563 [0.218, 0.909]	0.001	0.003 **	0.037 *	0.248	0.425	0.762	0.245
Rescue inhaler 7-day rolling Std (q4_rescue_inhaler_roll_std_7d)	0.313 [−0.016, 0.641]	0.062	0.087 †	0.339	0.160	0.372	0.784	0.170
Delta of sputum volume (q5_sputum_volume_delta)	0.232 [0.095, 0.369]	0.001	0.003 **	0.027 *	0.000	0.023	0.993	0.000
Sputum volume 3-day rolling mean (q5_sputum_volume_roll_mean_3d)	0.249 [0.135, 0.364]	<0.001	<0.001 ***	0.002 **	0.860	0.040	0.172	0.833
Sputum volume 7-day rolling Std (q5_sputum_volume_roll_std_7d)	0.635 [0.240, 1.031]	0.002	0.003 **	0.037 *	0.188	0.567	0.725	0.206
Sputum purulence (q6_sputum_color)	0.281 [0.147, 0.415]	<0.001	<0.001 ***	0.002 **	0.697	0.059	0.133	0.840
Skin Temperature								
Relative skin temperature (relative_skin_temp)	0.149 [−0.021, 0.318]	0.086	0.086 †	0.385	0.021	0.052	0.979	0.021
Cardiovascular								
Resting heart rate (resting_hr)	0.140 [0.031, 0.249]	0.012	0.059 †	0.121	0.734	0.038	0.113	0.867
Delta of resting heart rate (resting_hr_delta)	0.158 [0.002, 0.315]	0.047	0.078 †	0.301	0.000	0.000	0.998	0.000
Resting heart rate 7-day rolling Std (resting_hr_roll_std_7d)	0.477 [0.053, 0.900]	0.027	0.068 †	0.209	0.107	0.636	0.869	0.109
Acoustic—Speech								
Std.dev. of speech RMS amplitude (speech_intensity_var)	0.123 [0.071, 0.174]	<0.001	<0.001 ***	0.001 ***	0.903	0.001	0.157	0.852
Mean of speech MFCC-6 (speech_mfcc_6_mean)	−0.196 [−0.323, −0.070]	0.002	0.059 †	0.045 *	0.937	0.037	0.292	0.763
Mean of speech MFCC-8 (speech_mfcc_8_mean)	−0.272 [−0.460, −0.085]	0.004	0.073 †	0.067 †	1.253	0.102	0.414	0.751
Acoustic—Cough								
Std. of means cough MFCC-5 (cough_mfcc_5_mean_std)	0.261 [0.115, 0.407]	<0.001	0.053 †	0.019 *	0.133	0.024	0.873	0.132
Std. of means cough MFCC-8 (cough_mfcc_8_mean_std)	0.313 [0.108, 0.517]	0.003	0.098 †	0.047 *	0.112	0.094	0.886	0.113
Std. of means cough MFCC-9 (cough_mfcc_9_mean_std)	0.336 [0.129, 0.544]	0.002	0.084 †	0.037 *	0.166	0.102	0.809	0.170
Maximum of cough local jitter (cough_jitter_max)	0.232 [0.076, 0.387]	0.004	0.098 †	0.057 †	0.382	0.041	0.765	0.333
Indoor Air Quality, Environmental								
Nighttime maximum temperature (temp_max_night)	−0.423 [−0.690, −0.155]	0.002	0.022 *	0.041 *	0.264	0.204	0.858	0.236
24-h Std.dev. of relative humidity (hum_std_24h)	−0.202 [−0.317, −0.087]	0.001	0.013 *	0.020 *	0.132	0.000	0.896	0.128

CI: Confidence Interval; ICC: Intraclass Correlation Coefficient; $\sigma_{\mathrm{intercept}}^{2}$: per-patient random intercept variance; $\sigma_{\mathrm{slope}}^{2}$: random slope variance for prodromal status; $\sigma_{\mathrm{residual}}^{2}$: residual variance. All biomarkers were globally standardized (*Z*-score) prior to LMM fitting. *p*-value: unadjusted *p*-value; $q_{\mathrm{dom}}$-value: FDR adjusted *p*-value calculated within each domain using the Benjamini–Hochberg procedure. $q_{\mathrm{glob}}$-value: FDR adjusted *p*-value calculated using all 245 features and the Benjamini–Hochberg procedure. Significance levels are assigned based on *q*-values: *** q<0.001, ** q<0.01, * q<0.05, and †q<0.10 (exploratory threshold).

**Table 6:** Cross-sectional vs. multi-temporal predictive performance (ROC-AUC) across selected representative features.

Domain/Feature	Raw AUC [95% CI]	${\mathrm{EMA}}_{3}$ AUC [95% CI]	${\mathrm{EMA}}_{7}$ AUC [95% CI]
Patient-Reported Symptoms			
Cough frequency 3-day rolling mean (q3_cough_roll_mean_3d)	0.632 [0.600, 0.665]	0.634 [0.602, 0.667]	0.615 [0.582, 0.648]
Sputum volume 7-day rolling std. (q5_sputum_volume_roll_std_7d)	0.625 [0.589, 0.662]	0.615 [0.579, 0.652]	0.624 [0.589, 0.659]
Sputum purulence (q6_sputum_color)	0.600 [0.565, 0.636]	0.597 [0.563, 0.631]	0.577 [0.543, 0.611]
Acoustic—Speech			
Std.dev. of speech RMS amplitude (speech_intensity_var)	0.586 [0.555, 0.617]	0.602 [0.571, 0.634]	0.604 [0.571, 0.636]
Acoustic—Cough			
Std.dev. of means of MFCC-9 (cough_mfcc_9_mean_std)	0.595 [0.563, 0.628]	0.612 [0.581, 0.644]	0.610 [0.579, 0.641]
Maximum of means of (cough) Δ MFCC-12 (cough_delta_mfcc_12_mean_max)	0.585 [0.553, 0.617]	0.601 [0.570, 0.632]	0.606 [0.574, 0.637]
Indoor Air Quality, Environment			
Daily minimum ${\mathrm{CO}}_{2}$ level in ppm (co2_min_24h)	0.581 [0.550, 0.613]	0.614 [0.583, 0.645]	0.646 [0.616, 0.676]
Nighttime maximum ambient temperature (temp_max_night)	0.616 [0.588, 0.645]	0.621 [0.593, 0.649]	0.625 [0.597, 0.653]

AUC values represent individual univariate discriminative capacities computed independently for each biomarker across all longitudinal observations, without prior multivariate feature selection.

**Table 7:** Sensitivity analysis: distribution per patient of event-based AECOPD episodes, excluded, and identified prodromal windows for analytical modeling.

AECOPD Episodes/Patient	1	2	3	4	5	6	7	8	9	10	11	12	13	14	15	16	Total
Event-based	5	4	1	2	0	0	1	2	1	2	1	4	6	2	1	1	33
Excluded	1	0	0	0	0	0	0	0	0	0	0	1	0	0	0	0	2
Final Prodromal Windows	4	4	1	2	0	0	1	2	1	2	1	3	6	2	1	1	31

## Data Availability

The data are not publicly available due to privacy or ethical restrictions. The data that support the findings of this study are available on request from the corresponding author for researchers who meet the criteria for confidential data access as stipulated by participant-informed consent and the Institutional Research Ethics Committee of the province of Cadiz, Spain.

## Supplementary Materials

The following supporting information can be downloaded at: https://www.mdpi.com/article/10.3390/bioengineering13101195/s1: Supplementary Material S1: Multimodal feature extraction; Supplementary Material S2: Comprehensive breakdown of the 245 curated and independent features selected through repeated measures correlation and AUC filtering, categorized by domain; Supplementary Material S3: Correlation matrix; Supplementary Material S4: Physiological, behavioral, and environmental features, evaluated via paired Wilcoxon signed-rank tests with domain- and global-specific Benjamini–Hochberg False Discovery Rate (FDR) corrections; Supplementary Material S5: Physiological, behavioral, and environmental features, evaluated via Linear Mixed Models (LMM) with domain- and global-specific Benjamini–Hochberg False Discovery Rate (FDR) corrections; Supplementary Material S6: Comparison of LMMs before and after adjustment for season fixed effects, reporting standardized β coefficients, 95% CIs, raw *p*-values, and domain- and global-specific false discovery rate (FDR) *q*-values across all features; Supplementary Material S7: Distribution of Area Under the ROC Curve (ROC-AUC) across all markers and temporal smoothing windows; Supplementary Material S8: Sensitivity Analysis: feature selection stability after the correlation analysis between primary (symptom-based and event-based) and sensitivity (only event-based) exacerbation criteria across all explored domains.

## Abbreviations

The following abbreviations are used in this manuscript:

AECOPD	Acute Exacerbation of Chronic Obstructive Pulmonary Disease
AI	Artificial Intelligence
AUC	Area Under the ROC Curve
BLE	Bluetooth Low Energy
BMI	Body Mass Index
CAT	COPD Assessment Test
CI	Confidence Interval
CLES	Common Language Effect Size
CO_2_	Carbon Dioxide
COPD	Chronic Obstructive Pulmonary Disease
EHR	Electronic Health Record
EMA	Exponential Moving Average
FEV_1_	Forced Expiratory Volume in 1 s
FOT	Forced Oscillation Technique
FVC	Forced Vital Capacity
GOLD	Global Initiative for Chronic Obstructive Lung Disease
HRV	Heart Rate Variability
IAQ	Indoor Air Quality
IDE	Integrated Development Environment
IoT	Internet of Things
IQR	Interquartile range
LMM	Linear Mixed-Effects Model
NB-IoT	Narrowband Internet of Things
PM	Particulate Matter
RMSSD	Root Mean Square of Successive Differences
SpO_2_	Peripheral Oxygen Saturation
SUS	System Usability Scale
UCD	User-Centred Design
VOC	Volatile Organic Compounds
WSGI	Web Server Gateway Interface
YAML	YAML Ain’t Markup Language
