# Supplementary material for: Multimodal Prodromal Patterns Associated with COPD Exacerbations in Home Telemonitoring: An Exploratory Observational Study
Source: Bioengineering (Basel). 2026 Oct 14;13(10):1195. doi: 10.3390/bioengineering13101195 (PMC13701270; doi:10.3390/bioengineering13101195)
Supplement: Supplementary file 1 [file bioengineering-13-01195-s001.zip › Supplementary Material S1.pdf]

Supplementary Material S1

Multimodal Feature Extraction

The heterogeneous raw data streams acquired from the telemonitoring ecosystem were processed to generate a highly granular, daily feature space of 427 features per patient, categorized into distinct clinical and environmental domains.

Indoor Air Quality & Environmental Metrics

Continuous indoor air quality readings were aggregated into 24-hour daily profiles, as well as segmented into diurnal and nocturnal cycles to capture circadian variations in environmental exposure. For each pollutant and climatic variable, specifically particulate matter (PM_1, $PM_{2.5}$, PM_4, PM_{10}), CO_2, volatile organic compounds (VOCs), ambient temperature, and relative humidity, four descriptive statistics were calculated: mean, standard deviation, maximum, and minimum values. This resulted in 96 daily environmental features designed to capture both sustained exposure levels and acute environmental spikes.

Wearable-Derived Physiology, Sleep, and Activity

Continuous data from the wearable activity tracker yielded 24 daily features reflecting the patient's cardiorespiratory and behavioral baseline:

- Actigraphy (5 features): physical activity was quantified using daily step counts and the time spent across four intensity tiers (sedentary, lightly active, fairly active, and very active minutes).
- Autonomic and cardiovascular tone (3 features): measured using features that served as a proxy for systemic stress and autonomic nervous system balance, including resting heart rate (RHR) and Heart Rate Variability (HRV) estimated via the root mean square of successive differences (RMSSD) using both full-day data and data restricted to deep sleep.
- Nocturnal respiratory dynamics (7 features): continuous peripheral oxygen saturation (SpO_2) was summarized using 3 statistics (mean, minimum, and maximum). In addition, the average nocturnal breathing rate and the segregated breathing rate by sleep stages were estimated to identify subtle hypoxemic events or tachypnea during sleep.
- Sleep architecture (8 features): sleep quality was parameterized using time in bed, wake time, total minutes asleep, sleep efficiency, awakening counts, and time spent in specific sleep stages (light, deep, and REM).
- Skin temperature (1 feature): nocturnal relative skin temperature variation ($\text{tempSkin}_{\text{relative}}$) was tracked, serving as an early indicator of febrile shifts often preceding clinical decompensation.

Patient Reported Symptoms and Pulmonary Function

Patient-reported outcome measures were digitized into 6 features using a Likert-scale symptom diary, quantifying general health perception, dyspnea, cough severity, reliance on rescue inhalers, and sputum characteristics (volume and purulence). Concurrently, respiratory function was represented using a peak flow sensor during three consecutive peak expiratory flow (PEF) maneuvers. Peak flows and their daily mean were estimated. Finally, body weight was registered to monitor fluid retention or cachexia associated with exacerbation dynamics.

Fine-Motor Assessment

To capture subtle neurological and cognitive-motor declines potentially associated with AECOPD-induced hypoxemia or systemic inflammation, 14 features were extracted from two smartphone-based gamified tasks:

- Continuous circle-tracing task (7 features) to evaluate kinematic proficiency. The patient had to draw a circle on the screen with their finger, as perfect and centered as possible. Extracted metrics included trajectory smoothness, eccentricity, bounding area, circularity index, spatial deviation, and mean and standard deviation of radius from centroid.
- Target-tapping task (7 features) to evaluate visuomotor accuracy and fatigue. The patient had three attempts to try to touch a square spot on the screen. The error margins across the three sequential attempts were recorded daily, as well as the execution time, overall mean error, maximum error (indicative of attentional micro-lapses), and intra-test standard deviation.

Acoustic Features (Speech and Cough)

Features derived from sounds from both the upper and lower respiratory tracts, as well as from the voice, have become important indicators of respiratory function. In this study, voluntary coughing and speech-related breathing were explored for their potential as early indicators of AECOPD. Acoustic signal processing provided the highest-dimensional set of features, yielding 208 features as follows:

- Voluntary cough analysis (169 features): quantitative characterization of voluntary cough acoustics holds significant clinical utility for computer-aided diagnostics and remote respiratory telemonitoring, having emerged as a non-invasive and low-cost screening and tracking tool in obstructive airway diseases such as asthma, COPD, and allergic rhinitis. In this study, we examined the microstructural acoustic properties of voluntary cough sounds to evaluate possible differences in these properties between the stable and prodromal phases in AECOPD. To standardize cough sound acquisition, participants were instructed to slightly open their mouths, inhale deeply, and perform two to three consecutive maximal-effort coughs. The resulting acoustic episodes were segmented and analyzed to extract a comprehensive set of temporal, energy, and spectral descriptors. The features included the mean, maximum, and minimum values of the cough duration, zero-crossing rate (ZCR), log-energy, spectral centroid, spectral rolloff, band energy ratio, spectral moments (kurtosis and skewness), spectral entropy, crest factor, and the first three formants (F_1–F_3). Furthermore, Mel-Frequency Cepstral Coefficients (MFCCs), dynamic spectral shifts (ΔMFCCs), harmonics-to-noise ratio (HNR), and perturbation measures (jitter and shimmer) were calculated. These features aimed to map specific acoustic signatures to underlying pathophysiology such as bronchoconstriction, airway inflammation, and mucus hypersecretion.
- Speech breathing (39 features): to non-invasively monitor changes in patient-reported lung function, a standardized voice recording task was integrated into the daily telemonitoring protocol. This paradigm leverages the speech airstream mechanism as a digital proxy for predicting and diagnosing underlying respiratory decline. Patients were instructed to read the phonetically balanced Spanish phrase, *"El sonido del mar me llena de paz y felicidad"*, in English: *"The sound of the sea fills me with peace and happiness"*. This specific phrase was selected for its strategic pneumophonetic and aerodynamic constraints. Mechanically, the absence of natural syntactic pauses may act as a stress-test for expiratory control. This allows acoustic algorithms to potentially detect micro-inspiratory pauses or premature volume decay secondary to air trapping and reduced forced expiratory volume in 1 second (FEV_1) during prodromal exacerbation phases. Furthermore, the high density of unvoiced alveolar fricatives (/s/) demands sustained, turbulent airflow that can be sensitive to respiratory muscle fatigue and bronchoconstriction. Concurrently, stable open vowels (e.g., in *"mar"* and *"paz"*) can facilitate the computational extraction of vocal perturbation metrics, specifically jitter and shimmer. Consequently, longitudinal changes in fundamental frequency (F_0), harmonic-to-noise ratio (HNR), and speech rate markers could serve as digital indicators of underlying airway inflammation and prosodic alterations before clinical deterioration. Acoustic features extracted included total duration of speech, speech rate, mean intensity, pause dynamics (total count and duration), pitch, F_0 extrema (minimum and maximum), perturbation measures (local jitter and local shimmer), and HNR. Additionally, the first 13 Mel-frequency cepstral coefficients (MFCCs), along with their corresponding standard deviations, were computed.

Temporal Feature Engineering

Given the gradual physiological decline and prodromal variations that characterise AECOPD, recording the longitudinal evolution of a patient's condition over time can be clinically more meaningful than assessing single cross-sectional snapshots from day-to-day. Consequently, we implemented an automated temporal feature engineering pipeline based on a time-series vectorisation approach. A subset of 15 physiological and behavioural source variables, spanning all monitoring domains, was selected for temporal transformation. For each variable x on day t, the original static feature space was expanded into a dynamic multi-interval trajectory using three distinct mathematical operators:

1. 1-day and 2-day lags (short-term clinical inertia): to provide immediate historical context and capture short-term physiological dependency, the values of the two preceding days were appended as distinct features:

$$x_{\text{lag1}} = x_{t-1}, \quad x_{\text{lag2}} = x_{t-2} \tag{S1}$$

2. Deltas (acute gradients of change) were estimated to model abrupt physiological decompensations and acute symptomatic exacerbation trajectories. A discrete first-order derivative was calculated as follows:

$$\Delta x = x_t - x_{t-1} \tag{S2}$$

3. Rolling statistics (contextual baselines and volatility): to mitigate ambient sensor noise, smooth daily stochastic fluctuations, and establish personalised homeostatic boundaries, moving window aggregations were engineered:

   - 3-day rolling mean ($\mu_{3\text{d}}$) modelled sustained, progressive multi-day trends:

$$\mu_{3\text{d}} = \frac{1}{3}\sum_{i=0}^{2} x_{t-i} \tag{S3}$$

   - 7-Day rolling standard deviation ($\sigma_{7\text{d}}$) was used as a digital proxy for homeostatic instability and physiological volatility during the week preceding a potential event:

$$\sigma_{7\text{d}} = \sqrt{\frac{1}{7}\sum_{i=0}^{6}(x_{t-i} - \mu_{7\text{d}})^2} \tag{S4}$$

As detailed in Table S1, this systematic transformation expanded the selected 15 base variables into a highly dense temporal subset of 75 engineered features.

Table S1: Taxonomy of selected base variables subjected to temporal feature engineering.

**Domain**	**Base Variable**	**Clinical / Feature Interpretation**
Actigraphy & Weight	steps	Daily physical activity / Ambulation level
	sedentary time in minutes	Sedentary behavior and immobility tracking
Cardiovascular	resting heart rate	Autonomic tone and systemic stress proxy
Patient Reported Symptoms	dyspnea	Patient-reported dyspnea severity
	cough	Self-reported cough severity level
	rescue inhaler	Frequency of rescue bronchodilator use
	sputum volume	Sputum volume tracking
Fine Motor	mean error (tapping test)	Visuomotor accuracy and attentional lapses
	circularity (circle tracing task)	Kinematic smoothness (resting tremor proxy)
Acoustic - Speech	mean intensity	Mean vocal intensity and phonatory energy
	pause counts	Micro-inspiratory pause counts
	speech duration	Total expiratory sentence duration
	speech rate	Speech rate (syllables/seconds ratio)
	total pause duration	Cumulative duration of pauses during speech
	mean HNR	Harmonic-to-Noise Ratio (vocal tract stability)
