# Supplementary material for: Multimodal Prodromal Patterns Associated with COPD Exacerbations in Home Telemonitoring: An Exploratory Observational Study
Source: Bioengineering (Basel). 2026 Oct 14;13(10):1195. doi: 10.3390/bioengineering13101195 (PMC13701270; doi:10.3390/bioengineering13101195)
Supplement: Supplementary file 1 [file bioengineering-13-01195-s001.zip › Supplementary Material S2.pdf]

Supplementary Material S2

Table S2: Comprehensive breakdown of the 245 curated and independent features selected through repeated measures correlation and AUC filtering, categorized by domain.

Domain (Count)	Selected Independent Features
Patient Reported Symptoms (n=14)	q1_general_health, q6_sputum_color, q2_dyspnea_delta, q2_dyspnea_roll_mean_3d, q2_dyspnea_roll_std_7d, q3_cough_delta, q3_cough_roll_mean_3d, q3_cough_roll_std_7d, q4_rescue_inhaler_delta, q4_rescue_inhaler_roll_mean_3d, q4_rescue_inhaler_roll_std_7d, q5_sputum_volume_delta, q5_sputum_volume_roll_mean_3d, q5_sputum_volume_roll_std_7d
Actigraphy (n=12)	steps, fairly_active_mins, lightly_active_mins, very_active_mins, steps_lag1, steps_lag2, steps_delta, steps_roll_mean_3d, steps_roll_std_7d, sedentary_mins_lag2, sedentary_mins_delta, sedentary_mins_roll_std_7d
Cardiovascular (n=5)	resting_hr, hrv_daily_rmssd, hrv_deep_rmssd, resting_hr_delta, resting_hr_roll_std_7d
Nocturnal Respiratory Dynamics (n=5)	spo2_mean, avg_breathing_rate, deep_breathing_rate, rem_breathing_rate, light_breathing_rate
Skin Temperature (n=1)	relative_skin_temp
Fine Motor (n=17)	tapping_time, tapping_err1, tapping_err2, tapping_err3, tapping_err_mean_delta, tapping_err_mean_lag1, tapping_err_mean_lag2, tapping_err_mean_roll_std_7d, draw_circularity_delta, draw_circularity_roll_std_7d draw_circularity_lag1, draw_circularity_lag2, draw_spatial_deviation, draw_eccentricity, draw_smoothness, draw_radius_mean, draw_radius_std
Sleep Architecture (n=7)	time_in_bed, wake_mins, total_asleep_mins, sleep_efficiency, awakenings_count, deep_sleep_mins, rem_sleep_mins
Acoustic - Cough (n=112)	cough_dur_std, cough_dur_max, cough_zcr_mean_std, cough_log_energy_mean_std, cough_crest_factor_std, cough_crest_factor_max, cough_spec_centroid_mean_mean, cough_spec_centroid_std_mean, cough_spec_centroid_std_std, cough_spec_rolloff_mean_std, cough_spec_entropy_mean, cough_spec_entropy_std, cough_ber_std, cough_kurtosis_std, cough_skewness_std, cough_skewness_max, cough_mfcc_1_mean_mean, cough_mfcc_1_mean_std, cough_mfcc_1_std_mean, cough_mfcc_1_std_std, cough_delta_1_mean_max, cough_mfcc_2_mean_std, cough_mfcc_2_mean_max, cough_mfcc_2_std_std, cough_mfcc_2_std_max, cough_delta_2_mean_std, cough_delta_2_mean_max, cough_mfcc_3_mean_std, cough_mfcc_3_mean_max, cough_mfcc_3_std_mean, cough_mfcc_3_std_std, cough_delta_3_mean_mean, cough_delta_3_mean_std, cough_mfcc_4_mean_mean, cough_mfcc_4_mean_std, cough_mfcc_4_std_std, cough_mfcc_4_std_max, cough_delta_4_mean_mean, cough_delta_4_mean_std, cough_mfcc_5_mean_mean, cough_mfcc_5_mean_std, cough_mfcc_5_std_mean, cough_mfcc_5_std_std, cough_delta_5_mean_mean, cough_delta_5_mean_std, cough_mfcc_6_mean_mean, cough_mfcc_6_mean_std, cough_mfcc_6_std_std, cough_mfcc_6_std_max, cough_delta_6_mean_mean, cough_delta_6_mean_std, cough_mfcc_7_mean_mean, cough_mfcc_7_mean_std, cough_mfcc_7_std_mean, cough_mfcc_7_std_std, cough_delta_7_mean_mean, cough_delta_7_mean_std, cough_delta_7_mean_max, cough_mfcc_8_mean_std, cough_mfcc_8_mean_max, cough_mfcc_8_std_mean, cough_mfcc_8_std_std, cough_delta_8_mean_std, cough_delta_8_mean_max, cough_mfcc_9_mean_mean, cough_mfcc_9_mean_std, cough_mfcc_9_std_std, cough_mfcc_9_std_max, cough_delta_9_mean_std, cough_delta_9_mean_max, cough_mfcc_10_mean_mean, cough_mfcc_10_mean_std, cough_mfcc_10_std_mean,f cough_mfcc_10_std_std, cough_delta_10_mean_mean, cough_delta_10_mean_std, cough_delta_10_mean_max, cough_mfcc_11_mean_std, cough_mfcc_11_mean_max, cough_mfcc_11_std_mean, cough_mfcc_11_std_std, cough_delta_11_mean_mean, cough_delta_11_mean_std, cough_mfcc_12_mean_std, cough_mfcc_12_mean_max, cough_mfcc_12_std_mean, cough_mfcc_12_std_std, cough_delta_12_mean_std, cough_delta_12_mean_max, cough_mfcc_13_mean_std, cough_mfcc_13_mean_max, cough_mfcc_13_std_std, cough_mfcc_13_std_max, cough_delta_13_mean_std, cough_delta_13_mean_max, cough_f1_mean_std, cough_f1_mean_max, cough_f2_mean_std, cough_f2_mean_max, cough_f3_mean_std, cough_f3_mean_max, cough_f0_mean_mean, cough_f0_std_mean, cough_f0_std_max, cough_hnr_mean_std, cough_hnr_mean_max, cough_hnr_mean_mean_delta, cough_hnr_mean_mean_lag2, cough_hnr_mean_mean_roll_std_7d, cough_jitter_std, cough_jitter_max, cough_shimmer_std
Acoustic - Speech (n=50)	speech_pitch, speech_shimmer, speech_hnr, speech_pause_count, speech_total_pause_dur, speech_total_pause_dur_delta, speech_total_pause_dur_lag1, speech_total_pause_dur_lag2, speech_total_pause_dur_roll_std_7d, speech_f0_min, speech_pitch_var, speech_intensity_var, speech_rhythm_var, speech_mfcc_1_mean, speech_mfcc_1_std, speech_mfcc_2_mean, speech_mfcc_2_std, speech_mfcc_3_mean, speech_mfcc_3_std, speech_mfcc_4_mean, speech_mfcc_4_std, speech_mfcc_5_mean, speech_mfcc_5_std, speech_mfcc_6_mean, speech_mfcc_6_std, speech_mfcc_7_mean, speech_mfcc_7_std, speech_mfcc_8_mean, speech_mfcc_8_std, speech_mfcc_9_mean, speech_mfcc_9_std, speech_mfcc_10_mean, speech_mfcc_10_std, speech_mfcc_11_mean, speech_mfcc_11_std, speech_mfcc_12_mean, speech_mfcc_12_std, speech_mfcc_13_mean, speech_mfcc_13_std, speech_mean_intensity_delta, speech_mean_intensity_roll_std_7d, speech_mean_intensity_roll_mean_3d, speech_pause_count_delta, speech_pause_count_lag1, speech_pause_count_lag2, speech_pause_count_roll_mean_3d, speech_pause_count_roll_std_7d, speech_duration_delta, speech_active_duration_delta, speech_active_duration_lag2, speech_active_duration_roll_std_7d
Indoor Air Quality, Environment (n=22)	pm25_min_24h, pm10_max_24h, temp_std_24h, hum_std_24h, hum_min_24h, co2_min_24h, voc_mean_24h, voc_std_24h, voc_min_24h, pm4_min_day, pm4_min_night, pm10_max_night, temp_std_night, temp_max_night, hum_std_night, co2_mean_day, co2_std_night, voc_mean_night, voc_std_night, voc_min_day, voc_min_night, voc_max_day
