# Supplementary material for: Multimodal Prodromal Patterns Associated with COPD Exacerbations in Home Telemonitoring: An Exploratory Observational Study
Source: Bioengineering (Basel). 2026 Oct 14;13(10):1195. doi: 10.3390/bioengineering13101195 (PMC13701270; doi:10.3390/bioengineering13101195)
Supplement: Supplementary file 1 [file bioengineering-13-01195-s001.zip › Supplementary Material S4.pdf]

Supplementary Material S4

Table S4: Physiological, behavioral, and environmental features, evaluated via paired Wilcoxon signed-rank tests with domain- and global-specific Benjamini–Hochberg False Discovery Rate (FDR) corrections.

Domain / Feature	Stable	Prodromal	Delta [95% CI]	CLES	p-value	q-dom	q-glob
Patient Reported Symptoms							
q1_general_health	2.395 ± 0.660	2.664 ± 0.827	+0.269 [+0.099, +0.439]	0.676	0.001	0.007**	0.072†
q2_dyspnea_delta	0.003 ± 0.011	0.034 ± 0.052	+0.031 [+0.000, +0.062]	0.613	0.093	0.130	0.428
q2_dyspnea_roll_mean_3d	2.607 ± 0.834	2.827 ± 1.067	+0.221 [+0.002, +0.439]	0.640	0.015	0.026*	0.217
q2_dyspnea_roll_std_7d	0.178 ± 0.177	0.198 ± 0.164	+0.020 [−0.073, +0.112]	0.547	0.303	0.326	0.618
q3_cough_delta	0.000 ± 0.011	0.081 ± 0.124	+0.081 [+0.011, +0.151]	0.767	0.030	0.047*	0.256
q3_cough_roll_mean_3d	1.790 ± 0.914	2.109 ± 0.968	+0.319 [+0.166, +0.472]	0.624	0.000	0.004**	0.072†
q3_cough_roll_std_7d	0.147 ± 0.130	0.281 ± 0.162	+0.134 [+0.034, +0.233]	0.707	0.015	0.026*	0.217
q4_rescue_inhalerdelta	0.004 ± 0.008	0.027 ± 0.050	+0.024 [−0.006, +0.053]	0.622	0.123	0.157	0.442
q4_rescue_inhalerroll_mean_3d	2.308 ± 0.786	2.363 ± 0.770	+0.055 [−0.129, +0.238]	0.558	0.600	0.600	0.840
q4_rescue_inhaler_roll_std_7d	0.084 ± 0.084	0.150 ± 0.165	+0.066 [−0.009, +0.142]	0.551	0.196	0.229	0.546
q5_sputum_volume_delta	−0.001 ± 0.010	0.063 ± 0.082	+0.064 [+0.016, +0.112]	0.791	0.013	0.026*	0.214
q5_sputum_volume_roll_mean_3d	1.572 ± 0.825	1.805 ± 0.853	+0.233 [+0.109, +0.357]	0.613	0.001	0.007**	0.072†
q5_sputum_volume_roll_std_7d	0.098 ± 0.104	0.242 ± 0.191	+0.144 [+0.041, +0.247]	0.698	0.007	0.020*	0.125
q6_sputum_color	0.614 ± 0.885	1.034 ± 0.862	+0.419 [+0.216, +0.623]	0.713	0.002	0.008**	0.091†
Cardiovascular							
hrv_daily_rmssd	31.346 ± 20.972	29.524 ± 20.809	−1.822 [−3.986, +0.342]	0.436	0.030	0.050†	0.256
hrv_deep_rmssd	35.539 ± 22.379	36.178 ± 28.361	+0.640 [−5.192, +6.471]	0.467	0.847	0.847	0.966
resting_hr	67.004 ± 8.763	68.546 ± 9.303	+1.542 [+0.255, +2.830]	0.556	0.018	0.045*	0.240
resting_hr_delta	0.032 ± 0.192	0.307 ± 0.413	+0.276 [+0.059, +0.493]	0.751	0.007	0.034*	0.125
resting_hr_roll_std_7d	1.240 ± 0.254	1.590 ± 0.609	+0.350 [+0.017, +0.683]	0.693	0.064	0.080†	0.360
Nocturnal Respiratory Dynamics							
avg_breathing_rate	16.906 ± 3.221	16.976 ± 3.473	+0.070 [−0.757, +0.897]	0.493	0.934	0.934	0.980
deep_breathing_rate	16.858 ± 2.903	17.488 ± 3.374	+0.630 [−0.463, +1.723]	0.547	0.679	0.848	0.875
light_breathing_rate	16.559 ± 3.659	16.024 ± 3.708	−0.535 [−1.171, +0.100]	0.467	0.229	0.573	0.585
rem_breathing_rate	14.745 ± 3.262	15.049 ± 3.174	+0.304 [−0.468, +1.077]	0.533	0.359	0.599	0.652
spo2_mean	94.199 ± 1.576	93.856 ± 1.870	−0.343 [−0.802, +0.115]	0.449	0.229	0.573	0.585
Skin Temperature							
relative_skin_temp	−0.054 ± 0.128	0.075 ± 0.242	+0.130 [−0.022, +0.281]	0.658	0.095	0.095†	0.429
Actigraphy							
fairly_active_mins	9.062 ± 7.611	8.983 ± 7.190	-0.078 [-3.700, +3.543]	0.484	0.639	1.000	0.865
lightly_active_mins	163.728 ± 58.463	167.523 ± 68.629	+3.795 [-15.856, +23.446]	0.511	0.524	1.000	0.798
sedentaryMinutes_delta	−0.544 ± 3.610	−0.078 ± 17.706	+0.466 [-10.454, +11.386]	0.489	0.561	1.000	0.821
sedentaryMinutes_lag2	1063.434 ± 163.880	1101.676 ± 172.786	+38.242 [-23.880, +100.363]	0.564	0.330	1.000	0.637
sedentaryMinutes_roll_std_7d	98.073 ± 26.055	107.072 ± 33.554	+8.999 [-12.507, +30.505]	0.556	0.890	1.000	0.979
steps	4549.736 ± 2336.971	4270.491 ± 2515.901	-279.245 [-1042.689, +484.199]	0.471	0.679	1.000	0.875
steps_delta	−37.462 ± 64.782	−60.957 ± 347.408	-23.494 [-230.210, +183.222]	0.591	0.890	1.000	0.979
steps_lag1	4577.715 ± 2334.668	4312.960 ± 2569.590	-264.754 [-974.480, +444.971]	0.480	0.934	1.000	0.980
steps_lag2	4606.145 ± 2352.547	4323.268 ± 2514.075	-282.877 [-983.166, +417.412]	0.467	0.978	1.000	1.000
steps_roll_mean_3d	4541.208 ± 2309.029	4288.350 ± 2521.380	-252.858 [-991.735, +486.018]	0.467	0.890	1.000	0.979
steps_roll_std_7d	1792.462 ± 1017.762	1758.882 ± 1014.448	-33.579 [-320.452, +253.294]	0.493	1.000	1.000	1.000
very_active_mins	8.319 ± 10.064	6.775 ± 7.544	-1.543 [-4.854, +1.767]	0.471	0.277	1.000	0.600
Fine Motor							
draw_circularity_delta	0.003 ± 0.010	−0.001 ± 0.013	−0.004 [−0.015, +0.008]	0.469	0.903	1.000	0.979
draw_circularity_lag1	0.609 ± 0.175	0.535 ± 0.254	−0.075 [−0.192, +0.043]	0.424	0.426	0.659	0.701
draw_circularity_lag2	0.604 ± 0.177	0.544 ± 0.236	−0.060 [−0.168, +0.048]	0.424	0.268	0.659	0.585
draw_circularity_roll_std_7d	0.211 ± 0.090	0.208 ± 0.090	−0.003 [−0.043, +0.037]	0.475	0.670	0.949	0.875
draw_spatial_deviation	99.334 ± 34.700	104.122 ± 35.537	+4.788 [−2.282, +11.859]	0.546	0.173	0.659	0.498
tapping_err_mean_delta	−0.079 ± 0.167	0.012 ± 0.079	+0.091 [−0.024, +0.207]	0.617	0.173	0.659	0.498
tapping_err_mean_lag1	1.568 ± 0.760	1.417 ± 0.820	−0.151 [−0.597, +0.296]	0.403	0.119	0.659	0.435
tapping_err_mean_lag2	1.640 ± 0.842	1.355 ± 0.766	−0.284 [−0.717, +0.149]	0.362	0.020	0.344	0.240
tapping_err_mean_roll_std_7d	0.299 ± 0.097	0.314 ± 0.137	+0.016 [−0.044, +0.076]	0.485	1.000	1.000	1.000
tapping_err1	0.018 ± 0.007	0.019 ± 0.009	+0.001 [−0.003, +0.004]	0.485	0.855	1.000	0.966
tapping_err2	2.224 ± 1.074	2.129 ± 0.989	−0.095 [−0.593, +0.403]	0.485	0.855	1.000	0.966
tapping_err3	2.266 ± 1.379	2.081 ± 1.593	−0.185 [−1.190, +0.819]	0.408	0.426	0.659	0.701
draw_eccentricity	0.590 ± 0.140	0.574 ± 0.157	−0.016 [−0.047, +0.014]	0.464	0.296	0.659	0.614
draw_smoothness	1.355 ± 0.199	1.458 ± 0.332	+0.104 [−0.052, +0.260]	0.571	0.391	0.659	0.670
tapping_time	1809.488 ± 1028.152	1716.963 ± 1100.895	−92.525 [−290.486, +105.437]	0.469	0.391	0.659	0.670
draw_radius_mean	286.908 ± 64.821	282.801 ± 69.197	−4.107 [−12.467, +4.254]	0.495	0.358	0.659	0.652
draw_radius_std	30.376 ± 18.518	29.726 ± 19.279	−0.651 [−3.912, +2.611]	0.475	0.952	1.000	0.980
Sleep Architecture							
awakenings_count	10.020 ± 3.177	9.141 ± 2.953	−0.878 [−2.244, +0.487]	0.409	0.303	0.840	0.618
deep_sleep_mins	51.836 ± 12.798	52.317 ± 16.998	+0.481 [−7.326, +8.288]	0.484	0.847	0.847	0.966
sleep_efficiency	63.394 ± 12.350	64.107 ± 12.424	+0.713 [−2.226, +3.652]	0.542	0.720	0.840	0.909
rem_sleep_mins	63.093 ± 16.149	60.006 ± 22.800	−3.087 [−14.300, +8.127]	0.391	0.389	0.840	0.670
time_in_bed	492.226 ± 68.095	503.582 ± 120.922	+11.355 [−30.219, +52.930]	0.551	0.524	0.840	0.798
total_asleep_mins	295.591 ± 102.655	296.717 ± 133.221	+1.126 [−27.988, +30.241]	0.484	0.679	0.840	0.875
wake_mins	58.495 ± 12.547	60.927 ± 19.830	+2.432 [−4.245, +9.109]	0.524	0.639	0.840	0.865
Acoustic Speech							
speech_duration_delta	−0.005 ± 0.016	0.008 ± 0.034	+0.012 [-0.008, +0.032]	0.674	0.217	0.787	0.585
speech_total_pause_dur	0.413 ± 0.257	0.384 ± 0.277	-0.029 [-0.146, +0.089]	0.418	0.502	0.929	0.773
speech_total_pause_dur_delta	−0.001 ± 0.013	0.007 ± 0.037	+0.009 [-0.017, +0.034]	0.495	0.855	0.971	0.966
speech_total_pause_dur_lag1	0.414 ± 0.256	0.378 ± 0.272	-0.035 [-0.145, +0.074]	0.444	0.583	0.957	0.821
speech_total_pause_dur_lag2	0.413 ± 0.258	0.381 ± 0.273	-0.033 [-0.146, +0.080]	0.449	0.391	0.850	0.670
speech_total_pause_dur_roll_std_7d	0.148 ± 0.066	0.169 ± 0.102	+0.021 [-0.023, +0.066]	0.531	0.326	0.815	0.634
speech_f0_min	65.855 ± 0.494	65.915 ± 0.889	+0.061 [-0.279, +0.401]	0.424	0.638	0.957	0.865
speech_hnr	10.556 ± 2.266	10.719 ± 2.247	+0.163 [-0.177, +0.502]	0.531	0.326	0.815	0.634

Data are reported as mean ± standard deviation across subjects, derived from per-patient averages calculated for each clinical phase. Stable: baseline monitoring periods; Prodromal: risk windows immediately preceding a validated exacerbation; Delta: absolute difference computed as $\text{mean}_{\text{prodromal}} - \text{mean}_{\text{stable}}$, presented with its 95% confidence interval [95% CI]; CLES: common language effect size, computed as $P(\text{Prodromal} > \text{Stable})$. Values > 0.50 indicate an increase in the prodromal phase, whereas values < 0.50 indicate a decrease. Statistical significance was assessed using two-sided paired Wilcoxon signed-rank tests. p-value: unadjusted p-value; q-dom: false discovery rate (FDR) adjusted p-value calculated within each physiological domain using the Benjamini–Hochberg procedure. q-glob: FDR adjusted p-value calculated in all features using the Benjamini–Hochberg procedure. Significance levels were assigned based on q-values: ** $q < 0.01$, * $q < 0.05$, and † $q < 0.10$ (exploratory threshold).

Table S4: Physiological, behavioral, and environmental features, evaluated via paired Wilcoxon signed-rank tests with domain- and global-specific Benjamini–Hochberg False Discovery Rate (FDR) corrections. (cont.)

Variable	Stable	Prodromal	Delta [95% CI]	CLES	p-value	q-dom	q-glob
Acoustic Speech							
speech_mean_intensity_delta	−0.081 ± 0.219	0.086 ± 0.258	+0.166 [-0.090, +0.423]	0.643	0.268	0.787	0.585
speech_mean_intensity_roll_mean_3d	−23.754 ± 5.153	−23.209 ± 4.847	+0.545 [-0.195, +1.285]	0.526	0.104	0.594	0.435
speech_mean_intensity_roll_std_7d	1.491 ± 0.769	1.675 ± 0.845	+0.185 [-0.120, +0.489]	0.566	0.268	0.787	0.585
speech_mfcc_1_mean	−303.814 ± 52.724	−301.423 ± 49.456	+2.391 [-6.807, +11.589]	0.500	0.626	0.957	0.861
speech_mfcc_1_std	68.066 ± 11.668	67.265 ± 11.988	-0.800 [-5.717, +4.116]	0.469	0.952	0.971	0.980
speech_mfcc_2_mean	135.691 ± 25.337	136.381 ± 22.363	+0.691 [-3.032, +4.413]	0.505	0.761	0.971	0.932
speech_mfcc_2_std	42.582 ± 6.483	42.586 ± 5.839	+0.004 [-0.965, +0.973]	0.495	0.952	0.971	0.980
speech_mfcc_3_mean	9.665 ± 19.611	11.800 ± 18.337	+2.136 [-1.028, +5.299]	0.531	0.358	0.850	0.652
speech_mfcc_3_std	22.113 ± 3.135	21.680 ± 2.895	-0.434 [-1.395, +0.527]	0.444	0.502	0.929	0.773
speech_mfcc_4_mean	26.931 ± 19.574	28.679 ± 20.002	+1.748 [-0.455, +3.951]	0.520	0.173	0.719	0.498
speech_mfcc_4_std	23.960 ± 3.302	23.796 ± 3.284	-0.164 [-0.906, +0.579]	0.480	0.952	0.971	0.980
speech_mfcc_5_mean	4.956 ± 16.415	4.977 ± 17.838	+0.021 [-2.325, +2.366]	0.510	0.670	0.957	0.875
speech_mfcc_5_std	21.590 ± 2.147	21.438 ± 2.841	-0.152 [-0.899, +0.594]	0.469	0.903	0.971	0.979
speech_mfcc_6_mean	18.722 ± 12.903	15.966 ± 13.795	-2.756 [-4.428, -1.083]	0.434	0.004	0.067 †	0.099 †
speech_mfcc_6_std	15.433 ± 2.753	15.282 ± 2.799	-0.151 [-0.668, +0.367]	0.475	0.391	0.850	0.670
speech_mfcc_7_mean	−11.515 ± 14.871	−10.697 ± 15.849	+0.818 [-0.649, +2.284]	0.520	0.268	0.787	0.585
speech_mfcc_7_std	15.700 ± 3.129	15.324 ± 3.264	-0.377 [-1.005, +0.252]	0.459	0.241	0.787	0.585
speech_mfcc_8_mean	−12.644 ± 10.868	−15.507 ± 8.688	-2.863 [-5.122, -0.604]	0.408	0.001	0.031 *	0.072 †
speech_mfcc_8_std	13.569 ± 1.639	13.427 ± 1.671	-0.142 [-0.629, +0.345]	0.480	0.952	0.971	0.980
speech_mfcc_9_mean	−9.765 ± 4.984	−11.685 ± 5.298	-1.920 [-3.927, +0.087]	0.398	0.035	0.419	0.279
speech_mfcc_9_std	12.191 ± 1.804	12.239 ± 1.654	+0.049 [-0.338, +0.435]	0.480	0.808	0.971	0.961
speech_mfcc_10_mean	−7.230 ± 10.610	−7.236 ± 11.028	-0.006 [-1.654, +1.642]	0.495	0.761	0.971	0.932
speech_mfcc_10_std	11.729 ± 1.442	11.492 ± 1.562	-0.237 [-0.675, +0.201]	0.429	0.463	0.926	0.727
speech_mfcc_11_mean	−12.172 ± 7.710	−12.538 ± 6.128	-0.367 [-1.965, +1.232]	0.510	0.903	0.971	0.979
speech_mfcc_11_std	11.568 ± 1.567	11.487 ± 1.095	-0.081 [-0.586, +0.424]	0.490	0.903	0.971	0.979
speech_mfcc_12_mean	−3.126 ± 7.035	−3.273 ± 7.495	-0.147 [-0.992, +0.699]	0.469	0.583	0.957	0.821
speech_mfcc_12_std	11.799 ± 1.188	11.836 ± 1.120	+0.037 [-0.312, +0.385]	0.546	0.626	0.957	0.861
speech_mfcc_13_mean	−6.302 ± 5.369	−7.101 ± 6.494	-0.799 [-2.083, +0.486]	0.434	0.119	0.594	0.435
speech_mfcc_13_std	10.437 ± 2.019	10.557 ± 1.794	+0.120 [-0.221, +0.462]	0.536	0.855	0.971	0.966
speech_pause_count	1.716 ± 0.691	1.501 ± 0.716	-0.215 [-0.473, +0.043]	0.408	0.119	0.594	0.435
speech_pause_count_delta	0.000 ± 0.065	0.034 ± 0.133	+0.034 [-0.065, +0.132]	0.531	0.463	0.926	0.727
speech_pause_count_lag1	1.713 ± 0.668	1.474 ± 0.694	-0.239 [-0.471, -0.006]	0.398	0.042	0.419	0.293
speech_pause_count_lag2	1.719 ± 0.682	1.503 ± 0.678	-0.216 [-0.450, +0.018]	0.413	0.078	0.594	0.385
speech_pause_count_roll_std_7d	0.596 ± 0.133	0.622 ± 0.228	+0.025 [-0.100, +0.150]	0.541	0.715	0.971	0.907
speech_pitch	143.324 ± 27.990	145.589 ± 26.785	+2.266 [-2.937, +7.468]	0.556	0.583	0.957	0.821
speech_shimmer	0.123 ± 0.020	0.124 ± 0.019	+0.002 [-0.003, +0.006]	0.510	0.761	0.971	0.932
speech_active_duration_delta	−0.004 ± 0.012	0.004 ± 0.024	+0.008 [-0.008, +0.024]	0.648	0.326	0.815	0.634
speech_active_duration_lag2	2.255 ± 0.366	2.220 ± 0.387	-0.034 [-0.172, +0.103]	0.444	1.000	1.000	1.000
speech_active_duration_roll_std_7d	0.127 ± 0.052	0.123 ± 0.043	-0.004 [-0.033, +0.024]	0.500	0.670	0.957	0.875
speech_intensity_var	0.047 ± 0.024	0.050 ± 0.025	+0.004 [+0.001, +0.006]	0.582	0.001	0.031 *	0.072 †
speech_rhythm_var	0.046 ± 0.052	0.043 ± 0.050	-0.004 [-0.034, +0.027]	0.398	0.091	0.594	0.427
speech_pitch_var	13.526 ± 6.619	12.991 ± 7.016	-0.535 [-3.595, +2.525]	0.439	0.135	0.615	0.442
Acoustic Cough							
cough_ber_std	0.645 ± 0.677	1.347 ± 1.932	+0.702 [-0.311, +1.714]	0.561	0.068	0.344	0.360
cough_crest_factor_max	6.759 ± 1.314	7.100 ± 1.445	+0.341 [-0.006, +0.688]	0.566	0.068	0.344	0.360
cough_crest_factor_std	0.733 ± 0.365	0.812 ± 0.417	+0.079 [-0.137, +0.296]	0.551	0.153	0.451	0.481
cough_delta_1_mean_max	0.906 ± 5.068	1.614 ± 5.327	+0.708 [-1.586, +3.002]	0.510	0.670	0.815	0.875
cough_delta_2_mean_max	2.137 ± 1.214	2.477 ± 1.443	+0.340 [-0.325, +1.005]	0.566	0.542	0.731	0.804
cough_delta_2_mean_std	1.606 ± 0.721	1.650 ± 0.918	+0.044 [-0.291, +0.378]	0.526	0.855	0.921	0.966
cough_delta_3_mean_mean	0.919 ± 1.352	0.826 ± 1.198	-0.093 [-0.546, +0.360]	0.469	0.358	0.598	0.652
cough_delta_3_mean_std	1.763 ± 0.723	2.054 ± 0.520	+0.291 [-0.036, +0.619]	0.653	0.119	0.416	0.435
cough_delta_4_mean_mean	-0.274 ± 1.410	-0.466 ± 1.684	-0.192 [-0.484, +0.100]	0.475	0.135	0.421	0.442
cough_delta_4_mean_std	1.421 ± 0.385	1.524 ± 0.590	+0.103 [-0.254, +0.459]	0.587	0.326	0.579	0.634
cough_delta_5_mean_mean	1.661 ± 1.071	1.446 ± 1.054	-0.215 [-0.495, +0.065]	0.459	0.104	0.416	0.435
cough_delta_5_mean_std	1.374 ± 0.606	1.485 ± 0.662	+0.110 [-0.199, +0.419]	0.556	0.326	0.579	0.634
cough_delta_6_mean_mean	0.059 ± 0.649	-0.111 ± 0.736	-0.169 [-0.482, +0.143]	0.393	0.583	0.742	0.821
cough_delta_6_mean_std	1.021 ± 0.380	1.182 ± 0.843	+0.161 [-0.156, +0.478]	0.485	0.463	0.665	0.727
cough_delta_7_mean_max	1.310 ± 0.821	1.324 ± 0.775	+0.014 [-0.270, +0.299]	0.541	0.761	0.888	0.932
cough_delta_7_mean_mean	0.619 ± 0.813	0.558 ± 0.839	-0.061 [-0.412, +0.290]	0.485	0.542	0.731	0.804
cough_delta_7_mean_std	0.960 ± 0.315	1.099 ± 0.528	+0.139 [-0.084, +0.363]	0.556	0.241	0.526	0.585
cough_delta_8_mean_max	0.247 ± 1.070	0.468 ± 1.321	+0.221 [-0.047, +0.489]	0.597	0.119	0.416	0.435
cough_delta_8_mean_std	1.039 ± 0.427	1.154 ± 0.385	+0.115 [-0.028, +0.258]	0.561	0.049	0.344	0.319
cough_delta_9_mean_max	0.430 ± 1.146	0.526 ± 1.138	+0.097 [-0.412, +0.604]	0.551	0.241	0.526	0.585
cough_delta_9_mean_std	0.883 ± 0.405	0.909 ± 0.366	+0.026 [-0.210, +0.262]	0.551	1,000	1,000	1,000
cough_delta_10_mean_max	0.740 ± 0.733	0.897 ± 0.804	+0.157 [-0.078, +0.391]	0.582	0.119	0.416	0.435
cough_delta_10_mean_mean	0.137 ± 0.743	0.160 ± 0.744	+0.023 [-0.150, +0.196]	0.495	0.808	0.905	0.961
cough_delta_10_mean_std	0.826 ± 0.392	1.001 ± 0.566	+0.175 [+0.024, +0.326]	0.571	0.068	0.344	0.360
cough_delta_11_mean_mean	-0.507 ± 0.708	-0.512 ± 0.531	-0.005 [-0.267, +0.257]	0.495	0.583	0.742	0.821
cough_delta_11_mean_std	0.798 ± 0.405	0.802 ± 0.359	+0.005 [-0.149, +0.158]	0.541	0.903	0.963	0.979
cough_delta_12_mean_max	0.710 ± 0.641	0.843 ± 0.508	+0.133 [-0.133, +0.398]	0.571	0.173	0.472	0.498
cough_delta_12_mean_std	0.762 ± 0.320	0.853 ± 0.310	+0.092 [-0.107, +0.290]	0.617	0.135	0.421	0.442
cough_delta_13_mean_max	-0.080 ± 0.505	0.197 ± 0.756	+0.277 [-0.009, +0.562]	0.628	0.078	0.352	0.385
cough_delta_13_mean_std	0.699 ± 0.256	0.777 ± 0.337	+0.078 [-0.030, +0.187]	0.561	0.153	0.551	0.481
cough_dur_max	0.711 ± 0.265	0.744 ± 0.297	+0.034 [-0.029, +0.097]	0.561	0.268	0.526	0.585
cough_dur_std	0.116 ± 0.099	0.133 ± 0.137	+0.017 [-0.018, +0.053]	0.500	0.391	0.626	0.670
cough_f0_mean_mean	344.072 ± 71.692	352.769 ± 82.855	+8.696 [-19.216, +36.608]	0.551	0.463	0.665	0.727
cough_f0_std_max	179.694 ± 34.425	187.287 ± 37.826	+7.593 [-14.806, +29.991]	0.582	0.358	0.598	0.652
cough_f0_std_mean	142.281 ± 24.764	141.513 ± 24.185	-0.767 [-18.485, +16.949]	0.495	0.583	0.742	0.821
cough_f1_mean_max	812.705 ± 119.455	849.052 ± 154.471	+36.348 [-13.958, +86.654]	0.607	0.135	0.421	0.442
cough_f1_mean_std	123.355 ± 52.531	124.617 ± 79.752	+1.262 [-36.948, +39.473]	0.475	0.855	0.921	0.966
cough_f2_mean_max	1731.012 ± 179.050	1761.683 ± 170.458	+30.672 [-3.517, +64.860]	0.551	0.068	0.344	0.360
cough_f2_mean_std	144.967 ± 58.382	139.400 ± 56.648	-5.567 [-30.194, +19.060]	0.546	0.808	0.905	0.961
cough_f3_mean_max	2572.089 ± 188.043	2626.467 ± 156.429	+54.377 [+5.298, +103.457]	0.602	0.025	0.331	0.240
cough_f3_mean_std	186.573 ± 51.759	204.829 ± 45.748	+18.256 [-10.530, +47.043]	0.658	0.241	0.526	0.585
cough_hnr_mean_max	3.904 ± 1.593	4.208 ± 1.631	+0.304 [-0.222, +0.829]	0.546	0.194	0.505	0.546
cough_hnr_mean_mean_delta	-0.021 ± 0.080	-0.026 ± 0.169	-0.005 [-0.129, +0.119]	0.536	0.761	0.888	0.932
cough_hnr_mean_mean_lag2	2.952 ± 1.383	3.008 ± 1.393	+0.056 [-0.095, +0.206]	0.505	0.326	0.579	0.634
cough_hnr_mean_mean_roll_std_7d	0.904 ± 0.278	0.945 ± 0.352	+0.041 [-0.147, +0.229]	0.541	0.808	0.905	0.961
cough_hnr_mean_std	1.437 ± 0.500	1.535 ± 0.504	+0.098 [-0.147, +0.344]	0.597	0.358	0.598	0.652
cough_jitter_max	0.061 ± 0.011	0.065 ± 0.012	+0.004 [+0.001, +0.007]	0.587	0.030	0.331	0.256
cough_jitter_std	0.015 ± 0.007	0.017 ± 0.010	+0.002 [-0.001, +0.004]	0.566	0.241	0.526	0.585
cough_kurtosis_std	2.263 ± 1.684	2.388 ± 1.853	+0.125 [-0.830, +1.080]	0.505	0.952	0.969	0.980
cough_log_energy_mean_std	0.165 ± 0.055	0.196 ± 0.099	+0.032 [-0.012, +0.075]	0.571	0.078	0.352	0.385
cough_mfcc_1_mean_mean	-192.009 ± 29.341	-198.829 ± 29.463	-6.821 [-12.347, -1.294]	0.444	0.025	0.331	0.240
cough_mfcc_1_mean_std	15.908 ± 5.089	21.209 ± 8.485	+5.301 [+1.206, +9.395]	0.725	0.003	0.226	0.099 †
cough_mfcc_1_std_mean	96.644 ± 12.983	94.466 ± 12.868	-2.178 [-4.287, -0.070]	0.434	0.025	0.331	0.240
cough_mfcc_1_std_std	9.419 ± 3.750	10.673 ± 5.365	+1.254 [-1.685, +4.193]	0.582	0.463	0.665	0.727

Data are reported as mean ± standard deviation across subjects, derived from per-patient averages calculated for each clinical phase. Stable: baseline monitoring periods; Prodromal: risk windows immediately preceding a validated exacerbation; Delta: absolute difference computed as $\text{mean}_{\text{prodromal}} - \text{mean}_{\text{stable}}$, presented with its 95% confidence interval [95% CI]; CLES: common language effect size, computed as $P(\text{Prodromal} > \text{Stable})$. Values > 0.50 indicate an increase in the prodromal phase, whereas values < 0.50 indicate a decrease. Statistical significance was assessed using two-sided paired Wilcoxon signed-rank tests. p-value: unadjusted p-value; q-dom: false discovery rate (FDR) adjusted p-value calculated within each physiological domain using the Benjamini–Hochberg procedure. q-glob: FDR adjusted p-value calculated in all features using the Benjamini–Hochberg procedure. Significance levels were assigned based on q-values: ** $q < 0.01$, * $q < 0.05$, and † $q < 0.10$ (exploratory threshold).

Table S4: Physiological, behavioral, and environmental features, evaluated via paired Wilcoxon signed-rank tests with domain- and global-specific Benjamini–Hochberg False Discovery Rate (FDR) corrections. (cont.)

Variable	Stable	Prodromal	Delta [95% CI]	CLES	p-value	q-dom	q-glob
Acoustic Cough							
cough_mfcc_2_mean_max	162.363 ± 16.953	163.942 ± 18.892	+1.580 [-2.146, +5.305]	0.526	0.426	0.645	0.701
cough_mfcc_2_mean_std	7.982 ± 2.165	9.416 ± 3.001	+1.434 [-0.191, +3.058]	0.653	0.042	0.335	0.293
cough_mfcc_2_std_max	31.426 ± 4.322	31.263 ± 4.224	-0.163 [-2.101, +1.775]	0.495	0.626	0.787	0.861
cough_mfcc_2_std_std	4.220 ± 1.058	4.765 ± 1.498	+0.545 [-0.172, +1.262]	0.582	0.268	0.526	0.585
cough_mfcc_3_mean_max	-83.474 ± 18.070	-78.603 ± 18.288	+4.871 [+0.562, +9.180]	0.592	0.058	0.344	0.355
cough_mfcc_3_mean_std	9.053 ± 2.483	9.509 ± 3.390	+0.456 [-0.981, +1.893]	0.561	0.426	0.645	0.701
cough_mfcc_3_std_mean	37.042 ± 5.746	35.892 ± 5.281	-1.150 [-2.425, +0.126]	0.424	0.058	0.344	0.355
cough_mfcc_3_std_std	4.577 ± 1.131	4.843 ± 1.932	+0.266 [-0.545, +1.077]	0.577	0.583	0.742	0.821
cough_mfcc_4_mean_mean	42.846 ± 16.094	43.186 ± 18.285	+0.340 [-3.968, +4.647]	0.495	0.952	0.969	0.980
cough_mfcc_4_mean_std	7.029 ± 1.634	8.621 ± 3.428	+1.593 [-0.077, +3.263]	0.638	0.091	0.390	0.427
cough_mfcc_4_std_max	21.322 ± 2.473	22.401 ± 3.091	+1.079 [+0.109, +2.048]	0.587	0.042	0.335	0.293
cough_mfcc_4_std_std	3.183 ± 0.642	3.404 ± 1.003	+0.222 [-0.452, +0.895]	0.582	0.391	0.626	0.670
cough_mfcc_5_mean_mean	-36.808 ± 11.633	-38.237 ± 12.561	-1.429 [-4.068, +1.211]	0.439	0.173	0.472	0.498
cough_mfcc_5_mean_std	6.619 ± 2.242	7.954 ± 3.906	+1.335 [-0.401, +3.070]	0.582	0.268	0.526	0.585
cough_mfcc_5_std_mean	21.927 ± 3.274	21.343 ± 4.227	-0.584 [-1.557, +0.389]	0.469	0.391	0.626	0.670
cough_mfcc_5_std_std	3.460 ± 1.115	3.407 ± 1.464	-0.053 [-0.586, +0.480]	0.459	0.952	0.969	0.980
cough_mfcc_6_mean_mean	-14.163 ± 14.507	-15.945 ± 13.603	-1.782 [-4.870, +1.306]	0.469	0.296	0.552	0.614
cough_mfcc_6_mean_std	4.981 ± 1.468	6.114 ± 1.882	+1.133 [+0.467, +1.800]	0.679	0.004	0.226	0.099 †
cough_mfcc_6_std_max	13.531 ± 2.334	13.874 ± 2.857	+0.343 [-0.385, +1.070]	0.551	0.268	0.526	0.585
cough_mfcc_6_std_std	2.572 ± 0.600	2.658 ± 0.884	+0.085 [-0.346, +0.516]	0.510	0.952	0.969	0.980
cough_mfcc_7_mean_mean	-17.253 ± 7.986	-18.347 ± 8.346	-1.093 [-3.498, +1.311]	0.449	0.426	0.645	0.701
cough_mfcc_7_mean_std	4.932 ± 1.266	4.839 ± 2.019	-0.093 [-0.961, +0.775]	0.464	0.670	0.815	0.875
cough_mfcc_7_std_mean	12.064 ± 1.422	12.225 ± 2.375	+0.161 [-1.100, +1.422]	0.495	1.000	1.000	1.000
cough_mfcc_7_std_std	2.849 ± 0.365	3.139 ± 0.981	+0.290 [-0.319, +0.898]	0.577	0.502	0.711	0.773
cough_mfcc_8_mean_max	-27.395 ± 8.499	-27.139 ± 8.882	+0.257 [-1.873, +2.386]	0.526	0.952	0.969	0.980
cough_mfcc_8_mean_std	5.313 ± 2.352	5.531 ± 2.645	+0.218 [-0.329, +0.765]	0.536	0.583	0.742	0.821
cough_mfcc_8_std_mean	14.123 ± 3.066	13.679 ± 3.365	-0.444 [-1.075, +0.187]	0.454	0.173	0.472	0.498
cough_mfcc_8_std_std	2.686 ± 0.981	2.915 ± 0.994	+0.230 [-0.176, +0.636]	0.551	0.358	0.598	0.652
cough_mfcc_9_mean_mean	1.948 ± 10.809	0.207 ± 11.171	-1.741 [-3.517, +0.035]	0.434	0.078	0.352	0.385
cough_mfcc_9_mean_std	4.320 ± 1.217	5.481 ± 2.006	+1.161 [+0.404, +1.919]	0.663	0.007	0.251	0.125
cough_mfcc_9_std_max	12.274 ± 3.697	12.384 ± 4.544	+0.110 [-0.830, +1.050]	0.510	0.241	0.526	0.585
cough_mfcc_9_std_std	2.506 ± 0.628	2.335 ± 0.983	-0.172 [-0.610, +0.267]	0.408	0.670	0.815	0.875
cough_mfcc_9_mean_mean	-17.632 ± 5.511	-18.841 ± 6.344	-1.209 [-2.289, -0.129]	0.454	0.049	0.344	0.319
cough_mfcc_9_mean_std	4.061 ± 1.412	5.176 ± 1.835	+1.115 [+0.301, +1.928]	0.679	0.020	0.331	0.240
cough_mfcc_9_std_mean	11.873 ± 1.658	11.249 ± 1.352	-0.624 [-1.581, +0.332]	0.403	0.268	0.526	0.585
cough_mfcc_11_mean_max	-3.903 ± 6.585	-3.075 ± 7.630	+0.828 [-0.799, +2.455]	0.566	0.194	0.505	0.546
cough_mfcc_11_mean_std	4.755 ± 1.392	5.691 ± 2.784	+0.936 [-0.390, +2.261]	0.607	0.268	0.526	0.585
cough_mfcc_11_std_mean	9.493 ± 2.102	9.068 ± 1.564	-0.425 [-1.147, +0.297]	0.449	0.296	0.552	0.614
cough_mfcc_11_std_std	2.276 ± 0.545	1.973 ± 0.607	-0.303 [-0.726, +0.119]	0.372	0.241	0.526	0.585
cough_mfcc_12_mean_max	-8.959 ± 7.722	-7.423 ± 7.827	+1.535 [-0.367, +3.438]	0.556	0.119	0.416	0.435
cough_mfcc_12_mean_std	3.835 ± 0.825	4.768 ± 2.529	+0.933 [-0.372, +2.237]	0.658	0.241	0.526	0.585
cough_mfcc_12_std_mean	9.333 ± 1.341	9.090 ± 1.152	-0.242 [-0.528, +0.044]	0.469	0.104	0.416	0.435
cough_mfcc_12_std_std	1.921 ± 0.374	2.064 ± 1.112	+0.143 [-0.379, +0.666]	0.541	0.542	0.731	0.804
cough_mfcc_13_mean_max	-9.112 ± 5.480	-7.199 ± 4.430	+1.913 [+0.282, +3.543]	0.679	0.035	0.335	0.279
cough_mfcc_13_mean_std	3.990 ± 1.198	4.296 ± 1.480	+0.306 [-0.470, +1.083]	0.556	0.542	0.731	0.804
cough_mfcc_13_std_mean	10.194 ± 1.754	9.943 ± 1.666	-0.251 [-1.181, +0.678]	0.495	0.808	0.905	0.961
cough_mfcc_13_std_std	2.096 ± 0.565	2.240 ± 0.677	+0.143 [-0.187, +0.474]	0.546	0.426	0.645	0.701
cough_mfcc_9_std_std	2.224 ± 0.507	2.265 ± 1.011	+0.041 [-0.455, +0.536]	0.490	0.761	0.888	0.932
cough_shimmer_std	0.045 ± 0.023	0.047 ± 0.023	+0.001 [-0.005, +0.008]	0.520	0.715	0.861	0.907
cough_skewness_mean	0.247 ± 0.277	0.236 ± 0.183	-0.011 [-0.095, +0.073]	0.541	0.296	0.552	0.614
cough_skewness_std	0.195 ± 0.155	0.180 ± 0.068	-0.015 [-0.090, +0.060]	0.582	0.855	0.921	0.966
cough_spec_centroid_mean_mean	1311.047 ± 197.409	1296.948 ± 201.052	-14.099 [-54.825, +26.627]	0.464	0.463	0.665	0.727
cough_spec_centroid_std_mean	266.648 ± 96.789	249.042 ± 78.313	-17.605 [-34.213, -0.998]	0.449	0.030	0.331	0.256
cough_spec_centroid_std_std	68.560 ± 30.847	57.700 ± 21.341	-10.860 [-24.571, +2.850]	0.429	0.268	0.526	0.585
cough_spec_entropy_mean	5.888 ± 0.454	5.759 ± 0.374	-0.129 [-0.301, +0.043]	0.413	0.135	0.421	0.442
cough_spec_entropy_std	0.311 ± 0.130	0.416 ± 0.277	+0.106 [-0.031, +0.242]	0.597	0.042	0.335	0.293
cough_spec_rolloff_mean_std	181.022 ± 53.826	209.795 ± 81.608	+28.773 [-1.517, +59.063]	0.643	0.025	0.331	0.240
cough_zcr_mean_std	0.015 ± 0.006	0.015 ± 0.008	-0.000 [-0.004, +0.004]	0.469	0.855	0.921	0.966
Indoor Air Quality, Environment							
co2_mean_day	661.140 ± 163.220	715.947 ± 219.014	+54.807 [−9.177, +118.792]	0.556	0.049	0.272	0.319
co2_min_24h	403.360 ± 16.499	420.607 ± 37.196	+17.247 [−3.894, +38.388]	0.617	0.135	0.298	0.442
co2_std_night	135.287 ± 96.353	150.175 ± 102.889	+14.887 [−10.636, +40.411]	0.536	0.153	0.306	0.481
hum_min_24h	52.107 ± 4.711	54.380 ± 6.286	+2.273 [−1.148, +5.694]	0.602	0.296	0.434	0.614
hum_std_night	1.858 ± 0.518	1.568 ± 0.503	−0.291 [−0.465, −0.116]	0.332	0.004	0.074†	0.099†
hum_std_24h	2.681 ± 0.512	2.308 ± 0.596	−0.373 [−0.652, −0.095]	0.321	0.025	0.180	0.240
ppm10_max_night	16.342 ± 12.565	17.054 ± 12.893	+0.712 [−2.684, +4.109]	0.479	1.000	1.000	1.000
ppm10_max_24h	86.932 ± 95.116	91.713 ± 102.510	+4.782 [−14.941, +24.504]	0.495	0.903	0.946	0.979
ppm25_min_24h	5.478 ± 5.928	4.792 ± 5.005	−0.687 [−1.707, +0.334]	0.469	0.241	0.408	0.585
ppm4_min_day	4.805 ± 3.370	4.902 ± 4.037	+0.097 [−0.538, +0.732]	0.434	0.808	0.935	0.961
ppm4_min_night	9.364 ± 12.723	6.708 ± 7.765	−2.656 [−6.325, +1.013]	0.444	0.119	0.298	0.435
temp_max_night	25.631 ± 2.521	23.790 ± 3.112	−1.841 [−3.003, −0.680]	0.332	0.007	0.074†	0.125
temp_std_night	0.414 ± 0.270	0.364 ± 0.223	−0.051 [−0.111, +0.010]	0.459	0.104	0.298	0.435
temp_std_24h	0.586 ± 0.226	0.497 ± 0.235	−0.089 [−0.176, −0.002]	0.378	0.068	0.298	0.360
voc_max_day	400.035 ± 40.910	378.013 ± 46.146	−22.021 [−53.918, +9.875]	0.357	0.268	0.420	0.585
voc_mean_night	136.087 ± 38.947	128.802 ± 35.819	−7.285 [−24.129, +9.559]	0.444	0.463	0.599	0.727
voc_mean_24h	141.586 ± 11.305	134.954 ± 14.214	−6.631 [−15.917, +2.655]	0.367	0.173	0.316	0.498
voc_min_day	24.908 ± 6.280	28.505 ± 10.598	+3.597 [−0.790, +7.983]	0.556	0.119	0.298	0.435
voc_min_night	62.543 ± 20.718	61.675 ± 22.919	−0.868 [−11.368, +9.631]	0.449	0.855	0.941	0.966
voc_min_24h	23.539 ± 5.552	26.695 ± 9.273	+3.156 [−1.089, +7.401]	0.587	0.358	0.492	0.652
voc_std_night	55.458 ± 15.675	54.717 ± 18.722	−0.741 [−7.668, +6.186]	0.469	0.715	0.874	0.907
voc_std_24h	99.698 ± 10.310	93.186 ± 16.109	−6.512 [−14.223, +1.198]	0.357	0.135	0.298	0.442

Data are reported as mean ± standard deviation across subjects, derived from per-patient averages calculated for each clinical phase. Stable: baseline monitoring periods; Prodromal: risk windows immediately preceding a validated exacerbation; Delta: absolute difference computed as $\text{mean}_{\text{prodromal}} - \text{mean}_{\text{stable}}$, presented with its 95% confidence interval [95% CI]; CLES: common language effect size, computed as $P(\text{Prodromal} > \text{Stable})$. Values > 0.50 indicate an increase in the prodromal phase, whereas values < 0.50 indicate a decrease. Statistical significance was assessed using two-sided paired Wilcoxon signed-rank tests. p-value: unadjusted p-value; q-dom: false discovery rate (FDR) adjusted p-value calculated within each physiological domain using the Benjamini–Hochberg procedure. q-glob: FDR adjusted p-value calculated in all features using the Benjamini–Hochberg procedure. Significance levels were assigned based on q-values: ** $q < 0.01$, * $q < 0.05$, and † $q < 0.10$ (exploratory threshold).
