# Supplementary material for: Multimodal Prodromal Patterns Associated with COPD Exacerbations in Home Telemonitoring: An Exploratory Observational Study
Source: Bioengineering (Basel). 2026 Oct 14;13(10):1195. doi: 10.3390/bioengineering13101195 (PMC13701270; doi:10.3390/bioengineering13101195)
Supplement: Supplementary file 1 [file bioengineering-13-01195-s001.zip › Supplementary Material S5.pdf]

Supplementary Material S5

Table S5: Physiological, behavioral, and environmental features, evaluated via Linear Mixed Models (LMM) with domain- and global-specific Benjamini–Hochberg False Discovery Rate (FDR) corrections.

**Domain / Feature**	β **[95% CI]**	p**-value**	q**-dom**	q**-glob**	$\sigma^2_{\text{intercept}}$	σ^2_{slope}	$\sigma^2_{\text{residual}}$	**ICC**
Patient Reported Symptoms								
q1_general_health	0.390 [0.172, 0.608]	< 0.001	0.002**	0.019*	0.877	0.172	0.245	0.782
q2_dyspnea_delta	0.074 [-0.040, 0.189]	0.204	0.239	0.508	0.000	0.000	1.000	0.000
q2_dyspnea_roll_mean_3d	0.271 [0.033, 0.509]	0.025	0.040*	0.207	0.972	0.209	0.236	0.805
q2_dyspnea_roll_std_7d	0.117 [-0.211, 0.446]	0.484	0.521	0.741	0.466	0.385	0.712	0.395
q3_cough_delta	0.222 [0.056, 0.388]	0.009	0.016*	0.115	0.000	0.053	0.993	0.000
q3_cough_roll_mean_3d	0.344 [0.196, 0.493]	< 0.001	< 0.001***	0.001**	1.018	0.074	0.185	0.846
q3_cough_roll_std_7d	0.563 [0.218, 0.909]	0.001	0.003**	0.037*	0.248	0.425	0.762	0.245
q4_rescue_inhaler_delta	0.074 [-0.025, 0.173]	0.142	0.180	0.446	0.000	0.000	1.000	0.000
q4_rescue_inhaler_roll_mean_3d	0.056 [-0.141, 0.253]	0.578	0.578	0.789	0.848	0.142	0.166	0.836
q4_rescue_inhaler_roll_std_7d	0.313 [-0.016, 0.641]	0.062	0.087†	0.339	0.160	0.372	0.784	0.170
q5_sputum_volume_delta	0.232 [0.095, 0.369]	0.001	0.003**	0.027*	0.000	0.023	0.993	0.000
q5_sputum_volume_roll_mean_3d	0.249 [0.135, 0.364]	< 0.001	< 0.001***	0.002**	0.860	0.040	0.172	0.833
q5_sputum_volume_roll_std_7d	0.635 [0.240, 1.031]	0.002	0.003**	0.037*	0.188	0.567	0.725	0.206
q6_sputum_color	0.281 [0.147, 0.415]	< 0.001	< 0.001***	0.002**	0.697	0.059	0.133	0.840
Cardiovascular								
hrv_daily_rmssd	-0.078 [-0.173, 0.016]	0.105	0.131	0.428	0.732	0.002	0.435	0.627
hrv_deep_rmssd	0.022 [-0.093, 0.136]	0.708	0.708	0.861	0.246	0.008	0.717	0.255
resting_hr	0.140 [0.031, 0.249]	0.012	0.059†	0.121	0.734	0.038	0.113	0.867
resting_hr_delta	0.158 [0.002, 0.315]	0.047	0.078†	0.301	0.000	0.000	0.998	0.000
resting_hr_roll_std_7d	0.477 [0.053, 0.900]	0.027	0.068†	0.209	0.107	0.636	0.869	0.109
Nocturnal Respiratory Dynamics								
avg_breathing_rate	0.009 [-0.194, 0.212]	0.931	0.931	0.971	0.781	0.113	0.626	0.555
deep_breathing_rate	0.208 [-0.100, 0.517]	0.186	0.232	0.482	0.683	0.317	0.650	0.512
light_breathing_rate	-0.152 [-0.316, 0.012]	0.069	0.232	0.339	0.984	0.063	0.568	0.634
rem_breathing_rate	0.142 [-0.035, 0.320]	0.117	0.232	0.446	0.789	0.059	0.723	0.522
spo2_mean	-0.101 [-0.246, 0.044]	0.171	0.232	0.465	0.363	0.038	0.634	0.364
Skin Temperature								
relative_skin_temp	0.149 [-0.021, 0.318]	0.086	0.086†	0.385	0.021	0.052	0.979	0.021
Actigraphy								
fairly_active_mins	-0.042 [-0.217, 0.134]	0.641	0.819	0.831	0.234	0.073	0.808	0.225
lightly_active_mins	0.057 [-0.169, 0.282]	0.623	0.819	0.821	0.535	0.164	0.522	0.506
sedentary_mins_delta	0.019 [-0.090, 0.128]	0.735	0.819	0.870	0.000	0.000	1.000	0.000
sedentary_mins_lag2	0.161 [-0.078, 0.401]	0.187	0.814	0.482	0.486	0.181	0.685	0.415
sedentary_mins_roll_std_7d	0.116 [-0.186, 0.419]	0.451	0.814	0.730	0.157	0.308	0.888	0.150
steps	-0.077 [-0.279, 0.125]	0.455	0.814	0.730	0.434	0.125	0.541	0.445
steps_delta	-0.006 [-0.115, 0.103]	0.914	0.914	0.971	0.000	0.000	1.000	0.000
steps_lag1	-0.074 [-0.260, 0.111]	0.432	0.814	0.730	0.433	0.100	0.541	0.445
steps_lag2	-0.076 [-0.258, 0.106]	0.412	0.814	0.721	0.439	0.095	0.537	0.450
steps_roll_mean_3d	-0.083 [-0.310, 0.144]	0.475	0.814	0.741	0.574	0.176	0.388	0.597
steps_roll_std_7d	-0.032 [-0.226, 0.163]	0.751	0.819	0.884	0.570	0.122	0.419	0.577
very_active_mins	-0.113 [-0.259, 0.033]	0.128	0.814	0.446	0.289	0.044	0.772	0.273
Fine Motor								
draw_circularity_delta	-0.039 [-0.222, 0.144]	0.677	0.910	0.860	0.019	0.053	0.985	0.019
draw_circularity_lag1	-0.217 [-0.522, 0.089]	0.165	0.727	0.465	0.262	0.289	0.749	0.259
draw_circularity_lag2	-0.184 [-0.460, 0.093]	0.193	0.727	0.492	0.257	0.226	0.756	0.254
draw_circularity_roll_std_7d	-0.012 [-0.213, 0.188]	0.904	0.969	0.971	0.292	0.097	0.801	0.267
draw spatial deviation	0.053 [-0.067, 0.174]	0.387	0.727	0.706	0.442	0.010	0.682	0.393
tapping_err_mean_delta	0.022 [-0.089, 0.133]	0.696	0.910	0.860	0.000	0.000	1.000	0.000
tapping_err_mean_lag1	-0.125 [-0.410, 0.160]	0.389	0.727	0.706	0.895	0.210	0.875	0.506
tapping_err_mean_lag2	-0.173 [-0.442, 0.096]	0.206	0.727	0.508	0.721	0.202	0.882	0.450
tapping_err_mean_roll_std_7d	0.097 [-0.195, 0.389]	0.514	0.795	0.764	0.313	0.246	0.811	0.279
tapping_err1	-0.001 [-0.144, 0.142]	0.989	0.989	0.993	0.102	0.019	0.925	0.099
tapping_err2	-0.007 [-0.133, 0.119]	0.912	0.969	0.971	0.084	0.005	0.962	0.080
tapping_err3	-0.187 [-0.650, 0.275]	0.427	0.727	0.727	1.628	0.644	0.837	0.661
draw_eccentricity	-0.067 [-0.190, 0.056]	0.287	0.727	0.611	0.554	0.036	0.277	0.667
draw_smoothness	0.230 [-0.075, 0.534]	0.139	0.727	0.446	0.206	0.282	0.796	0.206
tapping_time	-0.078 [-0.234, 0.078]	0.328	0.727	0.658	0.792	0.076	0.198	0.800
draw_radius_mean	-0.063 [-0.177, 0.051]	0.280	0.727	0.602	0.861	0.027	0.278	0.756
draw_radius_std	-0.015 [-0.137, 0.108]	0.814	0.969	0.915	0.694	0.034	0.293	0.703
Sleep Architecture								
awakenings_count	-0.116 [-0.272, 0.040]	0.146	0.822	0.446	0.181	0.042	0.849	0.176
deep_sleep_mins	0.017 [-0.218, 0.252]	0.889	0.960	0.968	0.196	0.154	0.865	0.185
sleep_efficiency	0.054 [-0.093, 0.200]	0.474	0.822	0.741	0.386	0.048	0.525	0.424
rem_sleep_mins	-0.079 [-0.353, 0.194]	0.569	0.822	0.789	0.183	0.228	0.854	0.176
time_in_bed	0.071 [-0.184, 0.326]	0.587	0.822	0.790	0.212	0.207	0.701	0.232
total_asleep_mins	0.004 [-0.147, 0.155]	0.960	0.960	0.988	0.392	0.059	0.437	0.473
wake_mins	0.093 [-0.143, 0.329]	0.439	0.822	0.730	0.235	0.165	0.727	0.244

CI: Confidence Interval; ICC: Intraclass Correlation Coefficient; $\sigma^2_{\text{intercept}}$: per-patient random intercept variance; σ^2_{slope}: random slope variance for prodromal status; $\sigma^2_{\text{residual}}$: residual variance. All biomarkers were globally standardized (Z-score) prior to LMM fitting. p-value: unadjusted p-value; q-domain: false discovery rate (FDR) adjusted p-value calculated within each domain using the Benjamini–Hochberg procedure. Significance levels were assigned based on q-values: *** $q < 0.001$, ** $q < 0.01$, * $q < 0.05$, and † $q < 0.10$ (exploratory threshold).

Table S5: Physiological, behavioral, and environmental features, evaluated via Linear Mixed Models (LMM) with domain- and global-specific Benjamini–Hochberg False Discovery Rate (FDR) corrections. (cont.)

**Domain / Feature**	β **[95% CI]**	p**-value**	q**-dom**	q**-glob**	$\sigma^2_{\text{intercept}}$	σ^2_{slope}	$\sigma^2_{\text{residual}}$	**ICC**
Acoustic Speech								
speech_duration_delta	-0.013 [-0.115, 0.089]	0.798	0.956	0.911	0.000	0.000	1.000	0.000
speech_total_pause_dur	-0.112 [-0.435, 0.211]	0.496	0.953	0.754	0.640	0.337	0.560	0.533
speech_total_pause_dur_delta	-0.012 [-0.121, 0.097]	0.826	0.961	0.924	0.000	0.000	1.000	0.000
speech_total_pause_dur_lag1	-0.128 [-0.432, 0.176]	0.411	0.953	0.721	0.641	0.294	0.565	0.532
speech_total_pause_dur_lag2	-0.120 [-0.434, 0.194]	0.453	0.953	0.730	0.656	0.315	0.563	0.538
speech_total_pause_dur_roll_std_7d	0.176 [-0.162, 0.514]	0.307	0.903	0.632	0.323	0.361	0.745	0.302
speech_f0_min	0.022 [-0.229, 0.273]	0.862	0.980	0.952	0.184	0.173	0.723	0.203
speech_hnr	0.093 [-0.030, 0.217]	0.139	0.586	0.446	0.919	0.030	0.396	0.699
speech_mean_intensity_delta	0.041 [-0.072, 0.154]	0.479	0.953	0.741	0.000	0.000	1.000	0.000
speech_mean_intensity_roll_mean_3d	0.122 [-0.002, 0.245]	0.054	0.448	0.338	1.018	0.048	0.127	0.889
speech_mean_intensity_roll_std_7d	0.195 [-0.069, 0.459]	0.148	0.586	0.446	0.574	0.210	0.641	0.473
speech_mfcc_1_mean	0.058 [-0.078, 0.195]	0.404	0.953	0.717	0.823	0.052	0.264	0.757
speech_mfcc_1_std	-0.079 [-0.375, 0.217]	0.601	0.956	0.800	0.940	0.273	0.563	0.625
speech_mfcc_10_mean	-0.002 [-0.128, 0.125]	0.978	0.995	0.993	1.061	0.044	0.204	0.839
speech_mfcc_10_std	-0.093 [-0.295, 0.109]	0.366	0.953	0.695	0.513	0.110	0.543	0.486
speech_mfcc_11_mean	-0.028 [-0.180, 0.124]	0.720	0.956	0.866	0.991	0.063	0.401	0.712
speech_mfcc_11_std	0.001 [-0.245, 0.247]	0.995	0.995	0.995	0.679	0.198	0.511	0.570
speech_mfcc_12_mean	-0.020 [-0.120, 0.081]	0.702	0.956	0.860	0.964	0.018	0.298	0.764
speech_mfcc_12_std	0.040 [-0.155, 0.235]	0.688	0.956	0.860	0.640	0.087	0.674	0.487
speech_mfcc_13_mean	-0.132 [-0.300, 0.036]	0.124	0.586	0.446	0.581	0.075	0.461	0.557
speech_mfcc_13_std	0.030 [-0.122, 0.183]	0.698	0.956	0.860	0.910	0.064	0.335	0.731
speech_mfcc_2_mean	0.030 [-0.093, 0.153]	0.634	0.956	0.830	1.130	0.048	0.142	0.889
speech_mfcc_2_std	0.001 [-0.111, 0.112]	0.987	0.995	0.993	0.647	0.021	0.435	0.598
speech_mfcc_3_mean	0.095 [-0.035, 0.224]	0.152	0.586	0.449	0.905	0.045	0.242	0.790
speech_mfcc_3_std	-0.115 [-0.313, 0.083]	0.255	0.814	0.574	0.525	0.106	0.539	0.494
speech_mfcc_4_mean	0.084 [-0.020, 0.188]	0.112	0.586	0.435	1.049	0.024	0.226	0.823
speech_mfcc_4_std	-0.030 [-0.191, 0.130]	0.710	0.956	0.861	0.689	0.066	0.412	0.626
speech_mfcc_5_mean	0.014 [-0.095, 0.124]	0.797	0.956	0.911	1.046	0.027	0.251	0.806
speech_mfcc_5_std	-0.012 [-0.203, 0.179]	0.903	0.995	0.971	0.455	0.100	0.487	0.483
speech_mfcc_6_mean	-0.196 [-0.323, -0.070]	0.002	0.059†	0.045*	0.937	0.037	0.292	0.763
speech_mfcc_6_std	-0.037 [-0.166, 0.092]	0.572	0.954	0.789	0.733	0.039	0.334	0.687
speech_mfcc_7_mean	0.049 [-0.036, 0.133]	0.261	0.814	0.575	0.912	0.014	0.184	0.832
speech_mfcc_7_std	-0.095 [-0.259, 0.069]	0.255	0.814	0.574	1.008	0.078	0.260	0.795
speech_mfcc_8_mean	-0.272 [-0.460, -0.085]	0.004	0.073†	0.067†	1.253	0.102	0.414	0.751
speech_mfcc_8_std	-0.063 [-0.260, 0.135]	0.535	0.954	0.778	0.519	0.106	0.526	0.496
speech_mfcc_9_mean	-0.218 [-0.404, -0.033]	0.021	0.264	0.191	0.477	0.093	0.430	0.526
speech_mfcc_9_std	0.022 [-0.154, 0.199]	0.803	0.956	0.911	0.706	0.084	0.457	0.607
speech_pause_count	-0.211 [-0.443, 0.021]	0.075	0.466	0.351	0.391	0.151	0.656	0.374
speech_pause_count_delta	0.016 [-0.094, 0.126]	0.770	0.956	0.899	0.000	0.000	1.000	0.000
speech_pause_count_lag1	-0.235 [-0.454, -0.016]	0.036	0.356	0.251	0.374	0.130	0.660	0.362
speech_pause_count_lag2	-0.205 [-0.424, 0.013]	0.066	0.466	0.339	0.387	0.130	0.661	0.369
speech_pause_count_roll_std_7d	0.110 [-0.235, 0.454]	0.533	0.954	0.778	0.242	0.354	0.885	0.215
speech_pitch	0.074 [-0.078, 0.227]	0.340	0.945	0.666	0.771	0.068	0.245	0.759
speech_shimmer	0.058 [-0.098, 0.214]	0.466	0.953	0.740	0.721	0.056	0.501	0.590
speech_active_duration_delta	-0.004 [-0.136, 0.129]	0.957	0.995	0.988	0.000	0.000	1.000	0.000
speech_active_duration_lag2	-0.143 [-0.515, 0.229]	0.451	0.953	0.730	1.780	0.456	0.292	0.859
speech_active_duration_roll_std_7d	0.016 [-0.321, 0.352]	0.927	0.995	0.971	0.528	0.377	0.779	0.404
speech_intensity_var	0.123 [0.071, 0.174]	< 0.001	< 0.001***	0.001**	0.903	0.001	0.157	0.852
speech_rhythm_var	-0.115 [-0.506, 0.276]	0.564	0.954	0.789	0.651	0.517	0.699	0.482
speech_pitch_var	-0.029 [-0.245, 0.188]	0.794	0.956	0.911	0.243	0.118	0.820	0.228
Acoustic Cough								
cough_band_energy_ratio_std	0.336 [-0.112, 0.784]	0.142	0.423	0.446	0.127	0.670	0.819	0.134
cough_crest_factor_max	0.179 [-0.005, 0.363]	0.057	0.310	0.339	0.457	0.091	0.486	0.485
cough_crest_factor_std	0.084 [-0.188, 0.356]	0.546	0.728	0.778	0.227	0.218	0.772	0.227
cough_delta_0_mean_max	0.093 [-0.166, 0.352]	0.483	0.702	0.741	0.362	0.199	0.713	0.337
cough_delta_1_mean_max	0.136 [-0.089, 0.361]	0.237	0.510	0.553	0.228	0.133	0.804	0.221
cough_delta_1_mean_std	0.032 [-0.153, 0.218]	0.733	0.838	0.870	0.196	0.076	0.709	0.217
cough_delta_10_mean_mean	-0.038 [-0.226, 0.149]	0.688	0.820	0.860	0.511	0.099	0.620	0.452
cough_delta_10_mean_std	0.007 [-0.153, 0.167]	0.930	0.965	0.971	0.239	0.050	0.688	0.257
cough_delta_11_mean_max	0.119 [-0.065, 0.303]	0.206	0.483	0.508	0.292	0.081	0.736	0.284
cough_delta_11_mean_std	0.146 [-0.083, 0.375]	0.211	0.483	0.508	0.194	0.145	0.737	0.209
cough_delta_12_mean_max	0.286 [0.026, 0.546]	0.031	0.231	0.230	0.311	0.192	0.799	0.280
cough_delta_12_mean_std	0.114 [-0.049, 0.278]	0.170	0.434	0.465	0.149	0.045	0.823	0.154
cough_delta_2_mean_mean	-0.053 [-0.240, 0.135]	0.583	0.733	0.789	0.430	0.089	0.584	0.424
cough_delta_2_mean_std	0.215 [0.005, 0.426]	0.045	0.280	0.297	0.260	0.113	0.810	0.243
cough_delta_3_mean_mean	-0.096 [-0.251, 0.058]	0.223	0.489	0.525	0.499	0.056	0.497	0.501
cough_delta_3_mean_std	0.094 [-0.152, 0.340]	0.456	0.681	0.730	0.102	0.164	0.864	0.105
cough_delta_4_mean_mean	-0.099 [-0.219, 0.022]	0.109	0.386	0.435	0.396	0.020	0.550	0.419
cough_delta_4_mean_std	0.097 [-0.105, 0.299]	0.345	0.605	0.666	0.216	0.098	0.730	0.228
cough_delta_5_mean_mean	-0.154 [-0.394, 0.087]	0.211	0.483	0.508	0.389	0.160	0.763	0.338
cough_delta_5_mean_std	0.129 [-0.164, 0.422]	0.389	0.635	0.706	0.184	0.271	0.734	0.200
cough_delta_6_mean_max	-0.020 [-0.217, 0.178]	0.843	0.925	0.939	0.544	0.096	0.690	0.441
cough_delta_6_mean_mean	-0.088 [-0.373, 0.196]	0.542	0.728	0.778	0.681	0.249	0.578	0.541
cough_delta_6_mean_std	0.120 [-0.073, 0.314]	0.222	0.489	0.525	0.131	0.077	0.815	0.139

CI: Confidence Interval; ICC: Intraclass Correlation Coefficient; $\sigma^2_{\text{intercept}}$: per-patient random intercept variance; σ^2_{slope}: random slope variance for prodromal status; $\sigma^2_{\text{residual}}$: residual variance. All biomarkers were globally standardized (Z-score) prior to LMM fitting. p-value: unadjusted p-value; q-domain: false discovery rate (FDR) adjusted p-value calculated within each domain using the Benjamini–Hochberg procedure. Significance levels were assigned based on q-values: *** $q < 0.001$, ** $q < 0.01$, * $q < 0.05$, and † $q < 0.10$ (exploratory threshold).

Table S5: Physiological, behavioral, and environmental features, evaluated via Linear Mixed Models (LMM) with domain- and global-specific Benjamini–Hochberg False Discovery Rate (FDR) corrections. (cont.)

Domain / Feature	β [95% CI]	p-value	q-dom	q-glob	$\sigma^2_{\text{intercept}}$	σ^2_{slope}	$\sigma^2_{\text{residual}}$	ICC
Acoustic Cough								
cough_delta_7_mean_max	0.124 [-0.039, 0.288]	0.135	0.423	0.446	0.599	0.055	0.653	0.479
cough_delta_7_mean_std	0.143 [0.017, 0.269]	0.026	0.210	0.207	0.219	0.008	0.835	0.208
cough_delta_8_mean_max	0.057 [-0.186, 0.300]	0.646	0.786	0.833	0.399	0.183	0.483	0.452
cough_delta_8_mean_std	0.042 [-0.193, 0.277]	0.725	0.838	0.867	0.225	0.160	0.689	0.246
cough_delta_9_mean_max	0.122 [-0.043, 0.286]	0.147	0.423	0.446	0.286	0.053	0.708	0.288
cough_delta_9_mean_mean	0.003 [-0.129, 0.136]	0.959	0.985	0.988	0.431	0.024	0.660	0.395
cough_delta_9_mean_std	0.201 [0.056, 0.346]	0.007	0.125	0.091†	0.166	0.035	0.715	0.189
cough_duration_max	0.100 [-0.046, 0.246]	0.180	0.449	0.482	0.492	0.050	0.427	0.536
cough_duration_std	0.111 [-0.081, 0.302]	0.258	0.525	0.574	0.408	0.099	0.560	0.421
cough_f0_mean_mean	0.100 [-0.143, 0.342]	0.420	0.656	0.727	0.444	0.165	0.690	0.391
cough_f0_std_max	0.062 [-0.213, 0.337]	0.657	0.791	0.843	0.259	0.226	0.752	0.256
cough_f0_std_mean	-0.089 [-0.358, 0.181]	0.519	0.717	0.765	0.247	0.215	0.769	0.243
cough_f1_mean_max	0.153 [-0.035, 0.341]	0.110	0.386	0.435	0.293	0.067	0.879	0.250
cough_f1_mean_std	0.023 [-0.220, 0.267]	0.851	0.925	0.943	0.120	0.153	0.907	0.117
cough_f2_mean_max	0.142 [0.034, 0.251]	0.010	0.127	0.120	0.431	0.008	0.654	0.397
cough_f2_mean_std	0.023 [-0.141, 0.187]	0.784	0.886	0.910	0.158	0.032	0.862	0.155
cough_f3_mean_max	0.115 [-0.023, 0.254]	0.103	0.383	0.427	0.544	0.018	0.728	0.428
cough_f3_mean_std	0.068 [-0.090, 0.227]	0.396	0.635	0.710	0.109	0.032	0.886	0.109
cough_hnr_mean_max	0.114 [-0.080, 0.309]	0.248	0.514	0.568	0.446	0.093	0.679	0.397
cough_hnr_mean_mean_delta	-0.038 [-0.149, 0.074]	0.509	0.717	0.764	0.000	0.000	1.000	0.000
cough_hnr_mean_mean_lag2	0.018 [-0.080, 0.116]	0.721	0.838	0.866	0.535	0.000	0.613	0.466
cough_hnr_mean_mean_roll_std_7d	0.099 [-0.169, 0.368]	0.468	0.690	0.740	0.268	0.197	0.836	0.243
cough_hnr_mean_std	0.100 [-0.090, 0.290]	0.302	0.576	0.630	0.222	0.077	0.837	0.209
cough_jitter_max	0.232 [0.076, 0.387]	0.004	0.098†	0.057†	0.382	0.041	0.765	0.333
cough_jitter_std	0.076 [-0.081, 0.232]	0.344	0.605	0.666	0.236	0.050	0.634	0.271
cough_kurtosis_std	0.033 [-0.231, 0.297]	0.807	0.895	0.911	0.222	0.206	0.735	0.232
cough_log_energy_mean_std	0.195 [-0.062, 0.452]	0.138	0.423	0.446	0.153	0.180	0.847	0.153
cough_mfcc_0_mean_mean	-0.180 [-0.320, -0.041]	0.011	0.127	0.120	0.711	0.047	0.329	0.684
cough_mfcc_0_mean_std	0.396 [0.118, 0.674]	0.005	0.116	0.075†	0.157	0.219	0.830	0.159
cough_mfcc_0_std_mean	-0.119 [-0.247, 0.008]	0.067	0.310	0.339	0.586	0.031	0.448	0.567
cough_mfcc_0_std_std	0.121 [-0.153, 0.396]	0.386	0.635	0.706	0.200	0.221	0.762	0.207
cough_mfcc_1_mean_max	0.083 [-0.084, 0.249]	0.330	0.605	0.658	0.689	0.075	0.375	0.648
cough_mfcc_1_mean_std	0.207 [0.009, 0.405]	0.041	0.267	0.276	0.157	0.085	0.904	0.148
cough_mfcc_1_std_max	-0.010 [-0.228, 0.208]	0.929	0.965	0.971	0.343	0.134	0.633	0.352
cough_mfcc_1_std_std	0.176 [-0.023, 0.375]	0.083	0.359	0.381	0.098	0.085	0.915	0.097
cough_mfcc_10_mean_max	0.080 [-0.071, 0.231]	0.297	0.576	0.628	0.586	0.050	0.487	0.546
cough_mfcc_10_mean_std	0.216 [-0.080, 0.513]	0.152	0.426	0.449	0.135	0.257	0.849	0.137
cough_mfcc_10_std_mean	-0.180 [-0.423, 0.063]	0.146	0.423	0.446	0.724	0.175	0.690	0.512
cough_mfcc_10_std_std	-0.115 [-0.307, 0.078]	0.244	0.514	0.563	0.116	0.073	0.921	0.112
cough_mfcc_11_mean_max	0.130 [-0.022, 0.283]	0.094	0.376	0.404	0.666	0.056	0.408	0.620
cough_mfcc_11_mean_std	0.282 [-0.088, 0.652]	0.136	0.423	0.446	0.106	0.436	0.874	0.108
cough_mfcc_11_std_mean	-0.120 [-0.245, 0.006]	0.063	0.310	0.339	0.363	0.017	0.758	0.324
cough_mfcc_11_std_std	0.098 [-0.220, 0.417]	0.545	0.728	0.778	0.067	0.309	0.929	0.068
cough_mfcc_12_mean_max	0.249 [0.056, 0.441]	0.011	0.127	0.120	0.590	0.092	0.680	0.465
cough_mfcc_12_mean_std	0.098 [-0.113, 0.308]	0.363	0.625	0.695	0.155	0.102	0.881	0.150
cough_mfcc_12_std_max	-0.064 [-0.302, 0.175]	0.601	0.744	0.800	0.363	0.154	0.717	0.336
cough_mfcc_12_std_std	0.069 [-0.104, 0.242]	0.436	0.668	0.730	0.154	0.050	0.902	0.146
cough_mfcc_2_mean_max	0.212 [0.031, 0.394]	0.022	0.195	0.191	0.727	0.092	0.403	0.643
cough_mfcc_2_mean_std	0.048 [-0.123, 0.219]	0.581	0.733	0.789	0.136	0.045	0.900	0.131
cough_mfcc_2_std_mean	-0.139 [-0.289, 0.011]	0.069	0.310	0.339	0.599	0.055	0.447	0.572
cough_mfcc_2_std_std	0.096 [-0.138, 0.331]	0.422	0.656	0.727	0.111	0.139	0.898	0.110
cough_mfcc_3_mean_mean	0.015 [-0.193, 0.223]	0.889	0.958	0.968	0.745	0.135	0.320	0.700
cough_mfcc_3_mean_std	0.268 [-0.013, 0.549]	0.062	0.310	0.339	0.106	0.215	0.915	0.104
cough_mfcc_3_std_max	0.194 [0.047, 0.342]	0.010	0.127	0.120	0.227	0.033	0.756	0.231
cough_mfcc_3_std_std	0.101 [-0.120, 0.322]	0.370	0.628	0.697	0.074	0.118	0.940	0.073
cough_mfcc_4_mean_mean	-0.121 [-0.249, 0.007]	0.063	0.310	0.339	0.675	0.029	0.473	0.588
cough_mfcc_4_mean_std	0.215 [-0.086, 0.516]	0.161	0.434	0.465	0.222	0.265	0.798	0.218
cough_mfcc_4_std_mean	-0.122 [-0.293, 0.049]	0.163	0.434	0.465	0.510	0.071	0.575	0.470
cough_mfcc_4_std_std	0.002 [-0.197, 0.200]	0.986	0.986	0.993	0.187	0.084	0.878	0.176
cough_mfcc_5_mean_mean	-0.139 [-0.335, 0.058]	0.167	0.434	0.465	1.696	0.109	0.382	0.816
cough_mfcc_5_mean_std	0.261 [0.115, 0.407]	< 0.001	0.053†	0.019*	0.133	0.024	0.873	0.132
cough_mfcc_5_std_max	0.095 [-0.099, 0.288]	0.338	0.605	0.666	0.949	0.075	0.705	0.574
cough_mfcc_5_std_std	-0.002 [-0.175, 0.171]	0.984	0.986	0.993	0.109	0.048	0.917	0.107
cough_mfcc_6_mean_mean	-0.099 [-0.292, 0.093]	0.313	0.585	0.640	0.560	0.103	0.462	0.548
cough_mfcc_6_mean_std	-0.013 [-0.224, 0.199]	0.907	0.965	0.971	0.174	0.095	0.844	0.171
cough_mfcc_6_std_mean	0.019 [-0.379, 0.417]	0.926	0.965	0.971	0.320	0.503	0.770	0.293
cough_mfcc_6_std_std	0.147 [-0.133, 0.428]	0.303	0.576	0.630	0.030	0.219	0.962	0.030
cough_mfcc_7_mean_max	0.044 [-0.114, 0.202]	0.582	0.733	0.789	0.552	0.061	0.455	0.548
cough_mfcc_7_mean_std	0.050 [-0.065, 0.164]	0.397	0.635	0.710	0.256	0.007	0.670	0.277
cough_mfcc_7_std_mean	-0.106 [-0.263, 0.051]	0.187	0.454	0.482	0.640	0.052	0.565	0.531
cough_mfcc_7_std_std	0.048 [-0.119, 0.215]	0.575	0.733	0.789	0.185	0.042	0.856	0.177
cough_mfcc_8_mean_mean	-0.148 [-0.286, -0.010]	0.036	0.251	0.251	0.996	0.049	0.298	0.770
cough_mfcc_8_mean_std	0.313 [0.108, 0.517]	0.003	0.098†	0.047*	0.112	0.094	0.886	0.113
cough_mfcc_8_std_max	0.052 [-0.103, 0.207]	0.512	0.717	0.764	0.637	0.057	0.465	0.578
cough_mfcc_8_std_std	-0.053 [-0.253, 0.147]	0.604	0.744	0.800	0.100	0.081	0.915	0.099
cough_mfcc_9_mean_mean	-0.156 [-0.288, -0.022]	0.022	0.195	0.191	0.414	0.034	0.509	0.448
cough_mfcc_9_mean_std	0.336 [0.129, 0.544]	0.002	0.084†	0.037*	0.166	0.102	0.809	0.170
cough_mfcc_9_std_mean	-0.199 [-0.468, 0.070]	0.147	0.423	0.446	0.295	0.216	0.727	0.289
cough_mfcc_9_std_std	0.062 [-0.151, 0.276]	0.566	0.733	0.789	0.104	0.104	0.875	0.107

CI: Confidence Interval; ICC: Intraclass Correlation Coefficient; $\sigma^2_{\text{intercept}}$: per-patient random intercept variance; σ^2_{slope}: random slope variance for prodromal status; $\sigma^2_{\text{residual}}$: residual variance. All biomarkers were globally standardized (Z-score) prior to LMM fitting. p-value: unadjusted p-value; q-domain: false discovery rate (FDR) adjusted p-value calculated within each domain using the Benjamini–Hochberg procedure. Significance levels were assigned based on q-values: *** $q < 0.001$, ** $q < 0.01$, * $q < 0.05$, and † $q < 0.10$ (exploratory threshold).

Table S5: Physiological, behavioral, and environmental features, evaluated via Linear Mixed Models (LMM) with domain- and global-specific Benjamini–Hochberg False Discovery Rate (FDR) corrections. (cont.)

**Domain / Feature**	β **[95% CI]**	p**-value**	q**-dom**	q**-glob**	$\sigma^2_{\text{intercept}}$	σ^2_{slope}	$\sigma^2_{\text{residual}}$	**ICC**
Acoustic Cough								
cough_shimmer_local_std	0.002 [-0.114, 0.118]	0.974	0.986	0.993	0.276	0.010	0.616	0.309
cough_skewness_max	-0.031 [-0.184, 0.123]	0.697	0.821	0.860	0.346	0.059	0.389	0.470
cough_skewness_std	-0.096 [-0.342, 0.149]	0.442	0.669	0.730	0.338	0.208	0.477	0.415
cough_spec_centroid_mean_mean	-0.054 [-0.214, 0.107]	0.511	0.717	0.764	0.797	0.070	0.362	0.688
cough_spec_centroid_std_mean	-0.153 [-0.284, -0.021]	0.023	0.195	0.191	0.718	0.044	0.363	0.664
cough_spec_centroid_std_std	-0.120 [-0.334, 0.093]	0.268	0.537	0.587	0.396	0.124	0.776	0.338
cough_spec_entropy_mean	-0.201 [-0.415, 0.013]	0.066	0.310	0.339	0.515	0.133	0.510	0.502
cough_spec_entropy_std	0.332 [-0.066, 0.729]	0.102	0.383	0.427	0.221	0.508	0.755	0.227
cough_spec_rolloff_mean_std	0.144 [-0.023, 0.310]	0.091	0.376	0.397	0.146	0.044	0.873	0.143
cough_zcr_mean_std	-0.022 [-0.197, 0.152]	0.804	0.895	0.911	0.171	0.061	0.756	0.185
Indoor Air Quality, Environment								
co2_mean_day	0.148 [-0.049, 0.345]	0.141	0.345	0.446	0.347	0.088	0.766	0.312
co2_min_24h	0.209 [-0.065, 0.483]	0.134	0.345	0.446	0.079	0.170	0.930	0.079
co2_std_night	0.067 [-0.098, 0.233]	0.425	0.585	0.727	0.460	0.058	0.637	0.419
hum_min_24h	0.205 [-0.114, 0.524]	0.208	0.381	0.508	0.262	0.313	0.790	0.249
hum_std_24h	-0.202 [-0.317, -0.087]	0.001	0.013*	0.020*	0.132	0.000	0.896	0.128
hum_std_night	-0.196 [-0.352, -0.040]	0.014	0.103	0.138	0.174	0.034	0.845	0.170
ppm10_max_night	0.224 [-0.060, 0.508]	0.121	0.345	0.446	1.112	0.180	0.809	0.579
ppm10_max_24h	-0.029 [-0.219, 0.161]	0.766	0.802	0.897	1.213	0.072	0.668	0.645
ppm25_min_24h	-0.087 [-0.209, 0.036]	0.167	0.362	0.465	0.359	0.011	0.854	0.296
ppm4_min_day	0.010 [-0.115, 0.135]	0.878	0.878	0.965	0.220	0.011	0.872	0.201
ppm4_min_night	-0.175 [-0.374, 0.024]	0.084	0.308	0.381	0.541	0.115	0.725	0.428
temp_max_night	-0.423 [-0.690, -0.155]	0.002	0.022*	0.041*	0.264	0.204	0.858	0.236
temp_std_night	-0.144 [-0.302, 0.013]	0.073	0.308	0.350	0.591	0.055	0.669	0.469
temp_std_24h	-0.185 [-0.380, 0.010]	0.063	0.308	0.339	0.350	0.092	0.703	0.333
voc_max_day	-0.039 [-0.205, 0.126]	0.641	0.762	0.831	0.164	0.053	0.917	0.152
voc_mean_night	-0.104 [-0.340, 0.131]	0.386	0.567	0.706	0.822	0.142	0.807	0.505
voc_mean_24h	-0.050 [-0.211, 0.111]	0.542	0.702	0.778	0.083	0.028	0.958	0.080
voc_min_day	0.098 [-0.045, 0.241]	0.181	0.362	0.482	0.079	0.016	0.912	0.080
voc_min_night	-0.118 [-0.356, 0.120]	0.330	0.519	0.658	1.985	0.204	0.865	0.697
voc_min_24h	0.072 [-0.283, 0.427]	0.691	0.762	0.860	2.312	0.051	0.838	0.734
voc_std_night	0.033 [-0.130, 0.196]	0.693	0.762	0.860	0.165	0.043	0.878	0.158
voc_std_24h	-0.087 [-0.241, 0.068]	0.273	0.462	0.592	0.085	0.028	0.915	0.085

CI: Confidence Interval; ICC: Intraclass Correlation Coefficient; $\sigma^2_{\text{intercept}}$: per-patient random intercept variance; σ^2_{slope}: random slope variance for prodromal status; $\sigma^2_{\text{residual}}$: residual variance. All biomarkers were globally standardized (Z-score) prior to LMM fitting. p-value: unadjusted p-value; q-domain: false discovery rate (FDR) adjusted p-value calculated within each domain using the Benjamini–Hochberg procedure. Significance levels were assigned based on q-values: *** $q < 0.001$, ** $q < 0.01$, * $q < 0.05$, and † $q < 0.10$ (exploratory threshold).
