# Supplementary material for: Multimodal Prodromal Patterns Associated with COPD Exacerbations in Home Telemonitoring: An Exploratory Observational Study
Source: Bioengineering (Basel). 2026 Oct 14;13(10):1195. doi: 10.3390/bioengineering13101195 (PMC13701270; doi:10.3390/bioengineering13101195)
Supplement: Supplementary file 1 [file bioengineering-13-01195-s001.zip › Supplementary Material S6.pdf]

Supplementary Material S6

Table S6: Comparison of LMMs before and after adjustment for season fixed effects, reporting standardized β coefficients, 95% CIs, raw p-values, and domain- and global-specific false discovery rate (FDR) q-values across all features.

**Domain**	**Biomarker**	**Unadjusted Model**					**Model Adjusted by Season**				
		β	IC 95%	p-val	q-dom	q-glob	β	IC 95%	p-val	q-dom	q-glob
Patient Reported Symptoms	q1_general_health	0.390	[0.172, 0.608]	< 0.001	0.002**	0.019*	0.380	[0.172, 0.588]	< 0.001	0.001**	0.014*
	q2_dyspnea_roll_mean_3d	0.271	[0.033, 0.509]	0.025	0.040*	0.207	0.278	[0.057, 0.499]	0.014	0.021*	0.144
	q3_cough_delta	0.222	[0.056, 0.388]	0.009	0.016*	0.115	0.224	[0.058, 0.390]	0.008	0.015*	0.117
	q3_cough_roll_mean_3d	0.344	[0.196, 0.493]	< 0.001	< 0.001***	0.001**	0.327	[0.178, 0.475]	< 0.001	< 0.001***	0.002**
	q3_cough_roll_std_7d	0.563	[0.218, 0.909]	0.001	0.003**	0.037*	0.548	[0.187, 0.910]	0.003	0.007**	0.078†
	q4_rescue_inhaler_roll_std_7d	0.313	[-0.016, 0.641]	0.062	0.087†	0.339	0.325	[-0.045, 0.695]	0.085	0.119	0.410
	q5_sputum_volume_delta	0.232	[0.095, 0.369]	0.001	0.003**	0.027*	0.233	[0.095, 0.371]	0.001	0.003**	0.033*
	q5_sputum_volume_roll_mean_3d	0.249	[0.135, 0.364]	< 0.001	< 0.001***	0.002**	0.238	[0.136, 0.339]	< 0.001	< 0.001***	0.001**
	q5_sputum_volume_roll_std_7d	0.635	[0.240, 1.031]	0.002	0.003**	0.037*	0.622	[0.191, 1.054]	0.005	0.009**	0.083†
	q6_sputum_color	0.281	[0.147, 0.415]	< 0.001	< 0.001***	0.002**	0.275	[0.140, 0.410]	< 0.001	< 0.001***	0.004**
Skin Temperature	relative_skin_temp	0.149	[-0.021, 0.319]	0.086	0.086†	0.385	0.147	[-0.027, 0.321]	0.097	0.097†	0.440
Cardiovascular	resting_hr	0.140	[0.031, 0.249]	0.012	0.059†	0.121	0.125	[0.018, 0.231]	0.022	0.045*	0.198
	resting_hr_delta	0.158	[0.002, 0.315]	0.047	0.078†	0.301	0.161	[0.024, 0.298]	0.022	0.045*	0.198
	resting_hr_roll_std_7d	0.477	[0.053, 0.900]	0.027	0.068†	0.209	0.478	[0.055, 0.901]	0.027	0.045*	0.205
Acoustic - Cough	cough_jitter_max	0.232	[0.076, 0.387]	0.004	0.098†	0.057†	0.205	[0.048, 0.361]	0.011	0.126	0.129
	cough_mfcc_5_mean_std	0.261	[0.115, 0.407]	< 0.001	0.053†	0.019*	0.279	[0.134, 0.423]	< 0.001	0.018*	0.008**
	cough_mfcc_8_mean_std	0.313	[0.109, 0.517]	0.003	0.098†	0.047*	0.295	[0.091, 0.498]	0.005	0.126	0.083†
	cough_mfcc_9_mean_std	0.336	[0.129, 0.544]	0.002	0.084†	0.037*	0.341	[0.128, 0.553]	0.002	0.095†	0.052†
Acoustic - Speech	speech_mfcc_6_mean	-0.196	[-0.323, -0.070]	0.002	0.059†	0.045*	-0.192	[-0.320, -0.065]	0.003	0.067†	0.078†
	speech_mfcc_8_mean	-0.272	[-0.460, -0.085]	0.004	0.073†	0.067†	-0.275	[-0.462, -0.088]	0.004	0.067†	0.083†
	speech_intensity_var	0.123	[0.072, 0.174]	< 0.001	< 0.001***	0.001**	0.107	[0.055, 0.158]	< 0.001	0.003**	0.004**
Indoor Air Quality, Environment	hum_std_24h	-0.202	[-0.317, -0.087]	0.001	0.013*	0.020*	-0.117	[-0.235, 0.001]	0.052	0.384	0.305
	temp_max_night	-0.423	[-0.690, -0.155]	0.002	0.022*	0.041*	-0.094	[-0.186, -0.001]	0.047	0.384	0.290

All continuous features were globally standardized (Z-score) prior to model fitting. β: standardized fixed-effect slope coefficient for the prodromal status (7-day pre-exacerbation window vs. baseline); 95% CI: 95% Confidence Interval; p-val: unadjusted p-value; q-dom: False Discovery Rate (FDR) adjusted p-value calculated within each physiological domain using the Benjamini–Hochberg procedure; q-glob: FDR-adjusted p-value calculated across all evaluated features globally. Only features exhibiting statistical significance at an exploratory threshold of q-dom < 0.10 in either model are displayed. Significance levels are assigned based on q-values: *** $q < 0.001$, ** $q < 0.01$, * $q < 0.05$, and † $q < 0.10$ (exploratory threshold).
