# Supplementary material for: Multimodal Prodromal Patterns Associated with COPD Exacerbations in Home Telemonitoring: An Exploratory Observational Study
Source: Bioengineering (Basel). 2026 Oct 14;13(10):1195. doi: 10.3390/bioengineering13101195 (PMC13701270; doi:10.3390/bioengineering13101195)
Supplement: Supplementary file 1 [file bioengineering-13-01195-s001.zip › Supplementary Material S7.pdf]

Supplementary Material S7

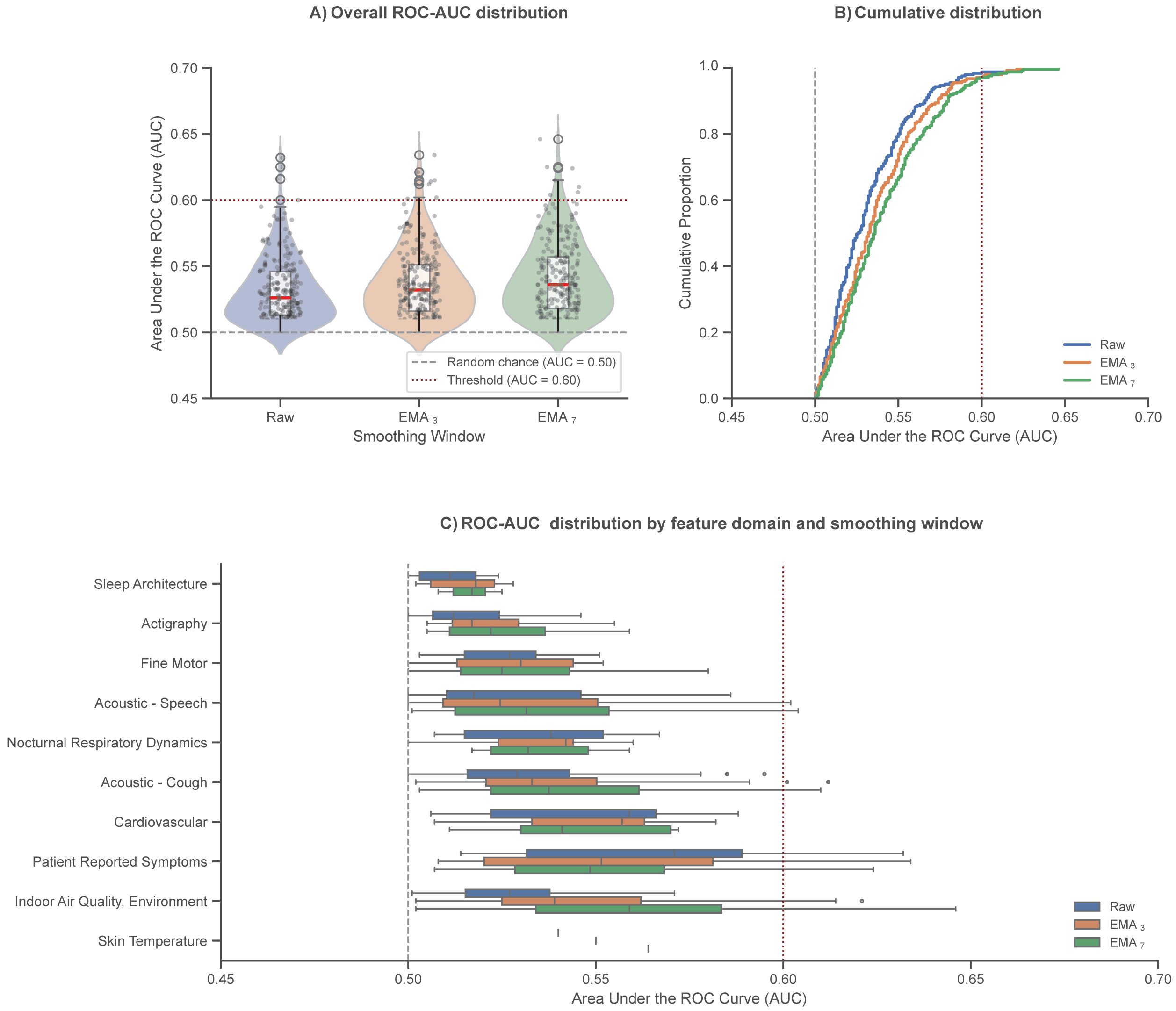

Figure S7: Distribution of Area Under the ROC Curve (ROC-AUC) across all markers and temporal smoothing windows. (A) Distribution AUC-ROC for all $N = 245$ evaluated features under cross-sectional (raw) and exponentially smoothed (EMA_3 and EMA_7) formulations. Violin plots depict probability density, boxplots display median and interquartile ranges (IQR); red central lines represent median values; and individual dots represent individual features. (B) Empirical Cumulative Distribution Function (ECDF) illustrating the cumulative proportion of features achieving specific AUC values across temporal windows. (C) Domain-stratified ROC-AUC distributions across smoothing windows, ordered by median predictive performance under EMA_7. Dotted horizontal/vertical red lines indicate the threshold ($AUC = 0.60$) and dashed gray lines mark random classification chance ($AUC = 0.50$).
