# Supplementary material for: Multimodal Prodromal Patterns Associated with COPD Exacerbations in Home Telemonitoring: An Exploratory Observational Study
Source: Bioengineering (Basel). 2026 Oct 14;13(10):1195. doi: 10.3390/bioengineering13101195 (PMC13701270; doi:10.3390/bioengineering13101195)
Supplement: Supplementary file 1 [file bioengineering-13-01195-s001.zip › Supplementary Material S8.pdf]

Supplementary Material S8

Table S8-A: Sensitivity Analysis: feature selection stability after the correlation analysis between primary (symptom-based and event-based) and sensitivity (only event-based) exacerbation criteria across all explored domains.

**Domain**	**Primary Analysis**	**Sensitivity Analysis**	**Overlap (N)**	**Stability (%)**
Actigraphy	12	12	11	91.7%
Cardiovascular	5	5	5	100.0%
Nocturnal Respiratory Dynamics	5	5	5	100.0%
Sleep Architecture	7	7	7	100.0%
Skin Temperature	1	1	1	100.0%
Patient-Reported Symptoms	14	15	12	85.7%
Fine Motor Skills	17	17	17	100.0%
Acoustic: Speech	50	48	43	86.0%
Acoustic: Cough	112	113	94	83.9%
Indoor Air Quality, Environment	22	22	15	68.2%
**TOTAL**	**245**	**245**	**210**	**85.7%**

Table S8-B: Sensitivity Analysis: features exhibiting statistically significant shifts between stable and prodromal phases. Only data associated to event-based exacerbations were evaluated via paired Wilcoxon signed-rank tests with domain-specific Benjamini–Hochberg False Discovery Rate (FDR) corrections.

**Domain / Feature**	**Stable**	**Prodromal**	**Delta [95% CI]**	**CLES [95% CI]**	***p*-value**	***q*-value**
Patient Reported Symptoms						
General health status (q1_general_health)	2.438 ± 0.665	2.805 ± 0.823	$+0.367$ [$+0.147, +0.587$]	0.691 [0.589, 0.832]	0.002	0.017*
Dyspnea 3-day rolling mean (q2_dyspnea_roll_mean_3d)	2.628 ± 0.867	2.977 ± 1.081	$+0.349$ [$+0.134, +0.563$]	0.668 [0.587, 0.821]	< 0.001	0.004**
Cough freq. (q3_cough)	1.791 ± 0.862	2.231 ± 0.931	$+0.440$ [$+0.082, +0.798$]	0.648 [0.536, 0.791]	0.009	0.032*
Cough freq. at 2-day lag (q3_cough_lag2)	1.787 ± 0.854	2.129 ± 0.980	$+0.342$ [$-0.016, +0.699$]	0.607 [0.485, 0.765]	0.042	0.070†
Delta of rescue inhaler (q4_rescue_inhaler_delta)	0.004 ± 0.006	-0.009 ± 0.023	-0.013 [$-0.026, +0.001$]	0.276 [0.138, 0.431]	0.036	0.067†
Rescue inhaler at 2-day lag (q4_rescue_inhaler_lag2)	2.272 ± 0.793	2.527 ± 0.735	$+0.255$ [$+0.067, +0.442$]	0.679 [0.594, 0.816]	0.003	0.017*
Rescue inhaler 7-day rolling Std.dev. (q4_rescue_inhaler_roll_std_7d)	0.097 ± 0.101	0.028 ± 0.051	-0.069 [$-0.120, -0.018$]	0.250 [0.112, 0.372]	0.013	0.032*
Sputum volume 3-day rolling mean (q5_sputum_volume_roll_mean_3d)	1.622 ± 0.819	2.040 ± 1.068	$+0.418$ [$+0.046, +0.790$]	0.597 [0.469, 0.760]	0.023	0.049*
Sputum purulence (q6_sputum_color)	0.533 ± 0.530	0.949 ± 0.695	$+0.416$ [$+0.096, +0.736$]	0.663 [0.543, 0.816]	0.013	0.032*
Cardiovascular						
Resting heart rate (resting_hr)	67.598 ± 8.728	69.422 ± 9.521	$+1.824$ [$+0.712, +2.937$]	0.561 [0.510, 0.653]	0.001	0.006**
Delta of resting heart rate (resting_hr_delta)	-0.011 ± 0.109	0.329 ± 0.501	$+0.339$ [$+0.039, +0.639$]	0.765 [0.531, 0.934]	0.035	0.088†
Nocturnal Respiratory Dynamics						
Mean nocturnal SpO2 (spo2_mean)	94.237 ± 1.623	93.403 ± 2.239	-0.834 [$-1.504, -0.164$]	0.388 [0.265, 0.480]	0.013	0.067†
Acoustic - Cough						
Maximum of mean cough F3 (cough_f3_mean_max)	2560.466 ± 181.563	2666.530 ± 184.070	$+106.064$ [$+60.151, +151.976$]	0.681 [0.618, 0.799]	< 0.001	0.055†
Mean of Stds. of cough MFCC-1 (cough_mfcc_1_std_mean)	98.433 ± 10.017	92.419 ± 9.617	-6.014 [$-9.395, -2.633$]	0.292 [0.153, 0.368]	0.002	0.092†
Mean of Stds. of cough MFCC-2 (cough_mfcc_2_std_mean)	29.227 ± 3.466	26.529 ± 3.780	-2.698 [$-4.103, -1.293$]	0.312 [0.181, 0.361]	< 0.001	0.055†

Data are reported as mean ± standard deviation across subjects, derived from per-patient averages calculated for each clinical phase. Stable: baseline monitoring periods; Prodromal: risk windows immediately preceding a validated event-based exacerbation; Delta: absolute difference computed as $\text{mean}_{\text{prodromal}} - \text{mean}_{\text{stable}}$, presented with its 95% confidence interval [95% CI]; CLES: common language effect size, computed as $P(\text{Prodromal} > \text{Stable})$, presented with its 95% bootstrap confidence interval [95% CI]. Values > 0.50 indicate an increase in the prodromal phase, whereas values < 0.50 indicate a decrease. Statistical significance was assessed using two-sided paired Wilcoxon signed-rank tests. p-value: unadjusted p-value; q-value: False Discovery Rate (FDR) adjusted p-value calculated within each physiological domain using the Benjamini–Hochberg procedure. Significance levels were assigned based on q-values: ** $q < 0.01$, * $q < 0.05$, and † $q < 0.10$ (exploratory threshold).

Table S8-C: Sensitivity Analysis: Linear mixed-effects model (LMM) performance after adjustment for season fixed effects across validated monitoring domains and event-based exacerbations. Domain-specific Benjamini–Hochberg False Discovery Rate (FDR) corrections were applied. Only features with statistical significance (q-domain < 0.1) are shown.

**Domain / Feature**	β **[95% CI]**	p**-value**	q**-dom**	q**-glob**	$\sigma^2_{\text{intercept}}$	σ^2_{slope}	$\sigma^2_{\text{residual}}$	**ICC**
Patient Reported Symptoms								
General health status (q1 general health)	0.497 [0.223, 0.771]	< 0.001	0.003**	0.015*	0.794	0.250	0.281	0.739
Dyspnea 3-day rolling mean (q2 dyspnea roll mean 3d)	0.412 [0.188, 0.636]	< 0.001	0.003**	0.015*	0.950	0.167	0.244	0.795
Cough freq. (q3 cough)	0.408 [0.075, 0.742]	0.016	0.041*	0.134	0.855	0.369	0.310	0.734
Delta of cough (q3 cough delta)	0.146 [0.011, 0.281]	0.034	0.072†	0.205	0.000	0.002	1.000	0.000
Rescue inhaler 2-day lag (q4_rescue_inhaler_lag2)	0.253 [0.087, 0.419]	0.003	0.014*	0.049*	0.804	0.082	0.216	0.789
Rescue inhaler 7-day rolling Std (q4_rescue_inhaler_roll_std_7d)	-0.240 [-0.417,-0.062]	0.008	0.030*	0.099†	0.205	0.071	0.775	0.209
Sputum volume 3-day rolling mean (q5_sputum_volume_roll_mean_3d)	0.438 [0.085, 0.791]	0.015	0.041*	0.134	0.842	0.424	0.241	0.778
Cardiovascular								
Resting heart rate (resting_hr)	0.166 [0.065, 0.267]	0.001	0.006**	0.035*	0.731	0.028	0.106	0.874
Delta of resting heart rate (resting_hr_delta)	0.223 [0.067, 0.379]	0.005	0.013*	0.071†	0.000	0.011	0.998	0.000
Nocturnal Respiratory Dynamics								
Nocturnal mean SpO2 (spo2_mean)	-0.330 [-0.560, -0.099]	0.005	0.025*	0.071†	0.377	0.136	0.637	0.372
Acoustic - Speech								
Mean speech MFCC 8 (speech_mfcc_8_mean)	-0.294 [-0.492, -0.097]	0.004	0.085†	0.054†	0.949	0.083	0.410	0.698
Std.dev. of speech RMS amplitude (speech_intensity_var)	0.136 [0.045, 0.227]	0.003	0.085†	0.054†	0.913	0.013	0.156	0.854
Acoustic - Cough								
Mean of means of Δ MFCC 4 (cough_delta_4_mean_mean)	-0.263 [-0.430, -0.096]	0.002	0.029*	0.045*	0.428	0.034	0.572	0.428
Std. of means of Δ MFCC 6 (cough_delta_6_mean_std)	0.412 [0.143, 0.682]	0.003	0.031*	0.049*	0.122	0.148	0.832	0.128
Maximum of means of F3 (cough_f3_mean_max)	0.323 [0.172, 0.473]	< 0.001	0.001**	0.003**	0.463	0.008	0.722	0.391
Mean of Stds. of MFCC 1 (cough_mfcc_1_std_mean)	-0.322 [-0.491, -0.154]	< 0.001	0.005**	0.011*	0.571	0.052	0.440	0.565
Mean of Stds. of MFCC 2 (cough_mfcc_2_std_mean)	-0.419 [-0.631, -0.206]	< 0.001	0.004**	0.010**	0.410	0.083	0.591	0.410
Maximum of means of MFCC 13 (cough_mfcc_13_mean_max)	0.393 [0.226, 0.560]	< 0.001	< 0.001***	0.001**	0.495	0.031	0.668	0.425
Mean of means of MFCC 5 (cough_mfcc_5_mean_mean)	-0.205 [-0.339, -0.071]	0.003	0.031*	0.049*	0.605	0.018	0.399	0.603
Std. of means of cough MFCC-6 (cough_mfcc_6_mean_std)	0.359 [0.151, 0.566]	0.001	0.015*	0.024*	0.142	0.061	0.873	0.140
Std. of Stds. of MFCC 7 (cough_mfcc_7_std_std)	0.415 [0.152, 0.677]	0.002	0.029*	0.045*	0.027	0.122	0.963	0.027
Std. of means cough MFCC-10 (cough_mfcc_10_mean_std)	0.345 [0.143, 0.546]	0.001	0.015*	0.024*	0.160	0.054	0.814	0.164

CI: Confidence Interval; ICC: Intraclass Correlation Coefficient; $\sigma^2_{\text{intercept}}$: per-patient random intercept variance; σ^2_{slope}: random slope variance for prodromal status; $\sigma^2_{\text{residual}}$: residual variance. All biomarkers were globally standardized (Z-score) prior to LMM fitting. p-value: unadjusted p-value; q-dom: false discovery rate (FDR) adjusted p-value calculated within each physiological domain using the Benjamini–Hochberg procedure. q-glob: FDR adjusted p-value calculated in all features using the Benjamini–Hochberg procedure. Significance levels were assigned based on q-values: *** $q < 0.001$, ** $q < 0.01$, * $q < 0.05$, and † $q < 0.10$ (exploratory threshold).

Table S8-D: Sensitivity Analysis: univariate area under the receiver operating characteristic curve (ROC-AUC) across domains when restricting the endpoint to event-based exacerbations.

Domain / Feature	Raw AUC [95% CI]	EMA_3 AUC [95% CI]	EMA_7 AUC [95% CI]
Patient-Reported Symptoms			
q1_general_health	0.671 [0.638, 0.704]	0.661 [0.627, 0.694]	0.648 [0.614, 0.682]
q3_cough	0.664 [0.630, 0.698]	0.674 [0.642, 0.707]	0.673 [0.640, 0.707]
q2_dyspnea_roll_mean_3d	0.653 [0.618, 0.687]	0.653 [0.620, 0.687]	0.648 [0.614, 0.681]
q3_cough_lag2	0.641 [0.607, 0.675]	0.652 [0.619, 0.686]	0.662 [0.628, 0.697]
q4_rescue_inhaler_lag2	0.633 [0.602, 0.664]	0.641 [0.609, 0.673]	0.647 [0.615, 0.678]
q3_cough_delta	0.619 [0.579, 0.659]	0.530 [0.488, 0.572]	0.512 [0.470, 0.553]
q5_sputum_volume_roll_mean_3d	0.594 [0.553, 0.635]	0.604 [0.564, 0.645]	0.621 [0.581, 0.661]
q5_sputum_volume_roll_std_7d	0.588 [0.545, 0.630]	0.595 [0.552, 0.637]	0.603 [0.561, 0.645]
q5_sputum_volume_delta	0.586 [0.545, 0.627]	0.589 [0.546, 0.632]	0.620 [0.579, 0.661]
q6_sputum_color	0.573 [0.535, 0.611]	0.587 [0.549, 0.626]	0.602 [0.565, 0.640]
Nocturnal Respiratory Dynamics			
spo2_mean	0.581 [0.538, 0.625]	0.596 [0.552, 0.640]	0.601 [0.557, 0.644]
Cardiovascular			
resting_hr	0.618 [0.580, 0.655]	0.607 [0.569, 0.645]	0.596 [0.558, 0.634]
Acoustic - Speech			
speech_mean_intensity	0.615 [0.576, 0.653]	0.642 [0.602, 0.683]	0.648 [0.605, 0.691]
speech_intensity_var	0.603 [0.565, 0.640]	0.626 [0.588, 0.665]	0.629 [0.588, 0.671]
speech_mfcc_8_mean	0.600 [0.558, 0.641]	0.618 [0.575, 0.660]	0.624 [0.581, 0.667]
speech_mean_intensity_lag1	0.594 [0.554, 0.634]	0.621 [0.579, 0.662]	0.626 [0.582, 0.670]
speech_mfcc_2_mean	0.591 [0.551, 0.631]	0.607 [0.567, 0.646]	0.618 [0.578, 0.658]
speech_total_pause_dur_lag2	0.585 [0.545, 0.625]	0.605 [0.566, 0.643]	0.620 [0.581, 0.658]
speech_total_pause_dur_lag1	0.585 [0.545, 0.625]	0.605 [0.566, 0.643]	0.624 [0.585, 0.662]
speech_mean_intensity_lag2	0.584 [0.542, 0.625]	0.605 [0.561, 0.648]	0.608 [0.563, 0.653]
speech_rhythm	0.583 [0.544, 0.621]	0.606 [0.566, 0.646]	0.619 [0.577, 0.662]
speech_pause_count_lag2	0.575 [0.537, 0.614]	0.599 [0.562, 0.637]	0.621 [0.583, 0.658]
speech_pause_count_lag1	0.573 [0.534, 0.612]	0.600 [0.562, 0.638]	0.625 [0.587, 0.662]
speech_rhythm_var	0.562 [0.514, 0.611]	0.592 [0.544, 0.640]	0.602 [0.555, 0.650]
Acoustic - Cough			
cough_mfcc_3_std_mean	0.621 [0.580, 0.661]	0.619 [0.578, 0.660]	0.602 [0.560, 0.643]
cough_mfcc_13_mean_max	0.620 [0.581, 0.658]	0.627 [0.590, 0.663]	0.613 [0.577, 0.650]
cough_mfcc_1_std_mean	0.619 [0.579, 0.658]	0.638 [0.599, 0.677]	0.629 [0.589, 0.668]
cough_mfcc_6_mean_std	0.613 [0.575, 0.651]	0.613 [0.574, 0.652]	0.604 [0.565, 0.643]
cough_mfcc_10_mean_std	0.606 [0.567, 0.644]	0.601 [0.563, 0.639]	0.586 [0.549, 0.623]
cough_mfcc_2_std_mean	0.599 [0.555, 0.643]	0.612 [0.568, 0.656]	0.609 [0.565, 0.654]
cough_f3_mean_std	0.595 [0.556, 0.633]	0.611 [0.570, 0.651]	0.607 [0.564, 0.649]
cough_mfcc_5_mean_mean	0.593 [0.549, 0.636]	0.601 [0.558, 0.644]	0.594 [0.552, 0.635]
cough_delta_7_mean_std	0.592 [0.550, 0.633]	0.610 [0.570, 0.649]	0.634 [0.597, 0.672]
cough_delta_5_mean_mean	0.591 [0.549, 0.633]	0.601 [0.560, 0.642]	0.608 [0.569, 0.648]
cough_f3_mean_max	0.589 [0.546, 0.632]	0.602 [0.561, 0.644]	0.596 [0.556, 0.635]
cough_mfcc_2_mean_std	0.580 [0.541, 0.619]	0.608 [0.571, 0.646]	0.634 [0.596, 0.672]
cough_band_energy_ratio_max	0.575 [0.533, 0.616]	0.595 [0.553, 0.636]	0.612 [0.571, 0.653]
cough_delta_3_mean_std	0.568 [0.526, 0.609]	0.585 [0.544, 0.626]	0.606 [0.566, 0.645]
cough_delta_9_mean_max	0.555 [0.513, 0.596]	0.590 [0.547, 0.631]	0.620 [0.580, 0.660]
Indoor Air Quality, Environment			
temp_mean_24h	0.603 [0.567, 0.639]	0.607 [0.571, 0.644]	0.608 [0.571, 0.645]
co2_min_day	0.568 [0.526, 0.609]	0.605 [0.563, 0.647]	0.637 [0.595, 0.680]
voc_min_day	0.547 [0.508, 0.587]	0.590 [0.551, 0.629]	0.630 [0.592, 0.667]
voc_min_24h	0.534 [0.494, 0.574]	0.562 [0.521, 0.602]	0.602 [0.563, 0.640]

ROC-AUC values represent individual univariate discriminative capacities computed independently for each biomarker across all longitudinal observations, without prior multivariate feature selection.
